# SeqTools: visual tools for manual analysis of sequence alignments

**DOI:** 10.1186/s13104-016-1847-3

**Published:** 2016-01-22

**Authors:** Gemma Barson, Ed Griffiths

**Affiliations:** Wellcome Trust Sanger Institute, Wellcome Trust Genome Campus, Hinxton, Cambridge, UK

**Keywords:** Sequence, Alignment, Annotation, Curation, Protein, Nucleotide, DNA

## Abstract

**Background:**

Manual annotation is essential to create high-quality reference alignments and annotation. Annotators need to be able to view sequence alignments in detail. The SeqTools package provides three tools for viewing different types of sequence alignment: Blixem is a many-to-one browser of pairwise alignments, displaying multiple match sequences aligned against a single reference sequence; Dotter provides a graphical dot-plot view of a single pairwise alignment; and Belvu is a multiple sequence alignment viewer, editor, and phylogenetic tool. These tools were originally part of the AceDB genome database system but have been completely rewritten to make them generally available as a standalone package of greatly improved function.

**Findings:**

Blixem is used by annotators to give a detailed view of the evidence for particular gene models. Blixem displays the gene model positions and the match sequences aligned against the genomic reference sequence. Annotators use this for many reasons, including to check the quality of an alignment, to find missing/misaligned sequence and to identify splice sites and polyA sites and signals. Dotter is used to give a dot-plot representation of a particular pairwise alignment. This is used to identify sequence that is not represented (or is misrepresented) and to quickly compare annotated gene models with transcriptional and protein evidence that putatively supports them. Belvu is used to analyse conservation patterns in multiple sequence alignments and to perform a combination of manual and automatic processing of the alignment. High-quality reference alignments are essential if they are to be used as a starting point for further automatic alignment generation.

**Conclusions:**

While there are many different alignment tools available, the SeqTools package provides unique functionality that annotators have found to be essential for analysing sequence alignments as part of the manual annotation process.

**Electronic supplementary material:**

The online version of this article (doi:10.1186/s13104-016-1847-3) contains supplementary material, which is available to authorized users.

## Findings

### Background

Manual annotation is essential to create high-quality reference alignments and annotation. Annotators need to be able to view sequence alignments in detail in order to verify them and to correct any errors. The SeqTools package provides three interactive tools for viewing different types of sequence alignment: Blixem, Dotter and Belvu.

These three programs were originally part of the AceDB [1] genome database system and as such were used mostly by vertebrate annotation groups at the Sanger Institute. The SeqTools package comprises programs derived from AceDB but that are a complete and independent rewrite. The package offers support for new feature types and file formats, making it more widely useful and compatible with other tools. Significant enhancements have also been added since the programs were first published [2–4].

### Implementation

The SeqTools programs are desktop applications written in C using GTK+. They are easy to install and are fast to run for small to medium sized alignments. Unlike many web-based tools, they do not require a network connection to work so are not subject to network problems. They support simple, widely-supported text-based file formats and have extensive command-line options which enable their use in software pipelines.

#### Blixem

Blixem is a many-to-one browser of pairwise alignments. It takes two files as input: a FASTA file containing the reference sequence, and a GFF version 3 file containing alignments as well as any gene models or other features of interest. Input files can be passed on the command line or piped in and additional feature files can be loaded from within the program. The move to GFF from legacy AceDB file formats is a major improvement in version 4 of Blixem.

The display consists of two sections: a zoomable overview section showing the feature positions along the reference sequence; and a detail section showing the actual alignment of protein or nucleotide sequences to the reference DNA sequence. Multiple match sequences are stacked below the reference sequence and individual bases are colour-coded to indicated how well they match. Markers indicate where deletions and insertions occur.

**Fig. 1:**
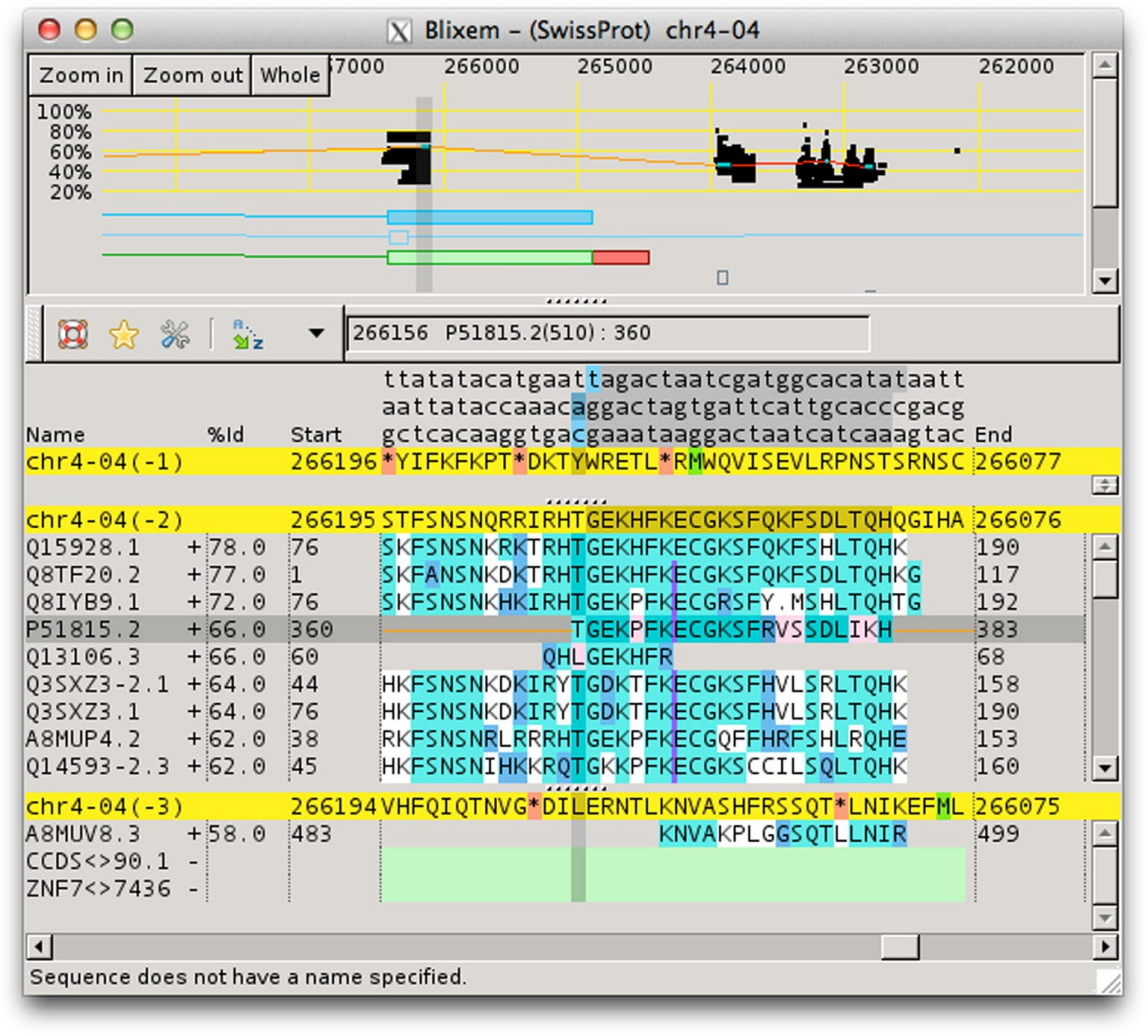
Blixem in protein mode. Blixem showing SwissProt alignments for human chromosome 4. The three-frame translation of the reference sequence is shown in *yellow*. The match sequence residues are highlighted in *cyan* for an exact match, *blue* for a conserved match and *grey* for a mismatch. Insertions are indicated by a *vertical purple bar* and deletions by a *dot*

Blixem works in nucleotide or protein mode depending on the type of the sequences. In nucleotide mode, both strands of the reference sequence are calculated and can be displayed simultaneously in the detail section. In protein mode, the three-frame translation of the reference sequence is calculated and all three frames for the current strand can be shown (Fig. 1). In both modes, the user can easily toggle the display between the forward and reverse strand orientation.

Blixem can display gene models and other feature types. Gene models and basic features are shown in the overview section as well as the detail section, and CDS and UTR sections can be highlighted in different colours. In version 4, support for new feature types has been added. These include: short-reads, which are displayed aligned against the reference sequence; SNPs, insertions and deletions, which are highlighted in the reference sequence; annotated polyA sites and signals, which again are highlighted in the reference sequence; and polyA tails, which are shown in the detail-view. Any other feature type can also be loaded, and will be displayed with simple positional information along with any styles specified in the config file.

An improved user-interface makes it easy to quickly find and navigate around alignments. There are flexible methods to filter, highlight and sort sequences. Other new features allow users to display a coverage plot, to display colinearity lines between alignment blocks, to show unaligned portions of match sequences, and to highlight splice-sites in the reference sequence for particular features.

Blixem has many configuration and command-line options, which include functionality to map coordinates, zoom in to a particular region, specify the sort order, and toggle sections of the display on or off. The colour scheme can be specified in an optional “.ini”-like styles file to match local colour usage. When calling Blixem from another program, the display can therefore be customised to match that of the parent program as closely as possible.

Blixem can be used to display next-generation sequencing (NGS) data. It is capable of displaying up to around 50,000 reads while remaining usefully responsive. Startup time is around 20 s with this number of reads, and while a small lag is noticeable it is less than a second for most operations. Blixem doesn’t currently read BAM/SAM files natively, so the reads have to be provided in GFF3 format. However, we plan to incorporate HTSlib into Blixem to provide native support for BAM/SAM in the future. Blixem has a number of display settings for working with NGS data. A configuration option (“squash-identical-matches”) allows the user to toggle a “squashed” view, where identical reads are compressed onto the same line, and lines are annotated with the number of reads they contain. This configuration option can be applied globally, or just to specific sources. There are command-line arguments to enable the squashed view by default, to show the coverage plot by default, and to change the default sort order (e.g. to sort by position or identity rather than name).

Dotter can be called from within Blixem to give a graphical representation of a particular alignment, which often allows annotators to see matches they would otherwise have missed.

#### Dotter

Dotter is a graphical dot-matrix program for detailed comparison of two sequences. Dotter can be called from the command-line or from other tools such as Blixem. It takes two sequences as input, in FASTA file format. Every residue in one sequence is compared to every residue in the other and a score is calculated. The scores are plotted on a grid with one sequence on the x-axis and the other on the y-axis. Dots are drawn in grey-scale, with darker shades indicating a higher score. Interactive function allows exploration of score values regardless of screen display bias.

**Fig. 2:**
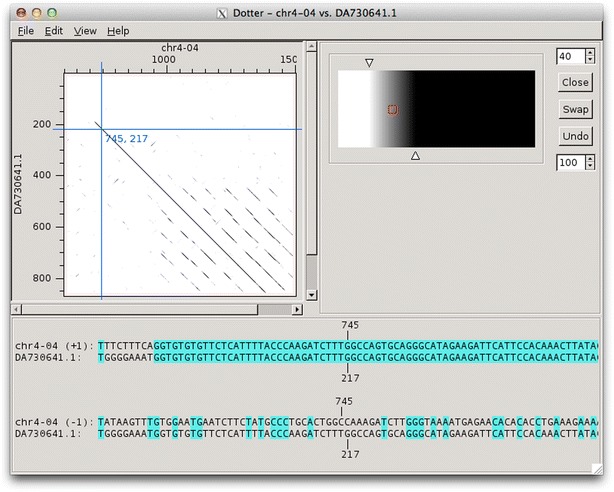
Dotter in nucleotide mode. *Top left* the dot-plot. *Top-right* the grey-ramp tool, used for dynamically adjusting the stringency cut-offs. *Bottom* the alignment tool, showing the sequence data at the current cross-hair position; both strands of the horizontal sequence are displayed, and residues are coloured according to how well they match

Dotter can operate in nucleotide–nucleotide, nucleotide–protein or protein–protein mode. In nucleotide–nucleotide mode, comparisons are made against both strands of the horizontal sequence; alignments appear as diagonal lines, with alignments on the reverse strand sloping in the opposite direction to those on the forward strand (Fig. 2). In nucleotide–protein mode, the three-frame translation of the horizontal sequence is computed and alignments against all three frames are shown. In protein–protein mode Dotter just performs a direct comparison between the two sequences.

To improve visualisation, scores are averaged over a sliding window. Scores below a minimum cutoff are ignored and scores above a maximum cutoff are saturated. The stringency cutoffs can be adjusted interactively using the Greyramp Tool, without having to recalculate the dot-matrix. This is a major advantage because large matrices can take a long time to compute. It is not easy to predict the correct thresholds programatically, so manual adjustment is often necessary. The Greyramp Tool allows the user to interactively change the upper and lower cutoffs with simple sliders on a greyramp image. A contrast sider is also available to move both cutoffs simultaneously. A simpler, minimised version of the greyramp tool is available which allows quick access to the contrast slider while maximising the screen space available for the plot.

The dot-plot can be navigated with keyboard shortcuts or the mouse. A crosshair displays the currently-selected position coordinates. The coordinates can easily be copied to the clipboard. The sequence data at the crosshair position can be inspected using the alignment tool. This tool places the crosshair position at the centre of its display to give a good view of the sequence on either side. In nucleotide mode it displays both strands of the reference sequence, and in protein mode it displays the three-frame translation. Bases are highlighted according to how well they match (exact, conserved or mismatch) so it is easy to see whether the current position is on the alignment.

The alignment tool scrolls in unison with the crosshair, and can be scrolled using keyboard shortcuts. Each sequence can be scrolled independently, or they can be scrolled in unison in order to walk along the alignment. Scrolling can be per-nucleotide or per-peptide, allowing very fine control of the crosshair/sequence position. Sections of the sequence can be selected in the alignment tool and the selected sequence and its coordinates can be copied to the clipboard.

Features such as gene-models and high-scoring pairs (HSPs) can be loaded from a GFF file or piped from a calling program such as Blixem. HSPs can be superimposed onto the dot-plot, and gene models are displayed along the axis of the relevant sequence, so they can be easily compared with the alignment. Internal repeats can be analysed by running Dotter on a sequence versus itself. Overlaps between several sequences can be found by concatenating the sequences and running Dotter on the concatenated sequence versus itself. In the case where you have multiple sequences on each axis, a grid can be displayed showing which section is from which sequence.

Dotter offers the option to save the plot to file, which is useful for large plots that take a long time to compute. Dotter can also be run in batch mode, where the matrix is computed in the background and saved for viewing later. Recreating a plot from a saved matrix is very fast, and has the advantage that it can still be dynamically adjusted and interacted with.

#### Belvu

Belvu is a multiple sequence alignment (MSA) viewer and editor used to create high-quality reference alignments. Belvu can load and save alignments in Stockholm, MSF, Selex and Fasta file formats.

**Fig. 3:**
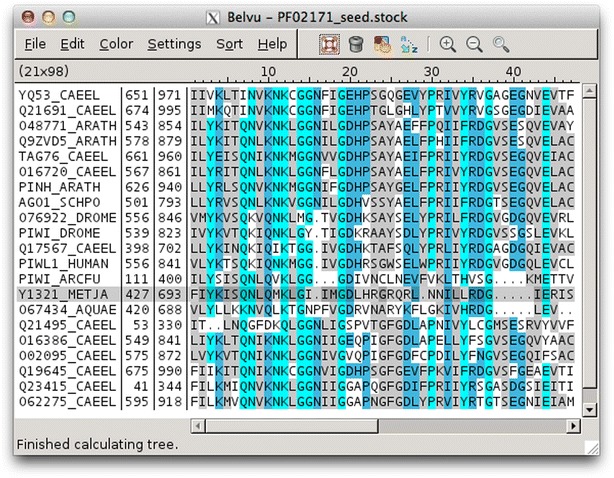
Belvu colour-by-conservation mode. Multiple sequence alignment in Belvu with residues coloured by average similarity by BLOSUM62. *Cyan* indicates conservation of 3.0 or greater, *blue* 1.5, and *grey* 0.5. Colours and thresholds are user-configurable

Opening an alignment brings up Belvu’s graphical alignment window. By default, residues are coloured by conservation (Fig. 3). Conservation can be calculated by average similarity by BLOSUM62, by percent identity (either including or ignoring gaps), or by a combination of percent identity and BLOSUM62. Residues are then coloured according to user-configurable conservation thresholds.

**Fig. 4:**
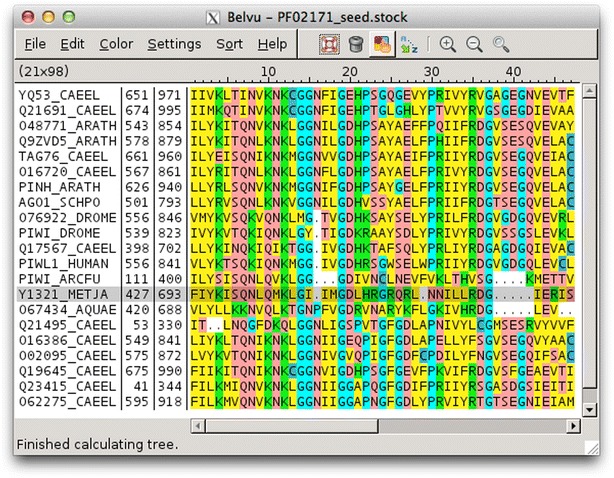
Belvu in colour-by-residue mode. Multiple sequence alignment in Belvu with residues coloured by residue type. Colours are user-configureable and thresholds can be specified to only colour residues with a given percent identity or higher

Residues can also be coloured by residue type (Fig. 4). There are several built-in modes for colouring residues by type, and colours can be edited to create custom colour schemes that can be saved to and loaded from file. Colouring can be applied to all residues, or to only those residues with a percent identity above a specified threshold. Again, gaps can be included or ignored in the identity calculation.

**Fig. 5:**
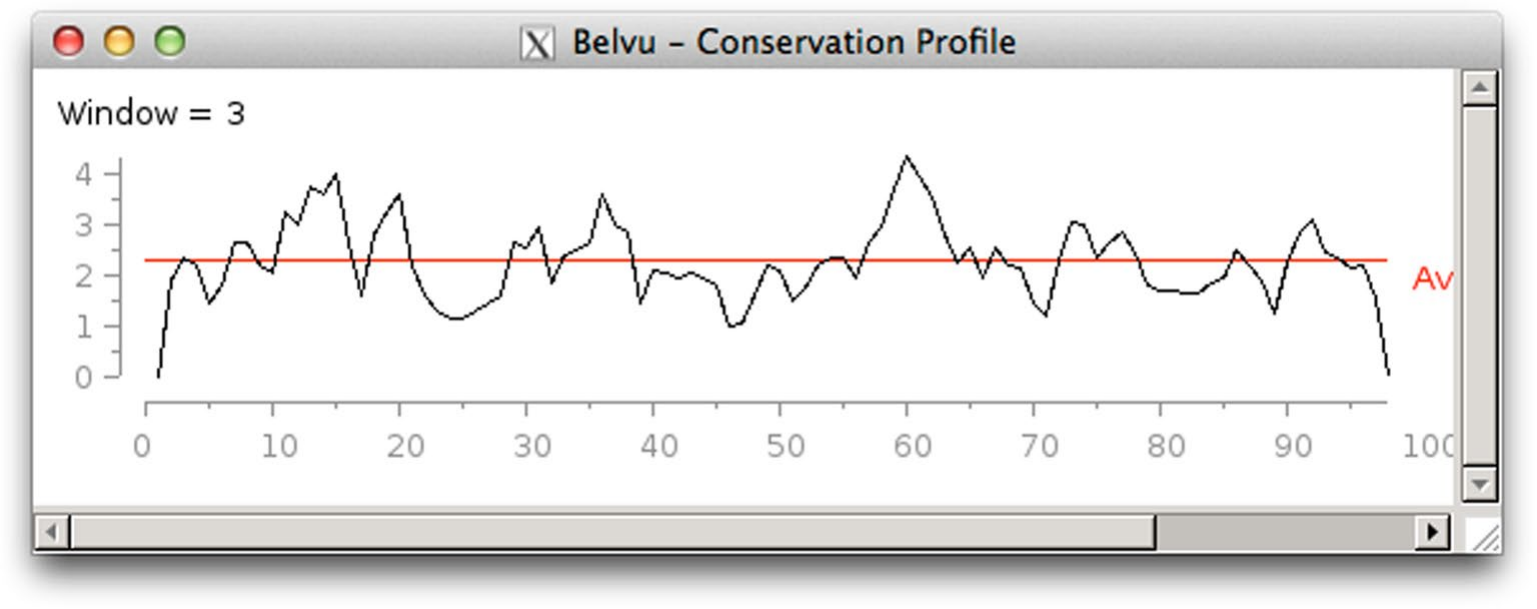
Belvu conservation profile. The maximum conservation for each column is plotted against the column number. The *red line* shows the average conservation, and a sliding-window of three has been applied for smoothing

Belvu can display a conservation profile for the alignment (Fig. 5), which shows the maximum conservation for each column. The plot can be smoothed by applying a sliding window of user-configurable size.

Belvu has basic alignment editing capabilities, whereby sequences or columns can be removed. Sequences can be removed by manual selection, or by automatically removing those that match certain criteria such as the percentage of gaps they contain, their score, or whether they are a redundant or partial sequence. Similarly, columns can be removed by criteria such as the percentage of gaps or the maximum conservation in the column.

**Fig. 6:**
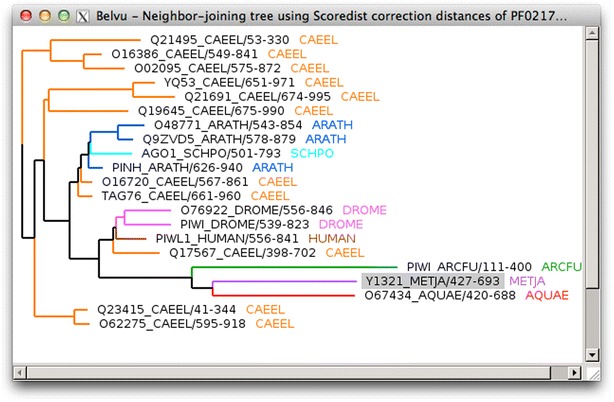
Belvu phylogenetic tree. Neighbour-joining tree using Scoredist correction

Belvu can construct a distance-based phylogenetic tree from a multiple alignment, using either neighbour-joining or UPGMA tree construction methods (Fig. 6). Distance correction can be applied using Scoredist [4], Storm and Sonnhammer, Kimura or Jukes–Cantor methods. Tree nodes can be swapped, and the tree can be re-rooted around a particular node. Trees can be saved in New Hampshire format. The distance matrix can also be exported, and the tree can be reconstructed from a saved distance matrix. Belvu can also perform bootstrap analysis.

Belvu has extensive command-line capabilities, which includes most of its non-interactive functionality: file format conversion; distance-matrix construction; tree generation using a variety of methods; distance-matrix construction; tree reconstruction from a distance matrix; bootstrap analysis; and automated editing operations based on caller-specified criteria such as conservation and percentage of gaps. Multiple command-line operations can be used in conjunction with one another, making powerful, automated processing possible. This makes Belvu ideal for use in software pipelines. Table 1 shows some examples of the type of operation that can be performed; many other combinations are also possible.

**Table 1:** Belvu command-line examples. Examples of the type of processing that can be performed in a single command-line call to Belvu

Input	Processing	Output
Alignment	Export to a different format	Alignment
Alignment	Remove sequences >80 % identical and columns with conservation <0.9	Alignment
Alignment	Construct neighbour-joining tree using Kimura distance correction	Distance matrix
Distance matrix	Reconstruct tree	Tree
Alignment	Apply bootstrap analysis	Bootstrap trees

### Results and discussion

Blixem and Dotter are used extensively by the HAVANA group at the Wellcome Trust Sanger Institute and are essential to the manual annotation process. Examples of work published by the HAVANA group that has involved the use of Blixem and Dotter includes [5–10]. Belvu is used in the curation of high-quality “seed” alignments for the Pfam database [11].

This section provides some examples of the type of work that each tool has been used for. We also compare the tools with other, similar tools that are available. This comparison is aimed at highlighting what makes the SeqTools programs unique and is not intended to be a comprehensive review of all features.

#### Blixem

Blixem is the “workhorse” for manual gene annotation in the HAVANA group and other collaborating institutes and is in continuous use. It represents the annotator’s first point of contact at nucleotide-level for all sequence alignment data, including ESTs, mRNAs and proteins. Annotators use Blixem to check the quality of an alignment (including finding missing or misaligned sequence), to identify splice sites (using this information to construct gene models and confirm alternative splicing events), to identify polyA sites and signals, to investigate the functional effects of polymorphisms (including reference sequence disabling variants in polymorphic pseudogenes), to investigate potential genome sequence errors, to identify deleterious mutations in pseudogenes, and in rarer cases to identify selenocysteine residues and RNA editing events.

As far as the authors are aware, there are no other tools available that are quite like Blixem. The tools that come closest are BAM viewers such as the UGENE Assembly Browser. This displays multiple reads aligned under the reference/consensus sequence with differences highlighted in a similar manner to Blixem. However, it is very much aimed at BAM data rather than general alignment data, and as it only reads BAM files it cannot display other feature types (although support for other file formats may be planned).

Artemis/BamView offers a similar stacked view for BAM reads, but again this is aimed solely at BAM. Artemis can also show alignments from a GFF file but does not combine them into the same stacked display—instead, they are shown next to the sequence display, but overlapped so that you have to select from a list to see a specific feature highlighted.

Gap5 is another tool that superficially looks similar to Blixem because it stacks multiple matches against a reference sequence. However, this is an assembly editor and is a much larger, complicated system than Blixem. It requires data to be converted to a gap5 database so cannot be used to simply view the contents of a GFF file.

#### Dotter

Dotter is essential to the HAVANA manual annotation process and is used for two main purposes:

Firstly, to identify sequence that is not represented (or is misrepresented) in the EST2genome or BLAST alignments produced by the analysis pipeline and displayed in Blixem. Identification of missing sequences using Dotter frequently allows annotation of models with alternative 5′ UTR and internal exons that are short in length, repeatmasked in the reference genome sequence, or contain transcript sequence errors/polymorphism that hinders their automated alignment. As the HAVANA group only annotate gene models to the extent they are supported by aligned evidence (i.e. they do not extend models by creating a tiling path of evidence or “borrowing” exons from other full-length models at the same locus), Dotter is useful to support the extension of gene models supported by evidence from paralogous and orthologous loci and locus-specific ESTs of inconsistent sequence quality. Secondly, to facilitate the rapid comparison of annotated gene models with transcriptional and protein evidence that putatively support them as breaks in alignment are more clearly identifiable than in a pairwise sequence alignment e.g. Clustal.

There are several dot-matrix alignment programs available such as Gepard, SEROLIS, DNAdot, GenomeDiff, Dotlet and UGENE’s Dot Plot viewer, but most of them lack tools similar to Dotter’s Greyramp and Alignment tool. The only two programs we found that offer a Greyramp tool are Gepard and SEROLIS. SEROLIS only supports very short alignments (4k max) and even so it is very slow (in one example we ran it took 8 s to open on a 112k reference sequence that it truncated to 4k, whereas Dotter opened using the full 112k sequence in around 3 s). Its greyramp tool is also very slow (taking around the same time again that it took to start up for each adjustment).

Gepard on the other hand has a good greyramp tool with a similar level of control as Dotter. Gepard also has a reasonable alignment tool and crosshair control—it was the only other tool we found that has keyboard shortcuts to walk along the alignment. Gepard also has the advantage over Dotter is that it uses an alternative algorithm based on suffix arrays for large sequences, which means it can calculate the alignment very quickly (they quote <2 min for an *Escherichia coli* self plot, which would take Dotter several days). However, for small to medium sized plots Dotter still has some advantages—its alignment tool is more sophisticated (Gepard does not highlight matching bases or allow sequence selection), and Dotter can display gene models and HSPs loaded from a GFF file.

Dotter and Gepard are the only tools we are aware of that save plots which can be fully interacted with when re-loaded. Gepard does this by saving the suffix array, rather than the dot-plot, so there may be a delay in opening it as it calculates the plot, although this is minimal compared to the time it takes to calculate the suffix array. Dotter’s approach is to save the dot-matrix to a binary format file. This takes longer to calculate than Gepard’s suffix arrays, but it opens instantly. Both tools have a command-line mode that allows the plot to be calculated and saved as a background job.

#### Belvu

Belvu is used in the manual curation of high-quality “seed” alignments for the Pfam database [11]. Annotators might start with an alignment from MUSCLE or MAFFT, for example, and use Belvu to trim the ends of the alignment to the best conservation, and remove gappy and partial sequences. They use Belvu to analyse conservation patterns, sorting alphabetically to see readily repeated domains on a sequence, or sorting by tree order to see simple evolutionary relationships. They can also sort by similarity to a specific sequence, which is useful when trying to spot false positives. Redundant sequences are removed in order to see the variation across the whole. Once of a high enough quality, the seed alignment is then used to automatically generate a “full” alignment, which contains all detectable protein sequences belonging to the family.

There are many MSA viewers, editors and phylogenetic tools available, offering a wide variety of features. To name but a few: Jalview2, ClustalX, UGENE, AliView, BaseByBase, CINEMA5, BioEdit, CLC Viewer, SeaView, Geneious, Jevtrace2, MVIEW, PFAAT, Sequlator, STRAP, Squint. Briefly, the features of Belvu that stand out as being generally lacking or less comprehensive in other tools are: its customisable colouring of residues by similarity and percent identity; its automated editing functions; and its extensive command-line mode.

Many other programs have per-residue colour schemes, many with schemes that can be edited and saved, but few have colouring by similarity and percent identity. Those that do are Jalview, BaseByBase, UGENE and Jevtrace. Of these, only BaseByBase, UGENE and Jevtrace have Belvu-style low/medium/high thresholds which display residues in a single colour per threshold. UGENE, however, can only do this for percent identity (not similarity), and neither UGENE nor BaseByBase has configurable thresholds like Belvu and Jevtrace. Other programs tend to just use the per-residue colours which are simply turned on or off based on a single threshold, which does not give such a clear view of the conservation patterns.

While Belvu’s editing capabilities are not intended to be comprehensive, it still has more functionality than many of the tools available, particularly regarding automatic removal of sequences and columns. While many tools allow manual deletion of sequences/columns and manual removal of gaps etc., few are able to automatically delete columns and sequences based on customisable criteria. Some of the operations that are available in other tools are: removal of all-gap columns (ClustalX, Jalview, AliView, UGENE, BaseByBase, SeaView, PFAAT, STRAP); removal of all gaps (ClustalX, UGENE, Jalview, STRAP); removal of redundant sequences (Jalview, PFAAT, SeaView); removal of a column by user-specified percentage of gaps (UGENE, PFAAT); and filtering of sequences by percent identity (MView, Jevtrace). Functionality that we did not find in any other programs includes: removal of sequences by percentage gaps; removal of partial sequences (those starting or ending with gaps); and removal of columns by conservation (with user-specified upper/lower cutoffs).

Command-line operation is not available in most of the tools we tested. SeqView has a small number of command line options such as tree and distance-matrix construction, and conversion to other formats. UGENE offers many command-line operations, such as conversion to other formats and finding ORFs and repeats. However, none of these tools offer editing commands on the command line, so Belvu is the only tool that we are aware of that can do this. In conjunction with its many automated editing commands and customisable criteria, this makes Belvu the most powerful command-line editing tool available.

### Conclusions

Blixem is a unique tool that displays multiple match sequences aligned against a single reference sequence. This allows annotators to compare evidence from a variety of sources in a very detailed nucleotide-level view. Support for new data types such as short-reads means that the annotator can see all relevant information in one place in the context of selected features.

Dotter gives a graphical view of a particular pairwise alignment. Its dynamically-adjustable contrast allows annotators to quickly tweak the display to get the best view of the results, and the alignment tool offers a detailed view of the aligned sequence. The alignment can easily be compared to annotated gene models and HSPs. Dotter is extremely useful for identifying alignments that cannot be found using automated methods, and for validating putative evidence.

Belvu is one of many programs available for viewing and editing multiple alignments and generating phylogenetic trees. Belvu’s advantages are its extensive command-line mode, its automatic editing operations, and its highly configurable colour schemes, including similarity-based schemes which give a very clear view of conservation patterns. Belvu is also the only tool to implement the Scoredist algorithm [4].

## Availability and requirements

- Project name: SeqTools;
- Project home page: http://www.sanger.ac.uk/resources/software/seqtools/;
- Operating system(s): Linux, Mac OS X, FreeBSD, Cygwin;
- Programming language: C;
- Other requirements: GTK+ 2.12 or higher;
- Licence: GNU GPL version 3;
- Any restrictions to use by non-academics: none.

The source code for the production version of SeqTools at the time of writing is available as Additional file 1.

## Additional file

10.1186/s13104-016-1847-2 A tarball of the current production release of the SeqTools source code at the time of writing.

## Abbreviations

MSA: multiple sequence alignment
SNP: single nucleotide polymorphism
GFF: generic feature format
NGS: next-generation sequencing
HSP: high-scoring pair
