# Supplementary material for: SeqTools: visual tools for manual analysis of sequence alignments
Source: BMC Res Notes. 2016 Jan 22;9:39. doi: 10.1186/s13104-016-1847-3 (PMC4724122; doi:10.1186/s13104-016-1847-3)
Supplement: Supplementary file 1 — 10.1186/s13104-016-1847-2 A tarball of the current production release of the SeqTools source code at the time of writing. [file 13104_2016_1847_MOESM1_ESM.gz › seqtools-4.32.1/doc/Design_notes/architecture.html]

SeqTools - Architecture


# Architecture

The SeqTools package consists of the following modules:  

blixemApp: blixem and blixemh application code  
dotterApp: dotter application code  
belvuApp: belvu application code  
seqtoolsUtils: generic utilities  
libpfetch: utilities for fetching over http (only required for blixemh: requires libcurl)
