# Supplementary material for: SeqTools: visual tools for manual analysis of sequence alignments
Source: BMC Res Notes. 2016 Jan 22;9:39. doi: 10.1186/s13104-016-1847-3 (PMC4724122; doi:10.1186/s13104-016-1847-3)
Supplement: Supplementary file 1 — 10.1186/s13104-016-1847-2 A tarball of the current production release of the SeqTools source code at the time of writing. [file 13104_2016_1847_MOESM1_ESM.gz › seqtools-4.32.1/doc/Design_notes/build.html]

SeqTools - Build System


# Build System

This page describes how to compile the SeqTools package using the Autotools scripts included with the source code.

## Autotools

The SeqTools package is configured and built using the GNU Build System (aka Autotools). The relevant configuration files are listed below:

- src/version.m4: This specifies the version number for the build. It is included by configure.ac, which sets the version number in the PACKAGE\_VERSION variable in the config.h file, which is included by the code.- src/configure.ac: This file is processed by autoconf to produce the configure file.- src/Makefile.am: This file is processed by automake to produce the Makefile.in file.- src/AUTHORS, src/COPYING, src/README, src/NEWS, src/ChangeLog: These files are for information.- subdirectory Makefile.am files: The subdirectories blixemApp, dotterApp, seqtoolsUtils and libpfetch all contain a Makefile.am file, which is processed by automake to create a Makefile.in file in that directory.

The build process is as follows:

- Run '`autoreconf -i`' in the `src` directory. This runs autoheader, aclocal, autoconf and automake and creates the configure script (and some other required files) in the src directory.- `cd` to the directory where the code is to be built (this can be src or any other directory, e.g. src/build) and run the configure script from there. This will create a makefile in the build directory (and subdirectories for blixemApp, dotterApp, seqtoolsUtils and libpfetch, which all contain a makefile for building that module). It will also create the config.h file that will be included by the code, and a few other required files.- Run `make` in the build directory to build everything (or in a subdirectory to build just that module).

## Modules

The source code includes the following modules. Each module has its own subdirectory with a makefile that produces a `.a` convenience library that is statically linked by the executables that require that code. The executables are built by the main `Makefile` in the `src` directory. The blixem and blixemh executables both use the same source code (the blixemApp code) but blixemh is compiled using the `PFETCH_HTML` flag, which conditionally compiles some additional code.

- blixemApp: Blixem application code.- dotterApp: Dotter application code.- seqtoolsUtils: Generic utility functions. Required by dotter, blixem and blixemh.- libpfetch: Utilities for fetching sequences over http. Required by blixemh.

## Dependencies

- All of the executables in the SeqTools package require the GLib and GTK+ libraries. These are built using pkg-config. configure.ac specifies which version of the libraries are required.- blixemh uses the libpfetch library, which requires the libcurl library.
