# Supplementary material for: SeqTools: visual tools for manual analysis of sequence alignments
Source: BMC Res Notes. 2016 Jan 22;9:39. doi: 10.1186/s13104-016-1847-3 (PMC4724122; doi:10.1186/s13104-016-1847-3)
Supplement: Supplementary file 1 — 10.1186/s13104-016-1847-2 A tarball of the current production release of the SeqTools source code at the time of writing. [file 13104_2016_1847_MOESM1_ESM.gz › seqtools-4.32.1/doc/Design_notes/modules/belvuApp.html]

SeqTools - belvuApp


# belvuApp

The belvuApp module contains the code for the Belvu application.

## `main()` function

The Belvu `main()` function lives in `belvuMain.c`. The `main()` function performs the following jobs.

- read in the command-line arguments that were passed to Belvu;- print usage information if incorrect arguments, or the help argument, were passed;- parse the input alignment file (or the input distance matrix, if argument `-T R` was given;- convert swissprot name suffixes to organisms;- set the default colours for organisms;- read in the scores file, if given;- sort alignments by the default sort order (or the given sort order, if passed as an argument);- read in match segments from a match file, if given, or look for match footers in the input alignment file, if given;- set the initial residue colours;- print the conservation table and exit, if requested;- read in residue and/or markup colours from a colour file, if given;- make the alignment non-redundant, if requested;- remove partial sequences, if requested;- remove gappy columns, if requested;- remove gappy sequences, if requested;- save the alignment and exit, if requested;- create bootstrap trees and exit, if requested;- output model probabilities and exit, if requested;- print subfamilies and exit, if requested;- show the annotation window, if requested;- display bootstrap trees, if requested;- display the main window (and/or the tree, if requested); and- enter the main GTK loop to await user interaction.

## The Belvu window

`belvuWindow.h/.c`

We use GTK+ widgets to create the Belvu graphical components. The main Belvu window is created by the `createBelvuWindow()` function in `belvuWindow.c`.

A Belvu window comprises the following components:

- Menubar- Toolbar- Alignment section- Statusbar

### The alignment section

`belvuAlignment.h/.c`

This is the main portion of the Belvu window and shows the actual alignment. There are two main modes for this window: normal and 'wrapped'. The normal mode is currently only used for the alignment in the main window. The wrapped mode is used whenever we create a wrapped-alignment window for printing; we can have as many wrapped-alignment windows as we like.

In normal mode, there are two drawing areas: one for the static columns such as name, start and end; these do not scroll horizontally (although they do scroll vertically). The other drawing area displays the sequence data, and scrolls both horizontally and vertically. In wrapped mode, only the sequence area is displayed (although sequence names are still drawn, they are all drawn inside the one widget and the whole thing scrolls together). Since the sequence area always exists, this widget controls the vertical scrolling; if the columns area also exists, it is scrolled in sync with the sequence area.

In normal mode, the user will be interacting with the widget a lot, so update speed is essential; therefore, only the currently-visible characters are drawn. Wrapped mode, however, is designed for printing, so the whole area must be drawn, which can be slow. There is also a limitation on the size of this window because we use a GdkPixmap to do the drawing, and we get a crash if we request a GdkPixmap that is too large (this appears to be undocumented, so we have had to guess a reasonable upper limit on the size). The size limit is unlikley to be a problem though seeing as this window is intended to be printed, and the maximum window size is already much larger than anyone would reasonably want to print.

## The Tree window

`belvuTree.h/.c`

This window displays the tree. There is only ever one tree (stored in the main context), and it must be calculated before the tree window can be shown. The underlying tree must be recalculated whenever it is invalidated by making changes such as deleting sequences or columns; we don't recalculate the tree automatically because it can take a long time and the user may have further edits to make. The tree window must therefore be able to handle an invalid (null) tree until the user requests it to be recalculated. There is only ever one tree window; if the user re-opens it, we re-display the original window.

## The Conservation Plot

`belvuConstPlot.h/.c`

This window shows the plot of the conservation profile. There is only ever one conservation plot window.

## The Annotation Window

This displays a list of all the annotation lines. There is only ever one annotation window. It is currently only available if requested on the command-line, and cannot be reopened if closed; it would make more sense to be able to open it from within the program.
