# Supplementary material for: SeqTools: visual tools for manual analysis of sequence alignments
Source: BMC Res Notes. 2016 Jan 22;9:39. doi: 10.1186/s13104-016-1847-3 (PMC4724122; doi:10.1186/s13104-016-1847-3)
Supplement: Supplementary file 1 — 10.1186/s13104-016-1847-2 A tarball of the current production release of the SeqTools source code at the time of writing. [file 13104_2016_1847_MOESM1_ESM.gz › seqtools-4.32.1/doc/Design_notes/modules/blixemApp.html]

SeqTools - blixemApp


# blixemApp

The blixemApp module contains the code for the Blixem application. There are two different Blixem executables: blixem and blixemh. They are essentially the same, but blixemh has some additional functionality that allows sequences to be fetched over http. This introduces a dependency on the libcurl library, so we also offer the standard blixem executable which can be compiled on systems where libcurl is not available. The same source files are used for both executables, with the libpfetch dependencies being conditionally compiled via use of the PFETCH\_HTML flag. The design notes here refer to both blixem and blixemh unless otherwise specified.

## `main()` function

The Blixem `main()` function lives in `blxmain.c`. The `main()` function performs the following jobs:

- read in the command-line arguments that were passed to Blixem;- parse the input files;- create the Blixem window; and- enter the main GTK loop to await user interaction.

## The Blixem window

`blxwindow.h/.c`

We use GTK+ widgets to create the Blixem graphical components. The main Blixem window is created by the `blxview()` function in `blxview.c`. `blxview()` also does any initialisation required, e.g. fetching any sequence data that was not provided in the input files.

The Blixem window has two main sections, the 'Big Picture' and the 'Detail View', which are separated by a splitter bar (by placing the detail-view and big-picture widgets in a GtkPaned). There is also a scrollbar at the bottom which controls the range of coordinates that is currently displayed in the Detail View. The Big Picture also uses this range because it highlights the current detail-view range and centres itself on it.

### A note on properties

We need to be able to set various properties for the main blixem window (as well as other widgets). We can set a property on a widet using the `g_object_set_data()` function. This requires passing the property name as a string, so we define 'set' and 'get' functions for any properties that we wish to set, so that the string need only be specified in these two functions, reducing the scope for error.

Rather than creating a 'get' and 'set' function for each property we wish to set, we place all properties in a struct and set the struct as the property on the widget. This means that we can access all properties with one call to the `g_object_get_data` function (via our 'get' function), which is advantageous because this can be slow if called many times. For the main window, our properties struct will be called something like `BlxWindowProperties`, will be created by `blxWindowCreateProperties()` and accessed via `blxWindowGetProperties()`.

If we just wish to access one property in the properties struct, it is convenient to have an access function that gets the properties struct and returns that particular property. However, one should avoid using these convenience functions if multiple properties are required - it is more efficient to get the properties struct once and access the individual properties directly from there.

In addition to these properties structs that are set on widgets, there is also a Blixem-wide properties struct called the 'Blixem context'. This is where to store properties that do not pertain to any particular widget.

### Big Picture

`bigpicture.h/.c`

The "big picture" section displays an overview of the entire portion of reference sequence that was passed to Blixem. The reference sequence coordinates are shown along the top. Alignments are shown as lines and are placed in a scaled grid (one for each strand) against their %ID. Exons and introns are drawn in standard notation in the transcript section.

The Big Picture contains the following main widgets, all inside a vbox. The vbox is placed inside a scrolled window so that all of the contents scroll together if they get too big for their pane. Any of these main widgets can be hidden to save space.

- Forward-strand grid: the grid showing alignments on the forward strand.- Forward-strand exons: a drawing area showing exons and introns on the forward strand.- Reverse-strand exons: a drawing area showing exons and introns on the forward strand.- Reverse-strand grid: the grid showing alignments on the forward strand.

The vbox also contains a header widget, which displays labels showing the reference sequence coordinates. This was designed as a separate widget (rather than being included as part of a grid) so that it is unaffected if a grid is hidden or the order is changed. The labels are spaced at the same intervals as the vertical lines on the grids. Some work is put into dividing the big-picture range so that we have nicely-spaced lines with nice round numbers as the headers. This is not trivial because we may wish to round to, say, the nearest 5 coordinates if we have a very small range or to the nearest 10000 for a large range. We do this by maintaining a list of 'nice' values that are acceptable values to round to.

There are zoom buttons on the big picture that allow the user to zoom in or out. "Zoom whole" zooms out so that we are viewing the whole section of reference sequence that was passed to Blixem. If we are zoomed in at all then we are obviously not viewing the full reference sequence range - the range that is displayed in the big picture is centred on the detail-view range, and scrolls when the detail-view range scrolls.

The current detail-view range is highlighted as a shaded area on the big picture. (This highlighting is done by having each grid and exon-view draw a shaded background spanning the relevant range of coordinates before drawing its real contents on top. Crude as this is, the effect is pretty good.) The user can select a different detail-view range by middle-clicking/middle-dragging in the big picture. This will show a preview box (similar to the original shaded area) of where the new detail-view range will go. Both views will scroll to the new range when the mouse button is released.

#### Re-ordering and hiding widgets

The default order is that the forward-strand grid and exon-view are shown at the top with the reverse-strand widgets below. However, if the user toggles the display (so that the reverse strand becomes the active strand), the reverse-strand grid and exon-view are then shown at the top. To change the order of the widgets, they are simply removed from their parent and re-added in the correct order. To hide a widget we essentially use the `gtk_widget_hide()` function, but we also set a custom property to indicate that the widget has been hidden by the user, because any call to `gtk_widget_show()` (e.g. when we change the order of the grids) will otherwise un-do the hide.

#### Big-Picture Grid

`bigpicturegrid.h/.c`

Each grid on the Big Picture is a GtkLayout. The grid's properties-struct will define which strand of the reference sequence the grid relates to. Vertical lines are drawn at regular intervals corresponding to the labeled reference sequence coordinates in the big-picture-header. Horizontal lines are drawn at regular intervals to indicate the %ID of alignments. The default of %ID values shown on the grid is *n*% to 100%, where *n* is the lowest %ID of any alignment in the current Blixem session. (This can save valuable drawing space, because we can draw a smaller grid if all alignments have a high %ID.)

Alignments are drawn on the grid as a horizontal line spanning the range of reference sequence coordinates where the alignment lies. They are drawn at a y-coordinate corresponding to their %ID. In theory the line should have zero height, but obviously we give it some height so that it can be seen and clicked on. Clicking on an alignment will select it in both the big-picture and in the detail-view.

#### Exon-View

`exonview.h/.c`

The 'Exon View' shows all exons and introns for the current big-picture range. (Perhaps 'Transcript View' would be a better name.) An exon can be split into two regions: CDS (coding) and UTR (non-coding/untranslated), or it may be entirely CDS or entirely UTR. We represent this by having a 'parent' exon object with one or two child objects for the CDS and/or UTR regions of that exon. This makes drawing the exons very easy because we just spin through all of the CDS/UTR objects and draw them at their given coordinates. However, we may not be supplied all of these objects in the input file (in fact the GFF3 specification recommends that one supply only exons and CDSs, since the rest can be implied), so we have to do some work on initialisation to fill in any missing objects. This is done in the `finaliseBlxSequences()` call from `blxview()`.

Introns are also stored as objects that can be drawn directly. Again, they may not be supplied in the input file, so we must work them out. We work out whether an intron is CDS or UTR according to whether its adjacent exons are CDS or UTR.

There are two exon-views: one for each strand. By default, all exons and introns for a strand are drawn on the same line, overlapping. They can be 'bumped' so that they display on separate lines instead.

### Detail View

`detailview.h/.c`

The 'Detail View' shows the alignment sequences in detail. It has a different layout depending on whether we are in nucleotide or protein mode:

- nucleotide mode: two panes, one for the forward strand and one for the reverse strand, with the active strand shown at the top.- protein mode: three panes, one for each reading frame. Shows only the active strand.

The 'active strand' is the forward strand by default. The user can switch to the reverse strand (or back again) using the 'toggle' option.

Each pane in the detail view therefore shows one reading frame of one strand of the reference sequence. The panes are separated by splitter bars. (Note that we need three panes in protein mode but a GtkPaned widget only provides two panes, so we need to have a second, nested GtkPaned inside the first GtkPaned widget in this case.) The detail-view also has a toolbar and a header widget. The header widget shows the labels for the columns in the pane. It is a separate widget because we only need to show these labels once, rather than showing them for each pane.

#### Detail-view pane

`detailviewtree.h/.c`

Each pane in the detail-view shows a list of alignments for a particular reading-frame and strand of the reference sequence. A list is implemented as a GtkTreeView, so the code often refers to it as a detail-view-tree.

An alignment list has several columns, such as the sequence name, %ID, start and end coordinates etc. The main column of interest however is the 'sequence column', which shows the actual sequence data. The list has a header, which shows the current portion of the reference sequence above the 'sequence column', so that the match sequences are shown lined up with the reference sequence. Things like SNPs and polyA signals can be highlighted in the reference sequence header. The header also shows the reference sequence name and the start and end coordinates of the currently-displayed section of reference sequence. (Note that this is a different use of the start and end column to that used by the alignment list, which uses these columns to show the start and end coordinates of the alignment on the match sequence - i.e. absolute coords, not the currently-displayed range.)

One row in the tree represents one match sequence or exon. Only matches/exons that lie within the current detail-view display range are visible in the tree. The display range is controlled by the scrollbar at the bottom of the main window. All detail-view trees share the same scrollbar/display-range so that they all show the same section of reference sequence. The cell contents of the tree are drawn by a custom cell renderer.

Middle-clicking/middle-dragging in the detail-view tree highlights the moused-over coordinate, and feeds back info about the currently selected coordinate in the feedback box on the toolbar. Left-clicking selects a row, and feeds back info about the selected sequence in the feedback box. Multiple rows can be selected and operations can be performed on the selected sequence(s), e.g. creating a group from them or running Dotter.

Note: The aim in using GtkTreeView was to take advantage of all of the functionality that comes with it: filtering, sorting, row selections, column headers, column resizing etc. etc. However, as more customisations have been added a lot of this functionality has had to be implemented manually, so perhaps in hindsight it would have been better to use a GtkLayout and do all of the drawing manually (which might give us better performance). If we ever need to change to a real tree with child rows, though (e.g. squash-matches could be implemented this way), having GtkTreeView may be advantageous. The main downside of using GtkTreeView is that when the user middle-clicks/drags, to highlight the current column the whole tree has to be redrawn. Ideally we would not have to redraw the tree but would XOR the highlight box over the top of it.

#### Detail-view columns

The `BlxColumnId` enum defines a list of all columns that should exist in the detail-view-panes. The columns in the GtkTreeViews are created from this information during initialisation. This makes it relatively easy to add new columns - see the note against `BlxColumnId` in `blixem_.h` for details.

#### Toolbar

The toolbar is part of the detail view for historic reasons but actually contains some functions relating to the whole of Blixem. It contains a drop-down box for sorting the detail-vew, buttons for Settings and Help, buttons for navigation and zooming in the detail-view, a feedback box containing info about the currently selected match/coordinate and a feedback area containing info about the currently moused-over match.

### Sequences, selections and groups

A Group is a struct that stores a list of (match) sequences, along with any properties that are relevant (e.g. highlight color, visibility etc.). A Selection is essentially just a list of sequences too, so many functions are designed to operate on a list of sequences so that they work with either a Group or a Selection. The Groups functionality is very basic at the moment, offering just a simple mechanism to highlight or hide a set of sequences, but it could be extended to give much more powerful filtering and sorting options.

## Calling Dotter

Blixem can call Dotter on the currently selected sequence. (Currently this is limited to a single sequence at a time, but the aim is to allow multiple Dotters to be started simultaneously from a multiple selection). Dotter is spawned as a new process. Blixem keeps track of all of the processes it has started via the spawnedProcesses member of the blixem context, and kills them when the main Blixem window is destroyed.

### Call Dotter on Self

This option allows the user to call Dotter on the reference sequence versus itself. The aim of this option is to be able to analyse internal repeats.

### Dotter parameters

When the user selects the 'Dotter' option they get presented with the Dotter dialog, which allows them to edit parameters before calling Dotter. The default parameters are for Auto coords, and for a Zoom factor of 0 (which means 'none', i.e. Dotter will calculate a sensible default zoom). Default parameters should be ok in most cases, so all the user needs to do is hit Execute. They can also specify parameters by entering coords manually or by clicking one of the buttons to use the current big-picture range or the full reference-sequence range. They can save the parameters if they don't want to execute Dotter immediately. (Note that parameters are also saved when the user hits the Execute button.) The auto/manual option will be remembered when parameters are saved, too, and the saved option will be the default the next time the dialog is opened.

### Auto coords

The 'Smart' or 'Auto' coords option means that Blixem will attempt to calculate a sensible range of coords to call Dotter with, based on the sequence that is currently selected. The auto coords need to be calculated when the Dotter dialog is opened, so that they can be displayed to the user before they execute Dotter. They need to be recalculated if the user selects a different sequence, selects the 'Self' option, or switches back to Auto coords after selecting the Manual coords option. For 'Self' mode, the auto range used is centred on the current detail-view range, and the length of the range is hard-coded (currently 2000 bases).

### Fetching sequence data

In the old seqbl file format, it was possible to get incomplete sequence data passed in (i.e. not the full length of the match sequence). Therefore, by default Blixem will try to fetch the sequence from the pfetch server (only available to users within the Sanger Institute) in order to get the full sequence data if possible. If this fails, it will use the sequence data that is stored in Blixem. The sequence data is fetched when the Dotter dialog is first opened because it is used to calculate the smart dotter range. It needs to be re-fetched if the user selects a different sequence.
