# Supplementary material for: SeqTools: visual tools for manual analysis of sequence alignments
Source: BMC Res Notes. 2016 Jan 22;9:39. doi: 10.1186/s13104-016-1847-3 (PMC4724122; doi:10.1186/s13104-016-1847-3)
Supplement: Supplementary file 1 — 10.1186/s13104-016-1847-2 A tarball of the current production release of the SeqTools source code at the time of writing. [file 13104_2016_1847_MOESM1_ESM.gz › seqtools-4.32.1/doc/Design_notes/modules/dotterApp.html]

SeqTools - dotterApp


# dotterApp

The dotterApp module contains the code for the Dotter application. Dotter can be called standalone from the command line, but is often called from Blixem.

## `main()` function

The Dotter `main()` function lives in `dotterMain.c`. The `main()` function performs the following jobs:

- read in the command-line arguments that were passed to Dotter, OR read in the streamed-in arguments if called from within SeqTools;- parse the input files;- create the Dotter window; and- enter the main GTK loop to await user interaction.

## The Dotter window

`dotter.h/.c`

We use GTK+ widgets to create the Dotter graphical components. The main Dotter window is created by the `dotter()` function in `dotter.c`. `dotter()` also does any initialisation required and creates a main dotter context (a struct containing properties for the current dotter session). Note that there can be multiple Dotter windows for the same Dotter session. Each Dotter window has a separate context for Dotter-wide properties pertaining only to that particular Dotter. Multiple Dotters in the same session will share the same input sequences, for example, but may display different ranges of those sequences. A new Dotter can be opened from an existing Dotter by middle-dragging to select a range to open the new Dotter on.

A Dotter window comprises the following components:

- The menu bar- The dot-matrix plot- Exons/introns for the active strand- Exons/introns for the other strand

### The dot-matrix plot

`dotplot.c, dotterKarlin.c`

This is the main portion of the Dotter window and shows the actual dot-plot or 'pixelmap'. The 'pixelmap' that represents the dot-plot is actually just an array whose length is the image width times the image height. The calculation of this array can be intensive for large sequences, but it only needs to be calculated once on startup (or if the settings are changed). The pixelmap is transformed to a GdkImage using info the Greyramp Tool. The image is then drawn by the dot-plot widget's expose function, along with the grid markers, labels and other cosmetic paraphernalia.

By default, a crosshair is displayed on the dot-plot to indicate the currently-selected coordinate and can be moved by left-clicking or by pressing the arrow keys.

#### HSPs

This is the High Scoring Pair view. It is off by default, but can be toggled by the user or by a command-line argument. HSPs are high-scoring alignments that have known start and end coordinates - they are drawn as a straight, solid line between those coordinates. There are three different modes for the HSP view:

- Red lines: in this mode, all HSPs are drawn as red lines. The HSPs are drawn over the top of the standard dot-plot image.- Score-based: in this mode, HSPs are drawn in different colors depending on their score. The HSPs are drawn over the top of the standard dot-plot image.- Greyramp: in this mode, the HSPs are drawn in a shade of grey based on their score (by default, the darker the shade the higher the score). Greyramp-tool filtering applies. In this mode, the HSP image REPLACES the standard dot-plot image.

### The exon/intron view

This is a drawing area showing the exons and introns within the Dotter display range for a particular strand. Two exon views are used - the 'active' strand exon view is shown first, with the other strand underneath it. The 'active' strand is the strand of the reference sequence that was passed to Dotter. The exons and introns are lined up with the scale on the dot-plot, and by default the crosshair extends over the exon view.

### Saving a dot-plot

A dot-plot can be saved. Dotter dumps chunks of data (whose size is known) to a binary file. If the size of any data changes, the file format must be changed. Old file formats should still be supported. A saved dot-plot can be loaded by calling Dotter from the command line with the `-l` argument.

## The Greyramp Tool

`greyramp.c`

This window is associated with a particular Dotter window and controls the filtering of noise on the dot-plot. The Greyramp Tool is owned by the Dotter window and will be destroyed along with it.

## The Alignment Tool

`alignment.c`

This window is associated with a particular Dotter window. It shows the sequence data for both of the sequences at the currently-selected coordinate in the Dotter window, and highlights the sequences to indicate matching/mis-matching bases. The Alignment Tool window is owned by the Dotter window and will be destroyed along with it.

## Batch mode

Calculating a dot-plot can take a long time. Batch mode allows Dotter to be run in the background. The dot-plot is saved to file and can be loaded by running Dotter from the command-line with the `-l` argument.
