# Supplementary material for: SeqTools: visual tools for manual analysis of sequence alignments
Source: BMC Res Notes. 2016 Jan 22;9:39. doi: 10.1186/s13104-016-1847-3 (PMC4724122; doi:10.1186/s13104-016-1847-3)
Supplement: Supplementary file 1 — 10.1186/s13104-016-1847-2 A tarball of the current production release of the SeqTools source code at the time of writing. [file 13104_2016_1847_MOESM1_ESM.gz › seqtools-4.32.1/doc/Design_notes/modules/seqtoolsUtils.html]

SeqTools - seqtoolsUtils


# seqtoolsUtils

The seqtoolsUtils module contains code that is common to both Blixem and Dotter. The more significant functionality is described below.

## Features

`blxmsp.h/.c`

The seqtoolsUtils module defines structs for our features. Supported features include matches, exons, introns, SNPs, polyA tails and polyA signals.

## File parsing

`blxparser.h/.c, blxGff3parser.h/.c`

Functions to parse the features file. The recommended file format is GFF version 3. However, the older exblx/seqbl file formats (as output from MSPcrunch) are also supported. Note that the reference sequence can be passed as part of the features file or in a separate file - if the latter, then it is parsed separately by the main() function, not by this module.

## Sequence translation

`translate.c, iupac.h`

Defines various functions and matrices used to reverse/complement nucleotide sequences and to translate nucleotide sequences to peptides.

## Web browser

`seqtoolsWebBrowser.c`

Defines functions to open a URL in the user's default web browser.

## Coordinate conversions

`utilities.c`

There are two functions that underpin all conversion of coordinates between how they are displayed and how they are stored: `convertDisplayIdxToDnaIdx` and `convertDnaIdxToDisplayIdx`. They convert a DNA index to display coords and vice versa. The display coords are either DNA or peptide coords depending on whether Blixem is in nucleotide or protein mode. These functions also take care of inverting the coords if the display is reversed (i.e. if the reverse strand is active) - this is necessary because the GtkAdjustment for the scrollbar can only handle a forward range, i.e. it assumes values increase from left-to-right; if we want to display values as decreasing from left-to-right then we need to invert the values.

## Drawing and Printing

`utilities.c`

Printing in GTK+ is performed using the Cairo graphics package. We must provide some way of telling Cairo what to draw\*. We implement this as follows:

- Each widget's expose function draws to a pixmap (i.e. GdkDrawable) and then pushes this pixmap to the screen. The pixmap is cached by setting it as a property on the widget using `widgetSetDrawable`. If the cached pixmap already exists, the expose function will not re-create it; it will just push the existing pixmap to the screen. (To force the widget to re-draw itself you must therefore clear the cached pixmap, using the `widgetClearCachedDrawable` function.)- The cached pixmap can be retrieved using the `widgetGetDrawable` function. The pixmap can be printed by Cairo, so this provides a way to print the contents of an individual widget. Only widgets that have a cached pixmap will be printed.- In order to print multiple widgets (e.g. all of the child widgets in a container such as the top-level window), we must combine the pixmaps of the individual widgets. We do this by recursing through all of the child widgets, extracting their cached pixmaps (if they have one), and drawing them onto a single, overall pixmap. Their position on the main pixmap can be found by translating the top-left corner position from the widget's coordinate system (0,0) to the overall parent widget's coordinate system. (See the `collatePixmaps` function.)- The print operation is initiated using the `blxPrintWidget` function. This creates a GtkPrintOperation and sets the print-settings and page-setup for it, if given (i.e. these can be cached by the calling function so that the same settings can be used the next time a print is performed). It then runs the print operation, which opens the print dialog. When the user OKs the dialog, this function updates the input print settings with the returned settings from the dialog.- When the user OKs the print dialog, the `onBeginPrint` callback is called. This function does any preparation required, e.g. setting the number of pages required.- Next, `onDrawPage` is called to do the drawing for each page.

\*UPDATE: A lot of the functions used here are now deprecated. It looks like GTK has improved support for drawing widgets' windows directly, which probably means that all of the pixmap stuff mentioned here is no longer necessary.

Current issues with this are:

- We always assume a single page and scale the entire image down to fit (this is usually adequate for Blixem and Dotter).- Text quality is really poor.- Items in a scrollable window are not clipped to the visible region; this is generally not an issue because other widgets will be drawn over the unwanted regions. However, if not all of the widgets are drawn, some of the unwanted regions may be visible.
