# Supplementary material for: SeqTools: visual tools for manual analysis of sequence alignments
Source: BMC Res Notes. 2016 Jan 22;9:39. doi: 10.1186/s13104-016-1847-3 (PMC4724122; doi:10.1186/s13104-016-1847-3)
Supplement: Supplementary file 1 — 10.1186/s13104-016-1847-2 A tarball of the current production release of the SeqTools source code at the time of writing. [file 13104_2016_1847_MOESM1_ESM.gz › seqtools-4.32.1/doc/Design_notes/specs.html]

SeqTools - Functional Spec


# Functional specification

We don't have a formal functional specification for the SeqTools programs; instead, required behaviours are defined by means of a test plan, which specifies a set of testable actions and their expected outcomes. The test plan is an OpenOffice spreadsheet. To get a general overview of program behaviour, see the user documentation.

- Test plan (.ods file)
- User documentation
