# Supplementary material for: SeqTools: visual tools for manual analysis of sequence alignments
Source: BMC Res Notes. 2016 Jan 22;9:39. doi: 10.1186/s13104-016-1847-3 (PMC4724122; doi:10.1186/s13104-016-1847-3)
Supplement: Supplementary file 1 — 10.1186/s13104-016-1847-2 A tarball of the current production release of the SeqTools source code at the time of writing. [file 13104_2016_1847_MOESM1_ESM.gz › seqtools-4.32.1/doc/User_doc/Belvu_manual/Belvu_manual.pdf]

Belvu User Manual

Written by Gemma Barson
<gb10@sanger.ac.uk>

Wellcome Trust Sanger Institute
22 September 2011

Revision History

**Revision**	**Date**	**Author**
First revision (Belvu v4.4.1)	18/01/11	Gemma Barson
Updated for version 4.14	02/07/12	Gemma Barson

Contents

Revision History **2**

Introduction **4**

Getting Started **5**
- Running Belvu 5
- File formats 5
  - Selex 5
  - Stockholm 5
  - MSF 6
  - Fasta 6
  - Raw 6

The Belvu Windows **6**
- Main window 7
  - Selections 7
  - Fetching sequences 8
  - Toolbar 8
  - Find dialog 9
- Tree 9
  - Tree menu 10
  - Organisms window 11
  - Tree settings 11
- Conservation plot 12
  - Conservation plot menu 12
  - Conservation plot settings 13

Main menu **14**
- File menu 14
- Edit menu 16
- Color menu 17
- Settings menu 18
- Help menu 19

Keyboard shortcuts **20**

Introduction

This manual explains how to configure, run and use Belvu. Belvu is a multiple sequence alignment viewer and phylogenetic tool. It has an extensive set of user-configurable modes to color residues by conservation or by residue type, and some basic alignment editing capabilities. It can generate distance matrices between sequences and construct distance-based trees, either graphically or as part of a phylogenetic software pipeline.
Key features include:

- Residues can be coloured by conservation, with user-configurable cutoffs and colours.
- Residues can be coloured by residue type (user-configurable).
- Colour schemes can be imported or exported.
- Swissprot (or PIR) entries can be fetched by double clicking.
- The position in the alignment can be easily tracked.
- Simple editing commands for rows and columns is supported (although Belvu is not intended to be a full editor).
- The alignment can be saved in Stockholm, Selex, MSF or FASTA format.
- Distance matrices between sequences can be generated using a variety of distance metrics.
- Distance matrices can be imported or exported.
- Trees can be constructed based on various distance-based tree reconstruction algorithms.
- Trees can be saved in New Hampshire format.
- Belvu can perform bootstrap phylogenetic reconstruction.
- Belvu can be used as a graphical tree viewer, or as a command-line tool for use in phylogenetic software pipelines.

Belvu is maintained by the Wellcome Trust Sanger Institute and is available as part of the SeqTools package. The software can be downloaded from the Sanger Institute's website:
http://www.sanger.ac.uk/resources/software/seqtools.

Getting Started

Running Belvu

As a minimum, Belvu takes the following required arguments:

```
belvu <alignment_file>
```

where <alignment_file> is a file or pipe containing the multiple alignment in Stockholm, Selex, MSF or aligned-Fasta format (see below).
Run 'belvu' without any arguments to see brief usage information, or, for more detailed help, run:

```
belvu --help
```

File formats

Belvu currently supports Stockholm (Mul/Pfam), Selex, MSF and aligned- and unaligned Fasta formats. Belvu will automatically detect which file format is supplied. The 'raw' file format can also be used, but you must pass a raw file using the `-r` argument because Belvu cannot detect this format automatically.

Selex

Selex is the native format used by Sean Eddy's HMM package HMMER. For details, see: http://www.psc.edu/general/software/packages/hmmer/manual/node46.html.

Each line contains a name, followed by the aligned sequence. A space, dash, underscore, or period denotes a gap. If the alignment is too long to fit on one line, the alignment is split into multiple blocks, separated by blank lines. The number of sequences, their order, and their names must be the same in every block (even if a sequence has no residues in a given block!) Other blank lines are ignored. You can add comments to the file on lines starting with a #.

```
seq1 \ \ \ \ ACGACGACGACG.
seq2 \ \ \ \ ..GGGAAAGG.GA
seq3 \ \ \ \ UUU..AAAUUU.A
seq1 \ ..ACG
seq2 \ AAGGG
seq3 \ AA...UUU
```

Stockholm

Also known as “Mul” or “Pfam” format, Stockholm is the native format used by Pfam and Rfam to disseminate protein and RNA sequence alignments. The file must start with a line giving the format version, and end with `//`. It has one domain per line:

```
# STOCKHOLM 1.0
<sequence_name>/<start>-<end> <sequence>
...
//
```

The residues must be aligned and gaps should be represented by dots. Markup lines can also be included; see http://en.wikipedia.org/wiki/Stockholm_format for more details.

MSF

Note on the MSF format: The "..... Check: .." line has to come before the first line that does not start with a space. The only legal exception is the line "PileUp of:" from GCG programs.

```
[Pileup]

[<filename>]   MSF:  <len>  Type: <type>    Check:  <check>   ..

 Name: <name1>  Len:  <len>  Check:  <check>  Weight:  <weight>
 Name: <name2>  Len:  <len>  Check:  <check>  Weight:  <weight>
 ...
//
<name1>      <sequence>
<name2>      <sequence>
...
<name1>      <sequence>
<name2>      <sequence>
...
```

The sequence names can include coordinates, e.g.

```
<name>/<start>-<end>
```

Fasta

In Fasta format, the sequence name is on a line starting with `>`, and the sequence on the following line(s). Input files for Belvu must be in aligned-Fasta format, where gaps are included so that each sequence is the same length.

```
>seq1
ACGACGACGACG.
..ACG
>seq2
..GGGAAAGG.GA
AAGGG
```

Belvu does not accept unaligned-Fasta files as input, but can output the sequences in unaligned Fasta format (i.e. with gaps removed)

Raw

The raw file format is as follows. Raw files must be passed using the `-r` command line argument because Belvu cannot detect this file format automatically.

```
<name> <sequence>
<name> <sequence>
...
```

The Belvu Windows

Main window

The main Belvu window contains the alignments. Residues are coloured by conservation or by residue type; use the Color menu to change the colour scheme.

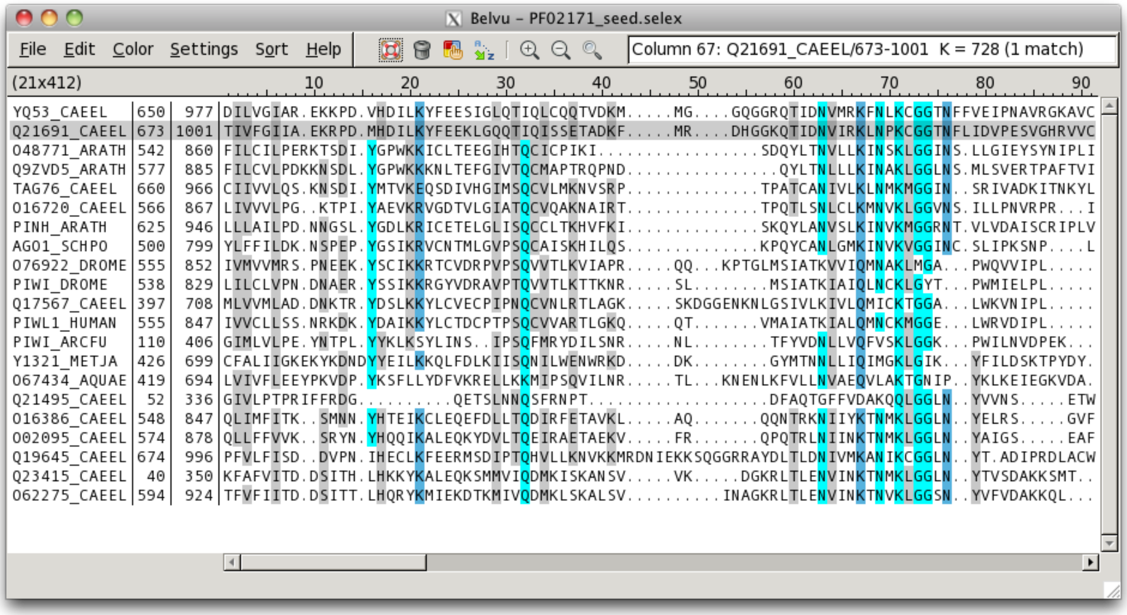

Figure 1: Alignment window in colour-by-conservation mode

At the top of the alignment list is a header displaying the number of sequences and alignment length, e.g.

```
(21x412)
```

means there are 21 sequences and the alignment length is 412.

The alignment list contains the following columns:

**Name**	The sequence name
**Start**	The start coordinate in the match sequence
**End**	The end coordinate in the match sequence
**Score**	Only displayed if a scores file was loaded; displays the score of the sequence
**Sequence**	Displays the sequence data

Selections

Click on a row to select that alignment. Details about the selected row will be shown in the feedback box on the toolbar. If there are other sequences with the same name, their names will be highlighted in the alignment list (but only the clicked row will have the whole row highlighted). The number of matches is shown in brackets in the feedback box.
If you clicked within the sequence area, a column will also be selected; the column number (1-based from the left) that you clicked will be shown in the feedback box, along with the residue and the sequence coordinate at that column for the selected sequence.

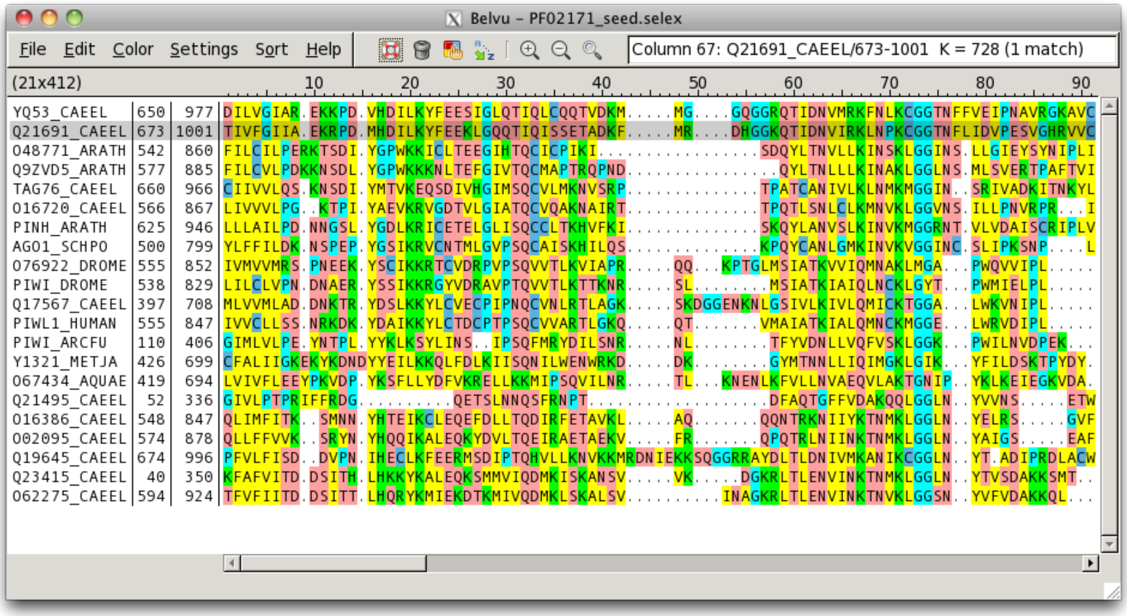

Figure 2: Alignment window in colour-by-residue mode

Middle-click in the alignment in order to select a column; the current column will be highlighted while the middle button remains pressed and you can drag to other columns to see column information dynamically. When you release the mouse button, the display will scroll so that it is centered on the selected column.

Fetching sequences

Double-click on a row in the alignment to fetch that sequence; the program used to fetch sequences must be specified in the BELVU_FETCH environment variable before Belvu is opened, e.g. in a C shell terminal:

```
setenv BELVU\_FETCH {\textquotesingle}pfetch -F{\textquotesingle}
```

Toolbar

The toolbar contains shortcuts to several of the menu items, as well as a feedback area displaying information about the currently-selected row and/or column.

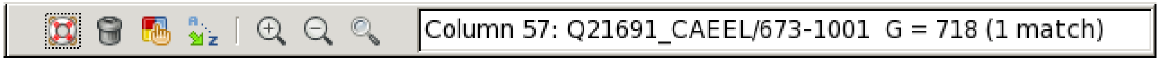

Figure 3: The toolbar

The toolbar buttons are as follows:

Help Display the help pages. See the Help menu

**Remove many sequences**	Start the mode that allows you to double-click to remove sequences. Click again or press Esc to cancel this mode. See the Edit menu
**Edit current colour scheme**	Edit the current colour scheme (see the Color menu)
**Sort alphabetically**	Sort sequences by name (see the Sort menu)
**Zoom in**	Increase the font size in the alignment list
**Zoom out**	Decrease the font size in the alignment list
**Find**	Open the Find dialog

The feedback area on the toolbar displays the following information:

**Column <column>:**	If a column is selected, this displays the column number (1-based from the left-most column)
**<name>/<start>-<end>**	If a sequence is selected, this displays the sequence name and its start/end coordinates
**<residue> = <coord>**	If a column and sequence are selected, this displays the residue and coordinate of that column within that sequence
**(<n> match[s])**	If a sequence is selected, this shows the number of sequences in the alignment with the same name (1 => only the current sequence has that name)

Find dialog

The Find dialog allows you to search for sequences by name. Open it by clicking on the toolbar icon or by using the keyboard shortcut Ctrl-F.

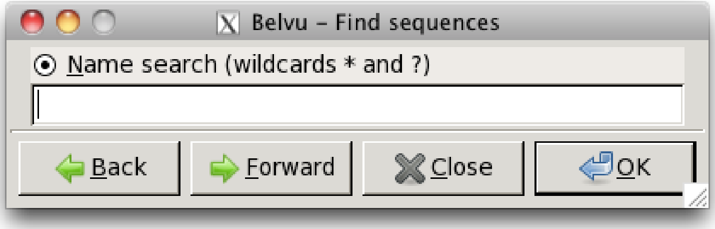

Figure 4: Find-sequences dialog

Enter the text you wish to search for. The text can include the wildcards '*' (for any amount of any character) or '?' (for one occurrence of any character).
Hit OK to close the dialog and search. If found, the first matching result will be highlighted in the alignment list. Alternatively, click Forward or Back on the Find dialog to perform a search forwards or backwards from the last search result. (These operations will start from the beginning of the list if there was no previous search result.)

Tree

The tree window can be opened from the main window using the 'Show tree' option on the File menu. The tree window will show a distance-based phylogenetic tree of the current alignment

using the default settings. To edit the tree settings before calculating the tree, first select the 'Tree settings' option from the File menu.
Click on a sequence name to select a sequence in the tree; the sequence will be highlighted in both the tree and the main window.
Click on a branch to either swap the nodes or re-root the tree from that branch; see the Tree settings section for more details.

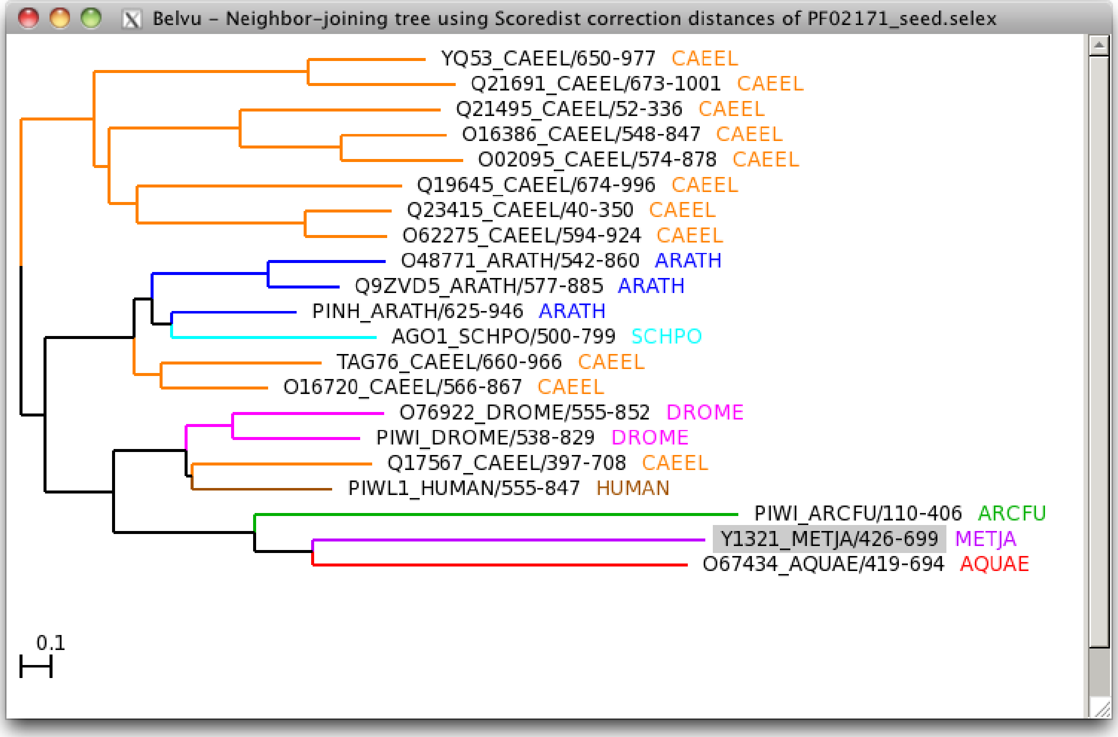

Figure 5: Tree window

Tree menu

The tree menu can be accessed by right-clicking anywhere in the tree window.

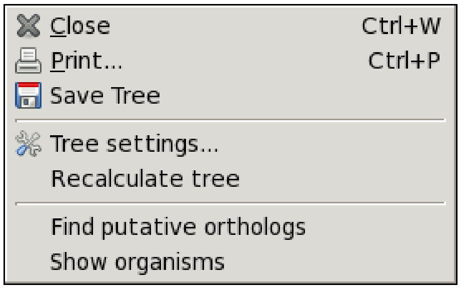

Figure 6: Tree menu

The options on the tree menu are as follows:

**Close**	Close the tree window (the tree will not be deleted and can be opened again without recalculating)
**Print**	Print the tree window
**Save Tree**	Save the tree in New Hampshire format
**Tree settings**	Open the tree settings dialog
**Recalculate tree**	Forces the tree to be recalculated; this is required after the alignment has changed and the tree is now invalid (e.g. if rows have been deleted)
**Find putative orthologs**	Highlights putative orthologs in the tree and outputs their details to the terminal
**Show organisms**	Opens a window showing the list of organisms, and outputs the number of organisms to the terminal

Organisms window

Select 'Show organisms' from the right-click menu in the tree to display the organisms window, which lists all of the organisms in the alignment:

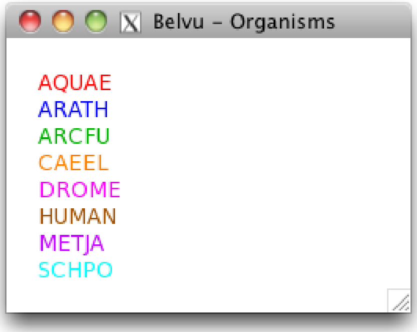

Figure 7: Organisms window

Tree settings

To open the tree-settings dialog, use the 'Tree settings' option from the File menu on the main window or from the right-click menu on the tree window.
The options are as follows. Note that changing the tree building method or distance correction method will force the tree to be recalculated, which may take a long time for large alignments.

**Tree building method**	Choose whether the tree should be built using the neighbour-joining or UPGMA method
**Distance correction method**	Select the distance-correction method to use
**Tree scale**	Adjust the horizontal scale used to draw the tree; set a smaller number to decrease the width of the tree or a larger number to increase it.

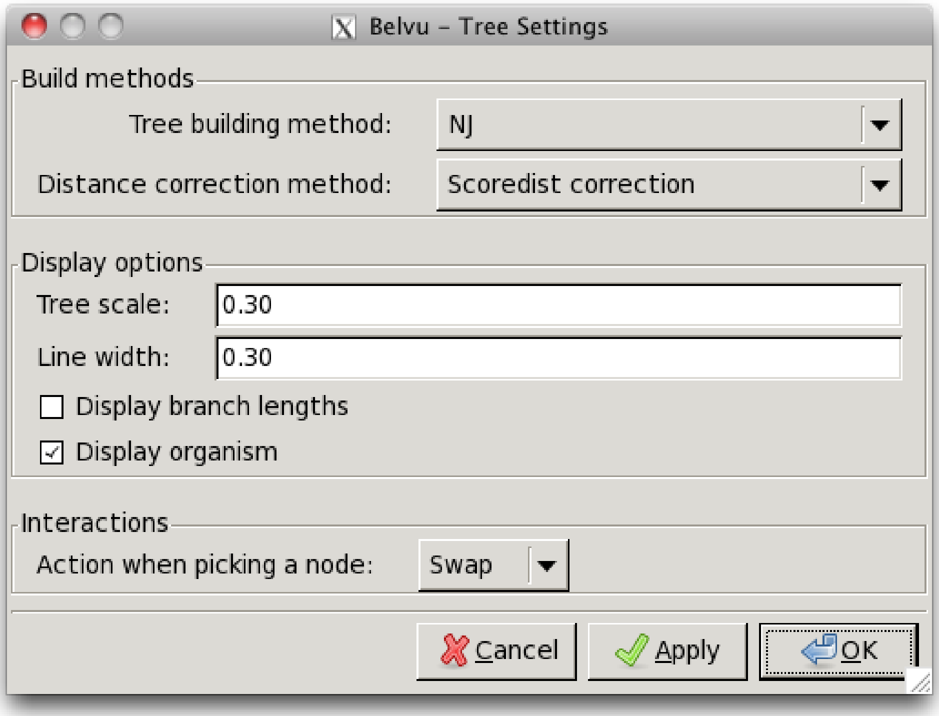

Figure 8: Tree settings dialog

**Line width**	Set the line width to use for the branches (0.1 => 1 pixel)
**Display branch lengths**	Whether to label branches with their lengths
**Display organism**	Whether to display the organism next to the sequence name
**Action when picking a node**	Swap: when you click a branch, its two child nodes will be swapped<br>Reroot: when you click a branch, the tree will be re-rooted with that node as the root<br>Note: to revert to the original tree, select the 'Recalculate tree' option from the right-click menu

Conservation plot

To display the conservation profile, select 'Show conservation plot' from the File menu. The conservation profile window will open displaying a plot of the conservation (vertical axis) against the column numbers (horizontal axis). The average conservation is shown as a red line.

Conservation plot menu

Right-click anywhere on the conservation plot to display the menu:
The options are:

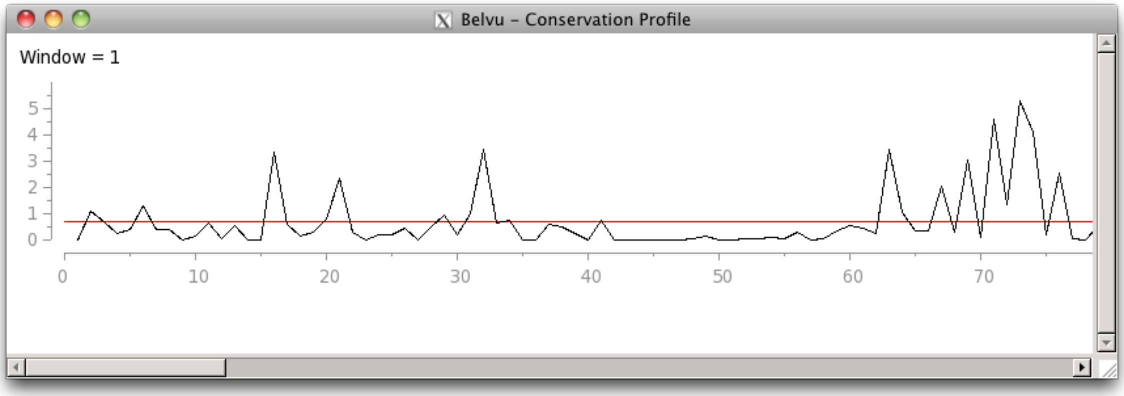

Figure 9: Conservation plot

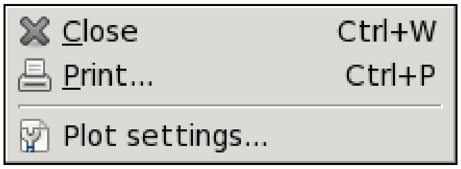

Figure 10: Conservation plot menu

**Close**	Close the conservation plot window
**Print**	Print the conservation plot
**Plot settings**	Show the plot settings dialog

Conservation plot settings

Select the 'Plot settings' option from the right-click menu on the conservation plot to show the plot settings dialog:

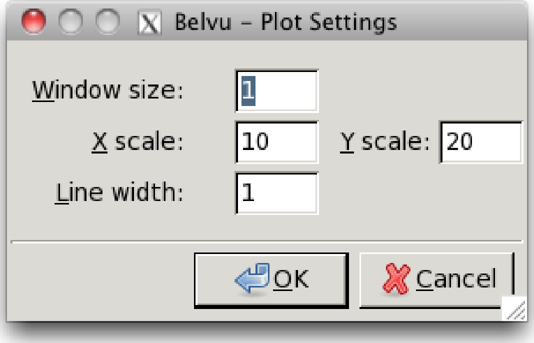

Figure 11: Conservation plot settings

The options are:

Window size Specify the size of the sliding window used to smooth out the curve; set a larger value for a smoother curve. The minimum value is 1, which means no smoothing is done

X scale Adjust the scale of the horizontal axis; set a smaller value to compress the scale or a larger value to expand it

Y scale Adjust the scale of the vertical axis; set a smaller value to compress the scale or a larger value to expand it

Line width Set the line width to use for the drawing, in pixels

Main menu

The main menu can be accessed via the menu-bar at the top of the main window. Right-clicking in the main window is a shortcut to the File menu.
Note that menus with a dotted line at the top can be "torn off" by clicking on the dotted line. A torn-off menu will stay visible on top of the Belvu window and can be repositioned by dragging its header bar. Click the dotted line again to get rid of it.

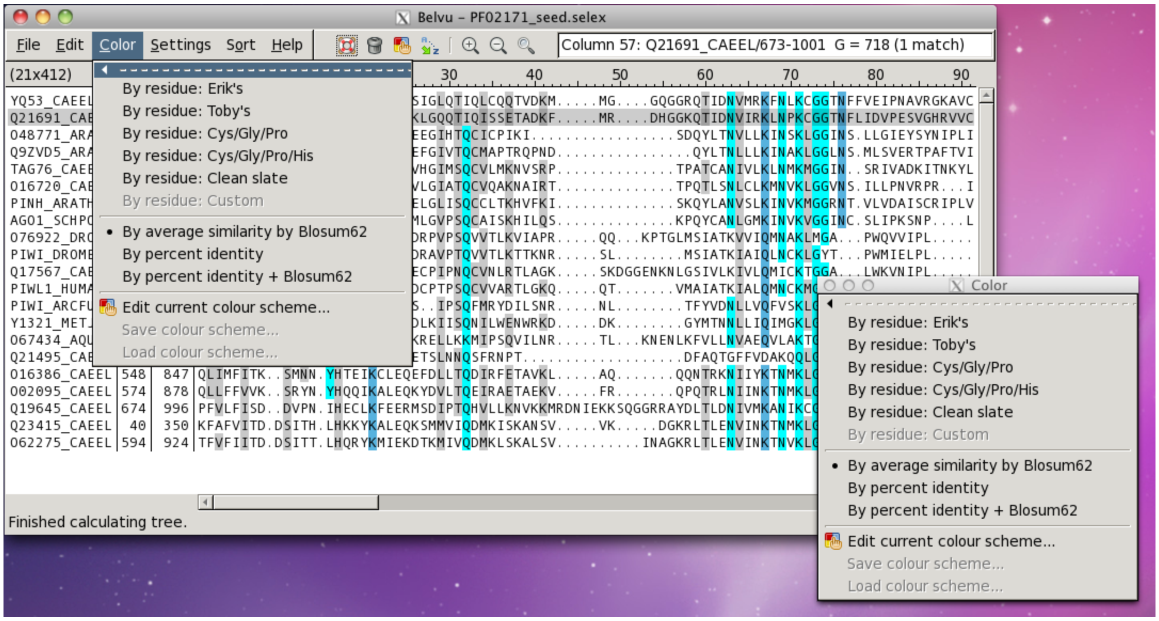

Figure 12: Menu tear-offs

File menu

Quit Quit Belvu (close all windows and exit)

Wrap for printing Open a window showing a wrapped alignment, suitable for printing

Print Print the current window (note that you should use the print option from the wrapped-alignment window to print the wrapped view)

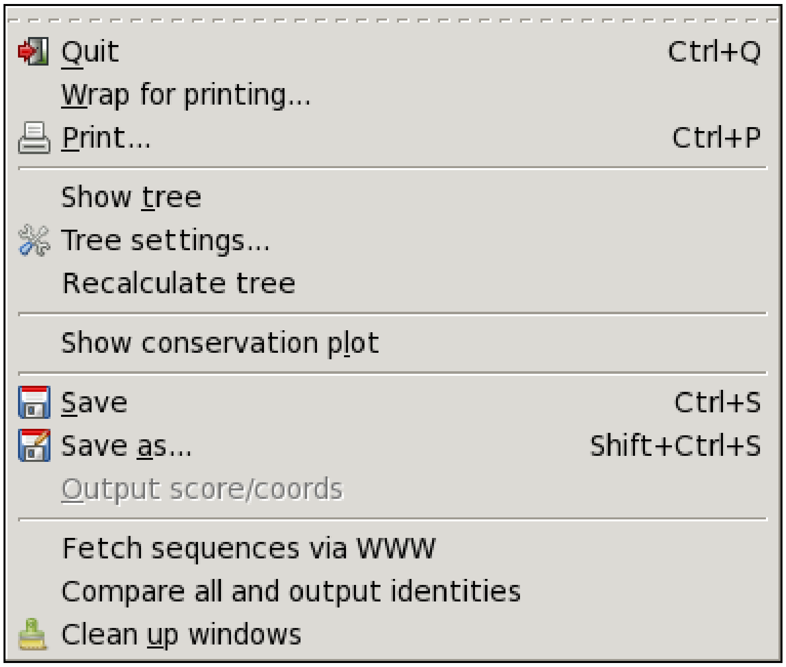

Figure 13: File menu

**Show tree**	Open the tree window; calculates the tree if it has not yet been calculated
**Tree settings**	Edit the settings used to calculate and display the tree
**Recalculate tree**	Use this to recalculate the tree after making changes that invalidate it, e.g. deleting rows
**Show conservation plot**	Show the conservation plot window
**Save**	Save the alignment in the current format
**Save as**	Save the alignment; allows you to select a different file format and choose whether coordinates should be saved and what separator character to use
**Output score/coords**	Only applicable if scores are loaded; outputs the score and coordinates of the currently-selected sequence to the terminal
**Fetch sequences via WWW**	Enables fetching of sequences over HTTP
**Compare all and output identities**	Compares each sequence against each other and outputs their identity and score to the terminal, along with some summary information about the maximum, minimum and mean score and identity
**Clean up windows**	Close all windows opened by this instance of Belvu (does not close the main window)

Edit menu

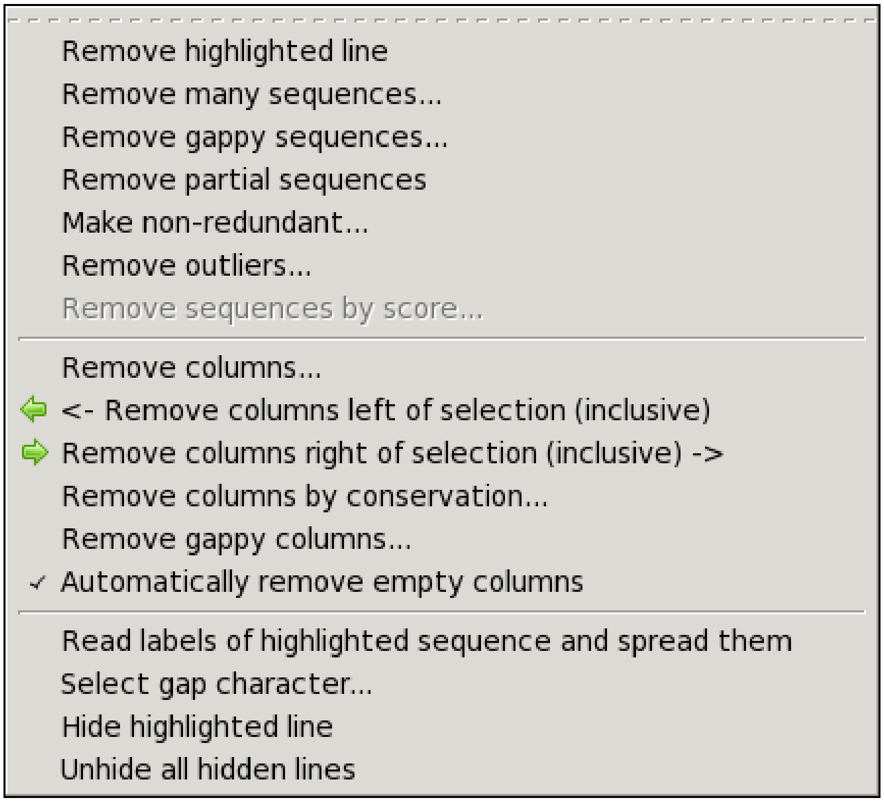

Figure 14: Edit menu

**Remove highlighted line**	Remove the currently-selected line
**Remove many sequences**	Enables a mode where you can double-click on sequences to remove them. The cursor will change to indicate that you are in this mode. Select the option again, press the Esc key, or right-click to cancel this mode
**Remove gappy sequences**	Remove sequences that have more than a given percentage of gaps
**Remove partial sequences**	Removes partial sequences
**Make non-redundant**	Remove sequences that are more than a given percentage identical to any other
**Remove outliers**	Remove sequences that are less than a given percentage identical to any other

**Remove sequences by score**	Only applicable if scores are loaded; remove sequences that have a score lower than a given threshold
**Remove columns**	Remove a specific range of columns
**Remove columns left of selection**	Removes the columns to the left of the currently-selected column (which is displayed in the feedback box on the toolbar, if a column is selected). The operation is inclusive, so the currently-selected column will be removed as well
**Remove columns right of selection**	Removes the columns to the right of the currently-selected column. The operation is inclusive, so the currently-selected column will be removed as well
**Remove columns by conservation**	Remove columns with a maximum conservation between specified values
**Remove gappy columns**	Remove columns with more than a given percentage of gaps
**Automatically remove empty columns**	After deleting sequences, columns that are left empty are automatically removed if this option is enabled
**Read labels of highlighted sequence and spread them**	Undocumented
**Select gap character**	Change the character used to display gaps in the alignment
**Hide highlighted line**	Hides the currently-selected line
**Unhide all lines**	Show all lines that were previously hidden

Color menu

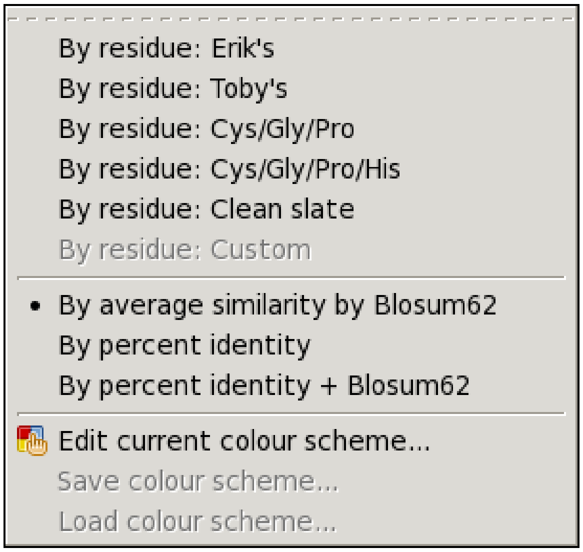

Figure 15: Colour menu

**Erik's**	Use Erik's original built-in residue colour scheme

Toby's Another built-in residue colour scheme

Cys/Gly/Pro A colour-by-residue scheme where only cystine, glycine and proline are highlighted

Cys/Gly/Pro/His A colour-by-residue scheme where only cystine, glycine, proline and histidine are highlighted

Clean slate Clear all colours; used for when you want to create a new colour scheme starting with all colours being white

Custom This option will become enabled when a residue colour scheme has been customised by editing it or loading it from file; if you change to a different colour scheme, you can toggle back to the custom colour scheme by selecting this option

By average similarity by Blosum62 A colour-by-conservation scheme colouring by average similarity by Blosum62

By percent identity A colour-by-conservation scheme colouring by percent identity

By percent identity + Blosum62 A colour-by-conservation scheme colouring by both percent identity and average similarity by Blosum62

Edit current colour scheme Edit the current colour scheme. If in colour-by-residue mode, allows you to edit the residue colours; if in colour-by-conservation mode, allows you to edit the thresholds and colours for the different levels of conservation

Save colour scheme Only applicable in colour-by-residue mode; save the current colour scheme to file

Load colour scheme Only applicable in colour-by-residue mode; load a colour scheme from file

Settings menu

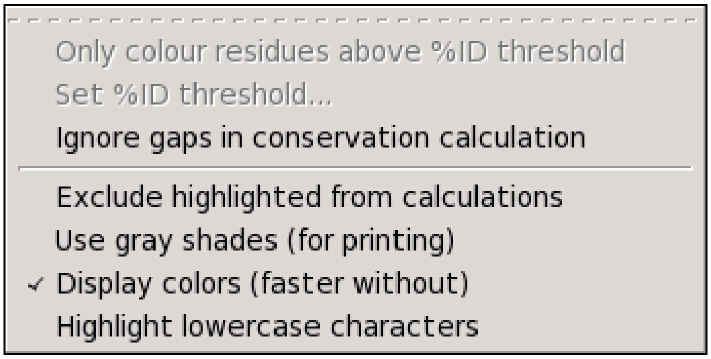

Figure 16: Settings menu

Only colour residues above %ID threshold Only applicable in colour-by-residue mode; only colour residues that have a percent identity above the threshold specified by the 'Set %ID threshold' menu option

**Ignore gaps in conservation calculation**	Only applicable in colour-by-conservation mode; ignore gaps when calculating the conservation
**Exclude highlighted from calculations**	Exclude the currently-selected row from colour calculations
**Use gray shades**	Only applicable to colour-by-conservation mode; use grey shades (suitable for printing)
**Display colours**	Whether to show colours or not (faster without)
**Highlight lowercase characters**	Highlights lowercase characters

Help menu

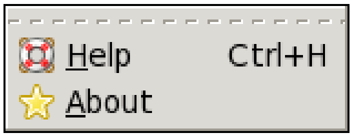

Figure 17: Help menu

**Help**	Show the help pages
**About**	Show the 'About' dialog

Keyboard shortcuts

Recommended shortcuts (consistent with other SeqTools programs):

**,**	Scroll one column left
**.**	Scroll one column right
**Ctrl-,**	Scroll one page left
**Ctrl-.**	Scroll one page right
**Shift-Ctrl-,**	Scroll to leftmost column
**Shift-Ctrl-.**	Scroll to rightmost column
**PageUp**	Scroll one page up
**PageDown**	Scroll one page down
**Ctrl-up**	Scroll one row up
**Ctrl-down**	Scroll one row down
**Home**	Scroll to top of alignment list
**End**	Scroll to bottom of alignment list
**Ctrl-W**	Close the current window. If this is the main window, it quits the application
**Ctrl-Q**	Quit the application
**Ctrl-S**	Save the alignment in the current format
**Shift-Ctrl-S**	Save the alignment in a different format
**Ctrl-P**	Print the current window
**Ctrl-H**	Open the Help pages
**Ctrl-F**	Find sequences
**Ctrl-R**	Make non-redundant
**Ctrl-T**	Remove partial sequences
**t**	Toggle between colour-by-residue and colour-by-conservation mode
**= (equal)**	Zoom in
**- (minus)**	Zoom out

Old-style Belvu shortcuts:

**Left-arrow**	Scroll one page left
**Right-arrow**	Scroll one page right
**Ctrl-left**	Scroll one column left
**Ctrl-right**	Scroll one column right
**Up-arrow**	Scroll one page up
**Down-arrow**	Scroll one page down
**Insert**	Scroll to leftmost column
**Delete**	Scroll to rightmost column
