# Supplementary material for: SeqTools: visual tools for manual analysis of sequence alignments
Source: BMC Res Notes. 2016 Jan 22;9:39. doi: 10.1186/s13104-016-1847-3 (PMC4724122; doi:10.1186/s13104-016-1847-3)
Supplement: Supplementary file 1 — 10.1186/s13104-016-1847-2 A tarball of the current production release of the SeqTools source code at the time of writing. [file 13104_2016_1847_MOESM1_ESM.gz › seqtools-4.32.1/doc/User_doc/belvu_quick_start.html]

Belvu - Quick Start


# Belvu - Quick Start

- What's new
- Overview
- Controls
- Styles

## Overview

Belvu - a multiple alignment viewer.

This page gives a quick-start guide to using Belvu. Other documentation is available here:

- What's new (revision history)
- Usage (command line options)
- User manual
- Outstanding issues

## Controls

### Mouse

#### Left mouse button

- Click on a residue to select the row and column (current selection will be shown in the feedback box at the top).
- Double click on a sequence to fetch.
- Scrollbars: drag and page.

#### Middle mouse button

- In alignment: select column and display crosshair. Drag to move the crosshair; release to centre on column.
- Scrollbars: drag and jump.

#### Right mouse button

- File menu (or Text menu when in the feedback box on the toolbar).

#### Mouse wheel

- You can scroll horizontally and vertically using your mouse's scroll wheel, if it has these functions.

### Keyboard shortcuts

#### Application

- Ctrl-Q: Quit application
- Ctrl-W: Close current window
- Ctrl-H: Show this help page
- Ctrl-S: Save alignment
- Shift-Ctrl-S: Save alignment as
- Ctrl-P: Print
- Ctrl-F: Find

#### Display

- T: Toggle color mode
- = (equal): Zoom in (increase text size)
- - (minus): Zoom out (decrease text size)

#### Editing

- Ctrl-R: Make non-redundant
- Ctrl-T: Remove partial sequences

#### Navigation

- Arrow keys: Scroll one page up/down/left/right
- Ctrl and Arrow keys: Scroll one row or column up/down/left/right
- PageUp: Scroll one page up
- PageDown: Scroll one page down
- Home: Go to Top
- End: Go to Bottom
- Insert: Go to Start of line
- Delete: Go to End of line
- , (comma): Scroll one column left
- . (period): Scroll one column right
- Ctrl-comma: Scroll one page left
- Ctrl-period: Scroll one page right
- Shift-Ctrl-comma: Go to Start of line
- Shift-Ctrl-period: Go to End of line

## Styles

You can change the look of GTK programs such as Belvu using the gtkrc file. Create or edit the file called `.gtkrc-2.0` in your home directory. Here are some suggestions for styles that you can modify:

```
# Change the default font size
gtk-font-name = "san serif 12"

style "scrollbar-style"
{
# Change the color of the scrollbars
        bg[NORMAL] = "#82cafa"
        bg[INSENSITIVE] = "#82cafa"
        bg[PRELIGHT] = "#97daff"

# Change the size of the scrollbars
        GtkRange::slider-width=12
}

class "*Scrollbar*" style "scrollbar-style"
```
