# Supplementary material for: SeqTools: visual tools for manual analysis of sequence alignments
Source: BMC Res Notes. 2016 Jan 22;9:39. doi: 10.1186/s13104-016-1847-3 (PMC4724122; doi:10.1186/s13104-016-1847-3)
Supplement: Supplementary file 1 — 10.1186/s13104-016-1847-2 A tarball of the current production release of the SeqTools source code at the time of writing. [file 13104_2016_1847_MOESM1_ESM.gz › seqtools-4.32.1/doc/User_doc/Blixem_manual/Blixem_manual.pdf]

Blixem User Manual

Written by Gemma Barson
<gb10@sanger.ac.uk>

Wellcome Trust Sanger Institute
17 January 2011

Revision History

Revision	Date	Author
First revision (Blixem v4.1.5)	17/01/11	Gemma Barson
Updated for Blixem v4.1.9	14/02/11	Gemma Barson
Updated for Blixem v4.1.13	25/03/11	Gemma Barson
Updated for Blixem v4.1.14	05/04/11	Gemma Barson
Updated for Blixem v4.1.17	09/05/11	Gemma Barson
Updated for Blixem v4.2	17/06/11	Gemma Barson
Updated for Blixem v4.7	02/12/11	Gemma Barson
Updated for Blixem v4.14	15/06/12	Gemma Barson
Updated for Blixem v4.26	07/03/14	Gemma Barson
Updated for Blixem v4.27	17/04/14	Gemma Barson

Contents

Revision History 2

Introduction 5
- An aside about the name "Blixem" . . . 5

Getting Started 6
- Running Blixem . . . 6
- Input files . . . 6
  - GFF file . . . 6
  - Transcripts . . . 7
  - Variations . . . 7
  - Sample GFF file . . . 7
  - FASTA file . . . 8
  - Combined GFF and FASTA file . . . 8
- Configuration file . . . 8
  - Program defaults . . . 9
  - Fetch methods . . . 10
  - Data types . . . 12
  - Source mapping . . . 12
  - Sources . . . 13
  - User settings . . . 13
- Colour key file . . . 14

The Blixem Window 15
- Active Strand . . . 15
- Big Picture . . . 17
  - Bumping the transcript view . . . 17
- Detail View . . . 17
  - Match colours . . . 19
  - Alignment lists . . . 19
  - Nucleotide mode . . . 19
  - Protein mode . . . 19
  - Exons . . . 20
- Coverage view . . . 20
- The toolbar . . . 22
- The main menu . . . 23
- Hiding sections of the window . . . 25

Operation 26
- Navigation . . . 26
  - Scrolling . . . 26
  - Zooming . . . 27
- Selections . . . 27
  - Selecting sequences . . . 27
  - Selecting coordinates . . . 28
  - Finding sequences . . . 28
  - Copy and paste . . . 29
- Sorting alignments . . . 30

Fetching sequences . . . 31
Grouping sequences . . . 31
Creating a group from a selection . . . 31
Creating a group from a search . . . 31
Creating a temporary 'match-set' group from the current selection . . . 32
Editing groups . . . 32
Running dotter . . . 33
Reference sequence versus itself . . . 34
Dotter HSPs only . . . 34

Settings **34**
Features . . . 35
Highlight variations . . . 35
Show polyA tails . . . 35
Show Unaligned Sequence . . . 35
Show Colinearity Lines . . . 35
Show Splice Sites . . . 35
Highlight Differences . . . 37
Squash Matches . . . 37
Display . . . 37
Use print colours . . . 37
Font . . . 37
%ID per cell . . . 37
Max %ID . . . 37
Min %ID . . . 37
Depth per cell . . . 37
Columns . . . 37
Load optional data . . . 37
Column settings . . . 37
Appearance . . . 38

Key **38**

Keyboard shortcuts **38**

Introduction

This manual explains how to configure, run and use Blixem. Blixem is an interactive browser of pairwise matches displayed as multiple alignments. It is not strictly a multiple alignment tool, rather a 'one-to-many' alignment. It is used to check the alignments of nucleotide and amino acid sequences against a reference sequence.

Blixem is maintained by the Wellcome Trust Sanger Institute and is available as part of the SeqTools package. The software can be downloaded from the Sanger Institute's website:
http://www.sanger.ac.uk/resources/software/seqtools

An aside about the name "Blixem"

"BLIXEM" was originally an acronym for "BLast matches In an X-windows Embedded Multiple alignment", although this is a bit of a misnomer now because Blixem can handle any kind of alignment, not just BLAST matches. We have dropped the acronym, and the capital letters, so the correct name is just "Blixem".

Getting Started

Running Blixem

As a minimum, Blixem takes the following required arguments:

```
blixem --display-type=N|P <features_file>
```

where `<features_file>` is the path name of a GFF version 3 file containing the alignments and any other features. The ‘`--display-type`‘ or ‘`-t`‘ argument is the only mandatory argument. It defines the display mode: ‘`N`‘ for nucleotide or ‘`P`‘ for protein. Run ‘blixem‘ without any arguments to see further usage information.

Input files

Blixem takes one or two files as input: a mandatory GFF version 3 file containing the features and, optionally, a separate file containing the reference sequence in FASTA format.

```
blixem --t N|P [<reference\sequence\file>] <features_file>
```

If the reference sequence file is not provided, the reference sequence must be supplied in FASTA format at the end of the GFF file, following a comment line that reads ‘##FASTA‘.

Note that the reference sequence must always be a nucleotide sequence and match sequences must be the correct type for the mode, i.e. nucleotide sequences for nucleotide mode or protein sequences for protein mode.

GFF file

Blixem uses the GFF version 3 file format. In this section we give a very brief description of this file format; see http://www.sequenceontology.org/gff3.shtml for a full description.

The GFF file should start with the following two comment lines. (Additional comments can be included but may be ignored.)

```
##gff-version 3
##sequence-region chr4-04 44144 154265
```

Each subsequent line defines a feature. A feature line must have the following 8 tab-separated columns:

```
reference_sequence_name source type start end score strand phase}
```

An optional 9th column defines any tags (separated by semi-colons). Blixem supports the following GFF tags. (Additional tags can be supplied but may be ignored.)

**Target**	(required for alignments)
**Gap**	(required for gapped alignments)
**ID**	(required for parent features)
**Name**	(required for transcripts and SNPs)

**Parent**	(required for child features)

In addition, Blixem supports the following custom tags.

**percentId**	(only applicable to alignments; populates the %ID column)
**sequence**	(only applicable to alignments; supplies thesequence data)
**variant_sequence**	(only applicable to variations; supplies the variation data)
**url**	(only used by variations; GFF3 special characters must be escaped)

Transcripts

Note that exons should have a Parent transcript defined, and the Name tag should be set in the parent rather than the child exons. Note that Blixem *will* recognise exons that do not have a Parent tag if they have a Name tag instead, but they may not get grouped correctly with other exons from the same transcript.

Typically, one defines the parent transcript, the exons, and the CDS regions; Blixem will then calculate the missing components (in this case, the UTR regions and the introns). Blixem will recognise other combinations of inputs, and will always calculate the missing components as long as enough information is provided.

Variations

SNPs, insertions and deletions are supported, as well as combined variations. One may use the generic 'sequence_alteration' type for these but it is good practice to use more specific types such as 'SNP' or 'deletion' where applicable.

Sample GFF file

A sample GFF file may look like this ('...' denotes that text has been omitted).

```
##gff-version 3
##sequence-region chr4-04 44144 154265
chr4-04 EST_Human nucleotide_match 79195 79311 95.000000 - . Target=DA692754\
.1 287 403 +;percentID=90.6;sequence=GATCTGGC...
chr4-04 EST_Human nucleotide_match 79195 79323 121.000000 + . Target=AI09510\
3.1 326 454 +;percentID=96.9;sequence=TTTAAATT...
chr4-04 ensembl_variation deletion 80798 80799 . + . Name=rs60725655;url=htt\
p%3A%2F%2Fwww.ensembl.org%2FHomo_sapiens%2FVariation%2FSummary%3Fv%3Drs60725\
655;variant_sequence=AA/-;
chr4-04 ensembl_variation sequence_alteration 80799 80799 . + . Name=rs57681\
246;url=http%3A%2F%2Fwww.ensembl.org%2FHomo_sapiens%2FVariation%2FSummary%3F\
v%3Drs57681246;variant_sequence=A/-/C;
chr4-04 ensembl_variation SNP 81040 81040 . + . Name=rs2352935;url=http%3A%2\
F%2Fwww.ensembl.org%2FHomo_sapiens%2FVariation%2FSummary%3Fv%3Drs2352935;var\
iant_sequence=T/C;
chr4-04 ensembl_variation insertion 82229 82230 . + . Name=rs35105663;url=ht\
tp%3A%2F%2Fwww.ensembl.org%2FHomo_sapiens%2FVariation%2FSummary%3Fv%3Drs3510\
5663;variant_sequence=-/G;
chr4-04 Augustus mRNA 119534 119941 . - . ID=transcript21;Name=AUGUSTUS00000\
051712
```

```
chr4-04 Augustus exon 119534 119941 . - . Parent=transcript21
chr4-04 Augustus CDS 119534 119941 . - 0 Parent=transcript21
```

FASTA file

A FASTA file has a header line that starts with '>'. We use a custom FASTA header format that contains the sequence name followed by the start and end coordinates, separated by spaces. Note that the FASTA sequence range may be different to the GFF file range.
The next line contains the start of the sequence data. The sequence data can be on a single line or separated by newlines; it is usually separated by newlines every 50 characters to aid readability.

```
>chr4-04 44144 154265
tcttgtttctgtaggagaggccatctccatcagctataaccaaaaaaaaa
acaaaaaactcctctttttgacaagtttgtaaagcctgtccatctgggtc
tataataatcctccaggccctatgccactcctctttattcagccagttca
...
```

Combined GFF and FASTA file

```
##gff-version 3
##sequence-region chr4-04 44144 154265
chr4-04_210623-364887 EST_Human nucleotide_match 79195 79311 95.000000 - . \
Target=DA692754.1 287 403 +;percentID=90.6
chr4-04_210623-364887 EST_Human nucleotide_match 79195 79323 121.000000 + .\
 Target=AI095103.1 326 454 +;percentID=96.9
...
##FASTA
>chr4-04 44144 154265
tcttgtttctgtaggagaggccatctccatcagctataaccaaaaaaaaa
acaaaaaactcctctttttgacaagtttgtaaagcctgtccatctgggtc
tataataatcctccaggccctatgccactcctctttattcagccagttca
...
```

Configuration file

Blixem supports ".ini-style" configuration files which are used to specify user options and to tell Blixem how to handle particular types of data. Blixem can accept config files by one or both of the following methods:

- A default config file called .blixemrc located in the user's home directory.
- A file passed on the command-line using the -c argument. The contents of this file will take priority if there are any clashes with the default file.

The default config file is generally used for display settings that are set from the Settings dialog. Blixem saves display settings to this file on exit, so it will be created the first time Blixem exits if it does not already exist. You can also edit this file by hand or add system settings to it such as the fetch methods if you wish.

The command-line method is useful when Blixem is called as part of a pipeline, because it allows the calling program to set specific config options (commonly the data-handling properties).

Program defaults

Defaults for the program can be specified in the [blixem] stanza. The properties that can be set are described below.

```
[blixem]
link-features-by-name=false
squash-linked-features=true
squash-identical-features=false
bulk-fetch = none
user-fetch = internal
stylesfile = ~/.ZMap/styles.ini
```

link-features-by-name
If true, features with the same name are considered to have the same parent, e.g. exons and introns with the same name are part of the same transcript, or matches with the same name are from the same match sequence.

squash-linked-features
If true, features that are linked under the same parent are squashed onto the same line when 'squash matches' is on.

squash-identical-features
If true, matches that are identical are squashed onto the same line when 'squash matches' is on.

bulk-fetch
This specifies the default method to use when batch-fetching sequences on start-up. Its value must be one of the fetch methods specified in the fetch method stanzas. The results of the fetch are parsed by Blixem. The bulk-fetch method can be overriden for specific data types (see the Data types section).

A comma-separated list of fetch methods can be specified if alternative fetch methods should be used if the first fetch fails for some reason. Each fetch method is tried in turn, in the order listed, until all sequences have been successfully fetched or we run out of methods to try.

user-fetch
This specifies the default method to use when the user interactively fetches a sequence from within Blixem, i.e. by double-clicking on a sequence. Its value must be one of the fetch methods specified in the fetch method stanzas. The results of the fetch are displayed to the user. The user-fetch method can be overridden for specific data types (see the Data types section).

A comma-separated list of fetch methods can be specified if alternative fetch methods should be used if the first fetch fails for some reason. Each fetch method is tried in turn, in the order listed, until the sequence has been successfully fetched or we run out of methods to try.

stylesfile
This specifies an ini-type file which is used to specify the colours that should be used for features in Blixem's transcript view. The file should contain one or more source stanzas followed by one or more key=value pairs, i.e.

```
[<source>]
<key>=<value>
...
```

`<key>` can be one of:

`colours`: default colours

`transcript-cds-colours`: used to specify a different colour for CDS sections

`<value>` is a semi-colon separated list of fill and line colours of the format

`<normal|selected> <fill|border> <colour>`

`<colour>` can be in any of the forms accepted by XParseColor; these include name for a colour from rgb.txt, such as DarkSlateGray, or a hex specification such as `#305050`.

Example

```
colours=normal border #0000af ; selected border #0000af ; normal fill white ;\
selected fill #ffddcc ;
transcript-cds-colours=normal border #0000af ; selected border #0000af ; norm\
al fill white ; selected fill #ffddcc ;
```

Note that selection colors will be calculated automatically if they are not specified (a darker shade of the default color will be used when the feature is selected).

Fetch methods

These stanzas define custom methods for fetching sequence data. Each fetch method must specify the `fetch-mode` key, which determines what type of fetch to perform. Other keys depend on the fetch mode. Valid fetch modes and their required keys are:

- `socket: node, port, command, args`
- `http: url, port, cookie-jar, request`
- `command: command, args`
- `sqlite: location, query`
- `www: url, request (user-fetch only; opens browser)`
- `internal: (user-fetch only; displays stored sequence)`
- `none: none`

In addition, the following keywords are required for bulk-fetch methods:

- `separator`: Specifies the separator between multiple sequence names when they are compiled into a list.
- `output`: Defines the output format and can be one of the following:
  - `raw`: raw sequence data; each sequence separated by a new line
  - `fasta`: FASTA format
  - `embl`: EMBL format
  - `list`: A list of named columns is returned
  - `gff`: GFF format for re-parsing

The following optional keywords can also be included for any fetch method:

- `errors`: Specifies a list of known error messages. This is used by Blixem to determine whether an error occurred even if the fetch program executed successfully. The value should be a comma-separated list of the expected error message text, e.g. `error="no match","Not authorized"`

The `request` and args values can include the following substitution symbols, which will be populated by blixem at run time. Use `%%` to represent a normal `%` character.

- `%p`: program name
- `%h`: host name
- `%u`: user name
- `%m`: match sequence name(s)
- `%r`: reference sequence name
- `%s`: start coord of feature on reference sequence
- `%e`: end coord of feature on reference sequence
- `%d`: dataset
- `%S`: feature source
- `%f`: file name (specified in the file tag in the GFF or in the Source stanza)
- `%(<key>)`: where `<key>` is any named key in the Source stanza

```
[pfetch-socket]
fetch-mode=socket
node=pfetch.sanger.ac.uk
port=22400
command=pfetch
args=--client=%p_%h_%u -q -C -F %m
errors="no match"","Not authorized"
separator=" "
output=embl

[pfetch-http]
fetch-mode=http
url=http://www.sanger.ac.uk:80/cgi-bin/otter/65/pfetch
request=request=%m
port=80}
cookie-jar=/nfs/users/nfs_g/gb10/.otter/ns_cookie_jar
errors="no match","Not authorized"
separator=" "
output=fasta

[www-fetch]
```

```
fetch-mode=www
url=http://www.sanger.ac.uk/cgi-bin/otter/65/pfetch
request=request=-F \%m

[variation-fetch]
fetch-mode=www
url=http://www.ensembl.org/Homo_sapiens/Variation/Summary
request=v=%m

[bam-fetch]
fetch-mode=command
command=bam_get
args=-file=http://hgdownload-test.cse.ucsc.edu/goldenPath/hg19/encodeDCC/wgEncode\
CshlLongRnaSeq/releaseLatest/%f
-chr_prefix=chr -gff_feature_source=%S -chr=%r -start=%s -end=%e
-dataset=%d
output=gff

[internal]
fetch-mode=internal

[none]
fetch-mode=none
```

Data types

You can override Blixem defaults by specifying a data-type for specific features. Data-types can be specified by a source mapping using the `[source-data-types]` stanza, or by using the custom `dataType` tag in the GFF input file. Possible key-value pairs are the same as for the Program defaults.

```
[dna-match]
link-features-by-name=true
bulk-fetch=pfetch-socket-embl,pfetch-socket-fasta
user-fetch=pfetch-http-embl,pfetch-http-fasta,internal

[protein-match]
link-features-by-name=true
bulk-fetch=pfetch-socket-fasta
user-fetch=pfetch-socket-embl,pfetch-socket-fasta,internal

[ensembl-variation]
user-fetch=variation-fetch
```

Source mapping

This stanza allows you to map a source to a particular data type. The keys should be valid sources that appear in the GFF file ,and the values must be stanzas specified in the data-type stanzas.

```
[source-data-types]
```

```
EST_Human=dna-match
EST_Mouse=dna-match
EST_Pig=dna-match
EST_Other=dna-match
SwissProt=protein-match
TrEMBL=protein-match
ensembl_variation=ensembl-variation
```

Sources

These stanzas allow you to set additional information on a per-source basis. You can substitute any value specified here using the `%(<key>)` substitution format, e.g. in the following example you could include the filename by using the substitution variable `%(file)`.

```
[Tier2_HepG2_cytosol_longPolyA_rep2]
file=wgEncodeCshlLongRnaSeqHepg2CytosolPapAlnRep2.bam
```

User settings

The following stanzas are used to specify display settings via the config file, that is, settings that the user can change via the Settings dialog in Blixem. These are saved to the default config file (`~/.blixemrc`) when Blixem exits so settings are persistent between Blixem sessions.

user-settings
This stanza is used to specify display options that Blixem will use on start-up. These are currently all true/false values which should be given 1 for true or 0 for false, except for num-unaligned-bases which takes an integer value.

```
[user-settings]
highlight-diffs=0
highlight-variations=1
show-variations-track=1
show-unaligned=0
show-unaligned-selected-seq=0
limit-unaligned=0
show-polya-site=0
show-poly-site-selected-seq=1
show-poly-sig=0
show-polya-sig-selected-seq=1
show-splice-sites=0
num-unaligned-bases=5
squash-matches=0
```

column-widths
This stanza is used to specify column widths that Blixem will use on start-up. It can also be used to hide a column by specifying a width of zero. Column names should be exactly as they appear in the column headers in Blixem, and are case-sensitive. Widths are specified in pixels.

```
[column-widths]
Name=120
```

```
Source=85
Organism=25
Gene Name=0
Tissue Type=0
Strain=0
Group=0
Score=0
\%Id=45
Start=
End=80
```

summary-columns
This stanza is used to specify which columns are included in the sequence summary details, which are shown in the feedback area when you mouse-over a sequence. Note that per-match columns such as Score, Start, End, etc. cannot be included so will be ignored if included in this stanza.

```
[summary-columns]
Name=true
Source=true
Organism=true
Gene Name=true
Tissue Type=true
Strain=true
Description=true
```

Colour key file

A ".ini"-style key file can be supplied via the --styles-file argument in order to tell Blixem what colour to draw certain features in, e.g.

```
[EST_Human]
fill_color=#ff0000
line_color=#bb0000
```

The group name (in square brackets) denotes a source, and the colours will apply to any features from the GFF file with the same source name. As many groups as required can be defined. Any features whose source does not have a group in the key file will use default colours.

The key-value pairs give the identifier of the colour and the colour string in hexadecimal format ("`#RRGGBB`"). Valid colour identifiers recognized by Blixem are:

```
fill_color
line_color
fill_color_selected
line_color_selected
fill_color_utr
line_color_utr
fill_color_utr_selected
line_color_utr_selected
```

Only `fill_color` and `line_color` are mandatory; the selection colors will be calculated automatically if not specified explicitly (a darker shade of the default color will be used when the feature is selected). For transcripts, the fill_color/line_color/etc items are used for CDS regions and different colors can be specified for UTR regions using fill_color_utr, line_color_utr etc.

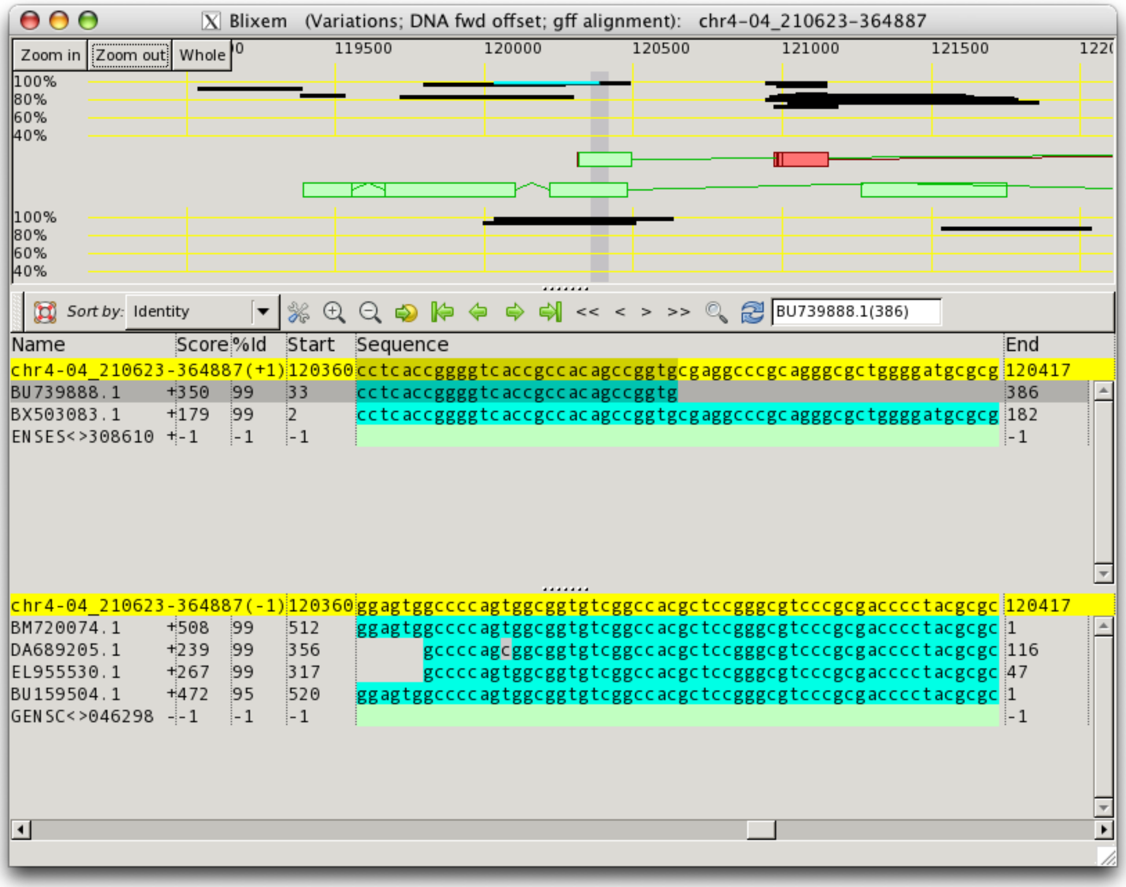

Figure 1: Nucleotide mode. There are two panes in the detail-view, one for each strand. The active strand is shown at the top. The active strand can be changed by hitting the 'Toggle' button or the 't' shortcut key.

The Blixem Window

The Blixem window consists of two main sections: an overview section called the "big picture", and a detail section showing the actual sequence data. These sections are separated by a splitter bar, so you can maximise the space for the area you are interested in. You can also hide sections of the window using the 'View' menu.

Blixem can show sequences in nucleotide or protein mode.

Active Strand

The "active" reference sequence strand in Blixem controls the orientation of the display – coordinates are shown increasing from left-to-right for the forward strand and decreasing for the reverse strand. The active strand is always shown at the top – i.e. the top grid and top transcript view in the big picture and the top pane in the detail view.

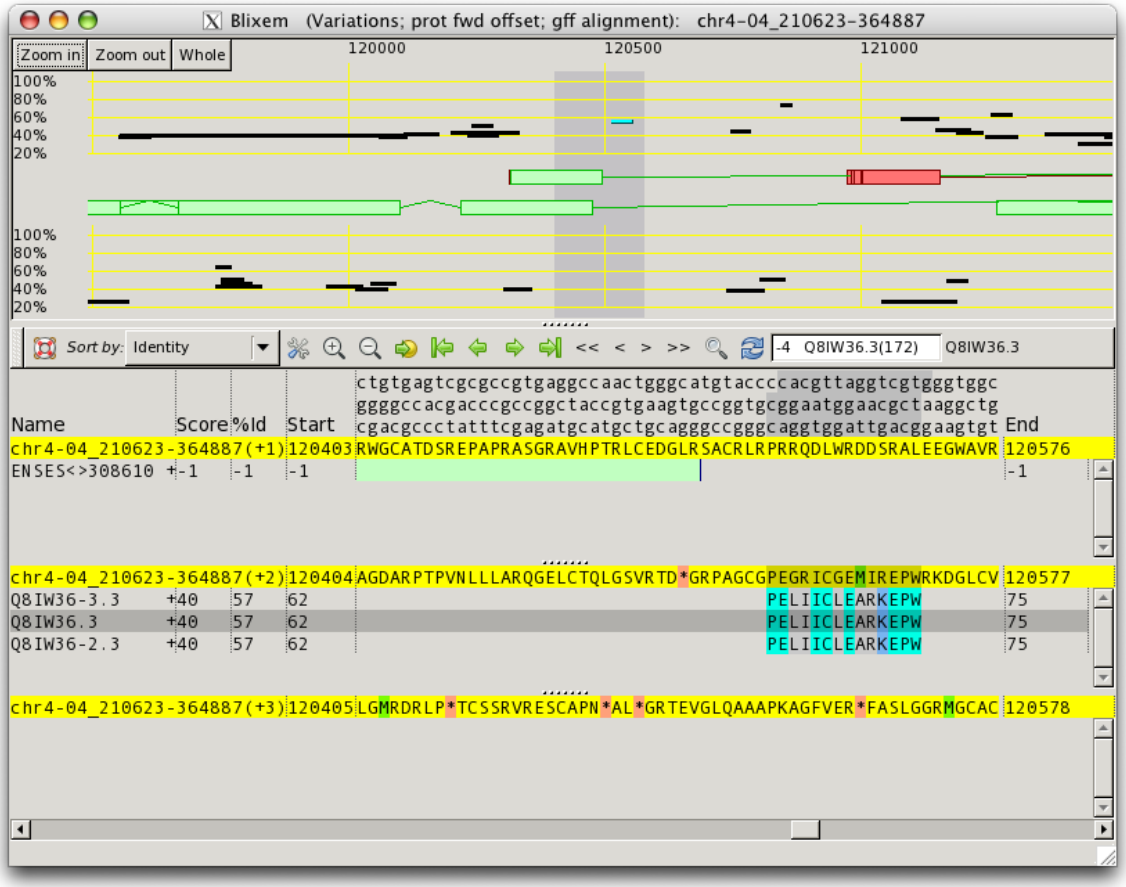

Figure 2: Protein mode. There are three panes in the detail-view; one for each reading frame of the active strand. The other strand can be activated by hitting the 'Toggle' button or the 't' shortcut key.

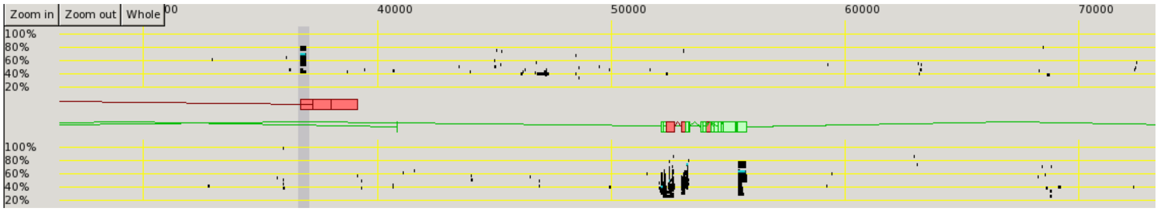

Figure 3: The Big Picture section

In protein mode, only the active strand is shown in the detail view. One must toggle the strand to view the other strand.

Toggle which strand is active by:

- pressing the ‘Toggle’ button 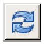 on the toolbar; or
- pressing the ‘t’ key.

By default, Blixem assumes that the reference sequence passed to it is the forward strand, unless otherwise specified by the ‘--reverse-strand’ command line argument.

Big Picture

The ‘Big Picture’ section shows an overview of the reference sequence. The reference sequence coordinates are shown along the top. You can zoom in to view a shorter range by using the 'Zoom in' button at the top left of the screen. Use 'Zoom out' or 'Whole' to zoom out – 'Whole' zooms out to view the full length of the reference sequence.

The big picture consists of two grids showing the alignments for each strand, and two sections between these grids showing the transcripts for each strand. The grids have a scale on the left-hand side showing the percent-ID, and alignments are plotted against this scale. The scale and extents of the grids can both be edited - see the Grid properties section in the Settings dialog.

The active strand alignments and transcripts are shown at the top and the other strand at the bottom. The direction of the coordinates is determined by the active strand. The active strand can be toggled using the 't' shortcut key or the 'Toggle strand' button on the toolbar.

Red shaded areas in the big picture indicate assembly gaps (gaps in the reference sequence). Assembly gaps are represented by dashes in the FASTA input file.

Bumping the transcript view

By default, exons and introns for the same strand are drawn overlapping each other. They can be expanded (or 'bumped') by pressing the 'b' shortcut key or by enabling the relevant option in the View dialog (see Hiding sections of the window).

Detail View

The ‘Detail View’ shows the actual sequence data for the match sequences. Match sequences are lined up underneath the relevant section of reference sequence, and individual bases are highlighted in different colours to indicate how well they match.

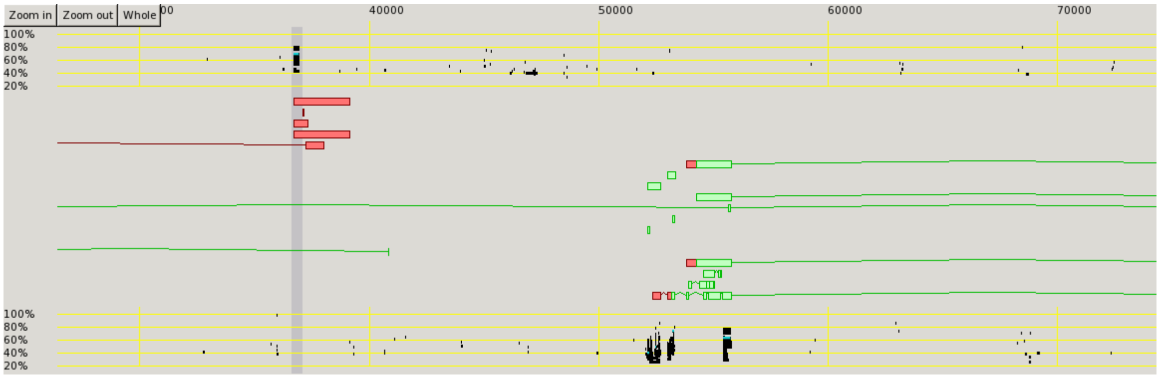

Figure 4: Expanded transcript view

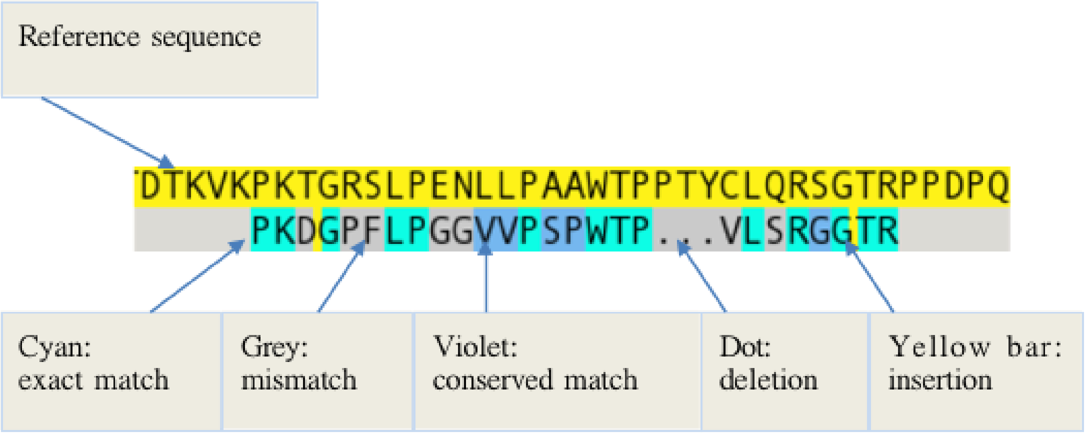

Figure 5: Alignment colour key

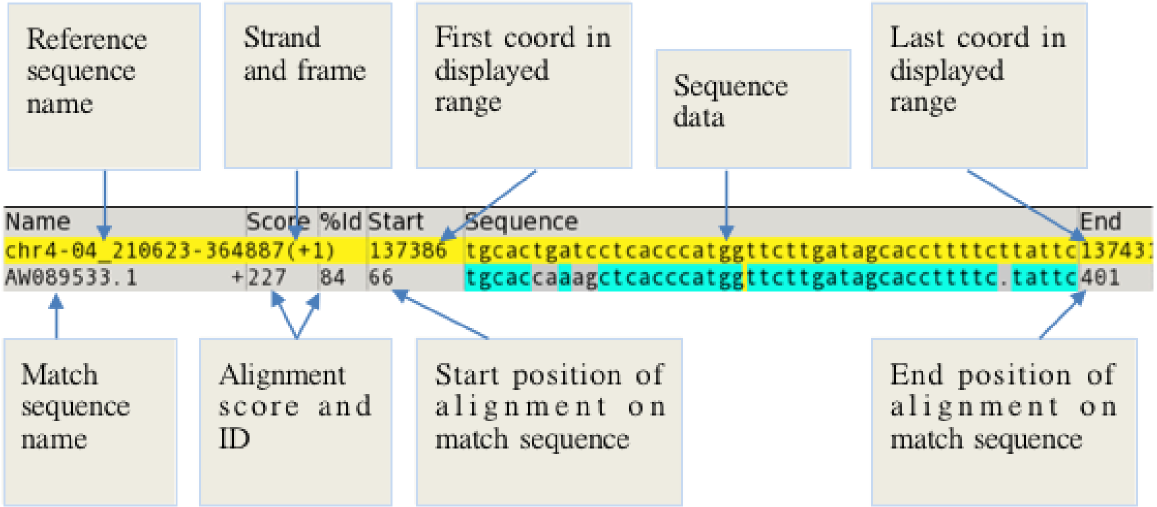

Figure 6: Alignment list details

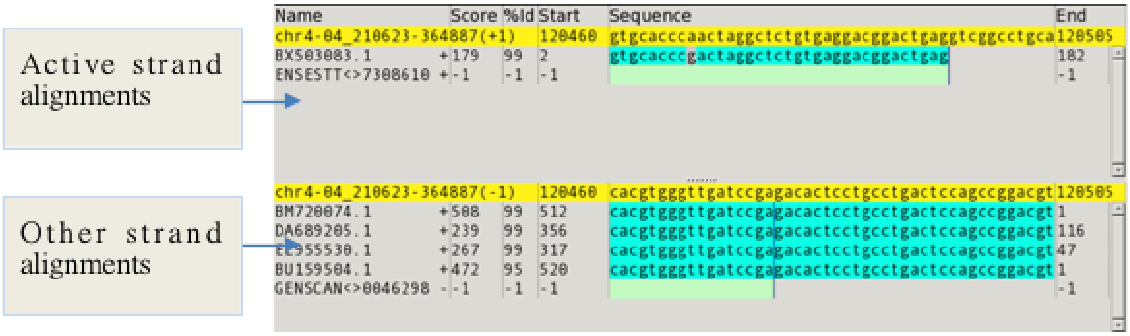

Figure 7: Alignment lists: nucleotide mode

Match colours

Alignment lists

There are separate lists of alignments for each strand and reading frame of the reference sequence. Each list has a yellow header bar containing the reference sequence. At the left, the yellow bar shows the reference sequence name and which strand/frame it is, e.g. (+1) means forward strand, reading frame 1; (-2) means reverse strand, reading frame 2.

Nucleotide mode

There are two sections to the detail view in nucleotide mode: one for each strand. The active strand is shown at the top and defines the coordinate direction (increasing if the forward strand is active, decreasing if the reverse is active).

Protein mode

There are three sections in the detail view in protein mode: one for each of the three reading frames for the active strand. Only the active strand is shown. To view the other strand, toggle the display using the 'Toggle strand' button or the 't' shortcut key.

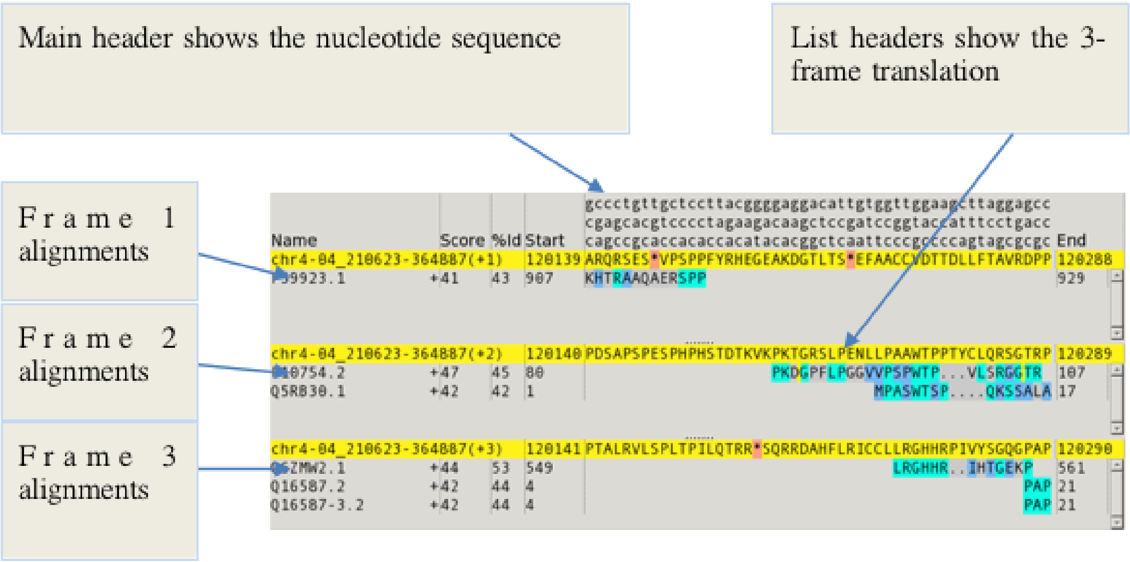

Figure 8: Alignment lists: protein mode

In protein mode, the yellow header bars show the translated reference sequence for that reading frame. STOP and MET codons in the reference sequence are highlighted in red and green. There is also an additional header section at the top showing the nucleotide sequence.

In the nucleotide-sequence header, codons are read from top-to-bottom and then left-to-right, starting at row 1 for frame 1, row 2 for frame 2 etc. Middle-clicking on a coordinate will highlight the three nucleotides for the selected codon and the currently-active reading frame (by default, frame 1). Left-clicking in an alignment list sets the active reading frame.

Exons

Exons are displayed as solid-colour blocks in the detail-view, coloured green for CDS, red for UTR. Vertical blue lines are drawn at the start and end of the blocks so that it is easy to see whether alignments line up with the exon boundaries.

In protein mode, an exon may not start or end exactly at a codon boundary. A "partial" or "split" codon like this is indicated in the detail-view by cross-hatch highlighting, and by drawing a dotted blue line rather than a solid line. (Note that dotted lines may be obscured by solid lines at the same position.)

The true boundary for split codons would really be either a third or two-thirds of the way through the character width, but Blixem does not draw boundaries through the middle of characters to avoid too cluttered a display.

Coverage view

The coverage view shows a plot of how many alignments there are at each coordinate along the reference sequence. It can give an indication of where the regions of interest are.

The coverage view can be shown/hidden by ticking/unticking the 'Show coverage view' check box on the View dialog (which can be accessed from the right-click menu or by hitting the 'v' shortcut key).

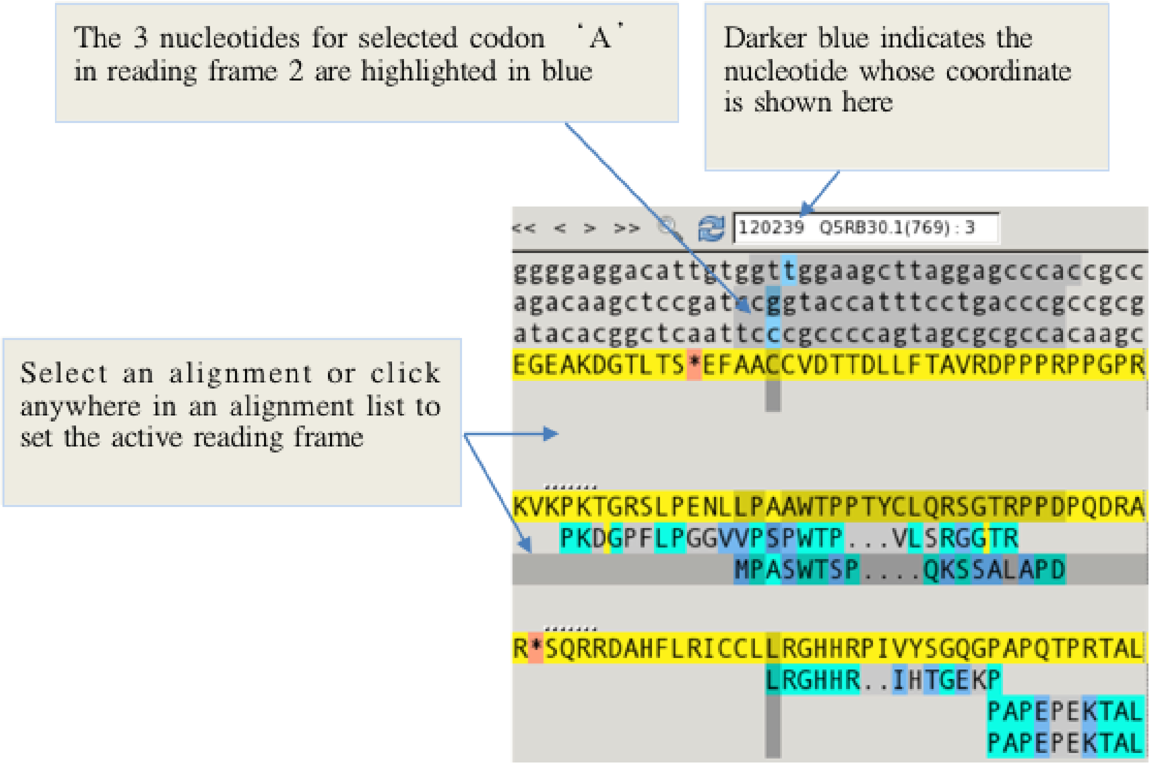

Figure 9: Selected reading frame and codon

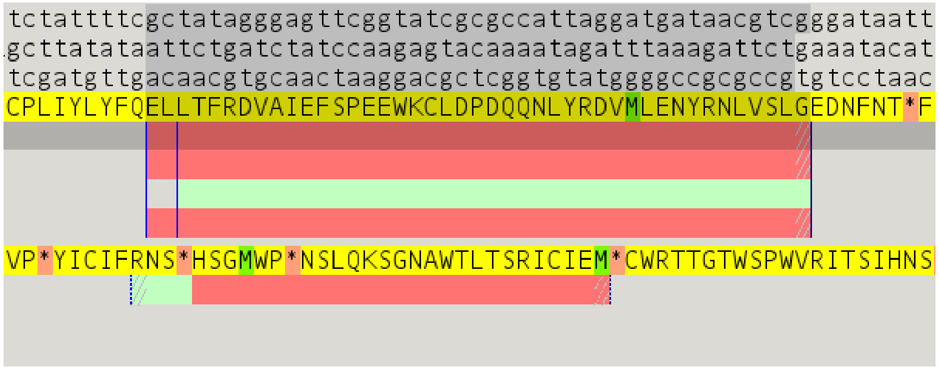

Figure 10: Exons in the detail-view. Split codons are indicated with cross-hatching, e.g. the last codon in the selected exon is a split codon because it does not include all three bases for that codon, as you can see from the highlighting in the DNA header.

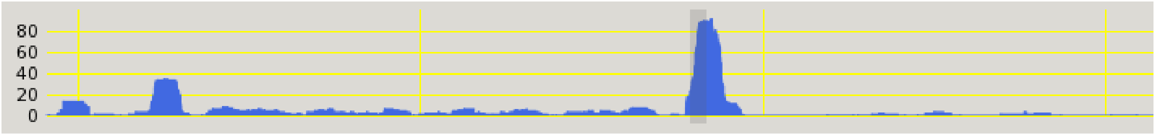

Figure 11: Coverage view

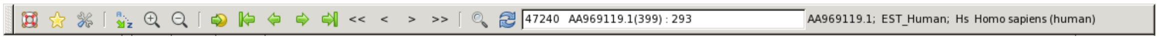

Figure 12: Detail-view toolbar

The scale of the coverage view is the same as that of the big-picture and it can be navigated in the same manner, i.e.

- use the horizontal scroll-bar or middle-click to scroll; and
- use the zoom buttons at the top or the Ctrl-=/Ctrl-- keys to zoom.

The toolbar

The detail-view toolbar contains the following functions. Note that the Help and Settings buttons are included in the detail-view toolbar even though they apply to Blixem as a whole.

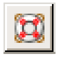	**Help**:	Show help about how to use Blixem
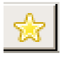	**About:**	Show program information
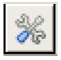	**Settings**:	Show the Settings dialog
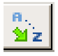	**Sort**:	Show the Sort dialog
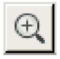	**Zoom in**:	Increase the font size in the detail-view
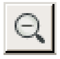	**Zoom out**:	Decrease the font size in the detail-view
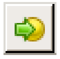	**Go to**:	Go to a particular coordinate
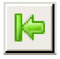	**First match**:	Go to the first coordinate of the first alignment[1]
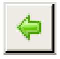	**Previous match**:	Go to the start of the current alignment or the end of the previous alignment[1]
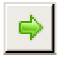	**Next match**:	Go to the end of the current alignment or the start of the next alignment[1]
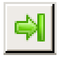	**Last match**:	Go to the end of the last alignment[1]
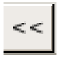	**Back one page**:	Scroll the detail-view range to the left by one page
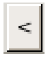	**Back one index**:	Scroll the detail-view range to the left by one base

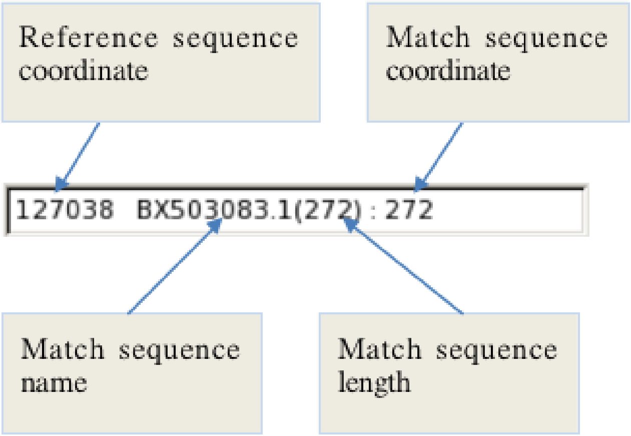

Figure 13: Feedback box

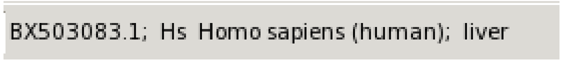

Figure 14: Moused-over item feedback area

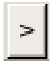	**Forward one index**:	Scroll the detail-view range to the right by one base
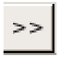	**Forward one page**:	Scroll the detail-view range to the right by one page
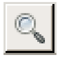	**Find**:	Scrolls to the start of the first alignment from that sequence if any are found.
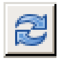	**Toggle strand**:	Toggle which strand is the active strand

Feedback box
The feedback box contains information about the currently selected sequence and/or coordinate, if either is selected. Click on a row in the detail-view to select a sequence. Middle-click on a base in the detail-view to select that coordinate. Text in the feedback box can be selected and copied.

Moused-over item feedback area
The area to the right of the toolbar contains information about the currently moused-over item (e.g. a match sequence in the alignment list or a variation in the variations track). For a match sequence, this information includes the sequence name and optional data such as organism and tissue type that can be parsed from EMBL files. To load optional data, see the Settings dialog. Note that the optional data may be incomplete due to the inconsistent information available from the EMBL files.

The main menu

Right-click anywhere in the Blixem window to pop up the main menu.
The options are:

[1] Acts only on selected sequences, if there is currently a selection; if no sequences are currently selected, then this operation acts on all sequences.

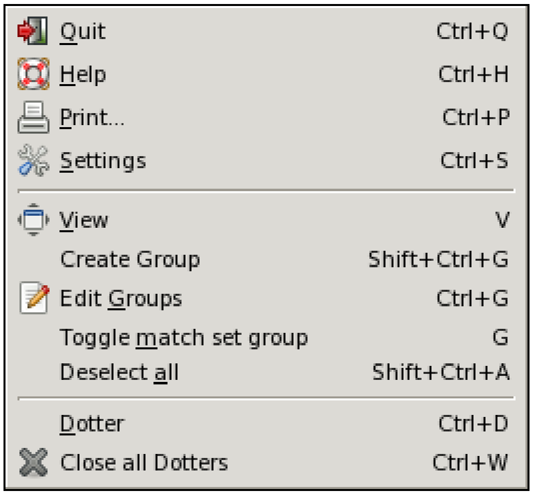

Figure 15: Main menu

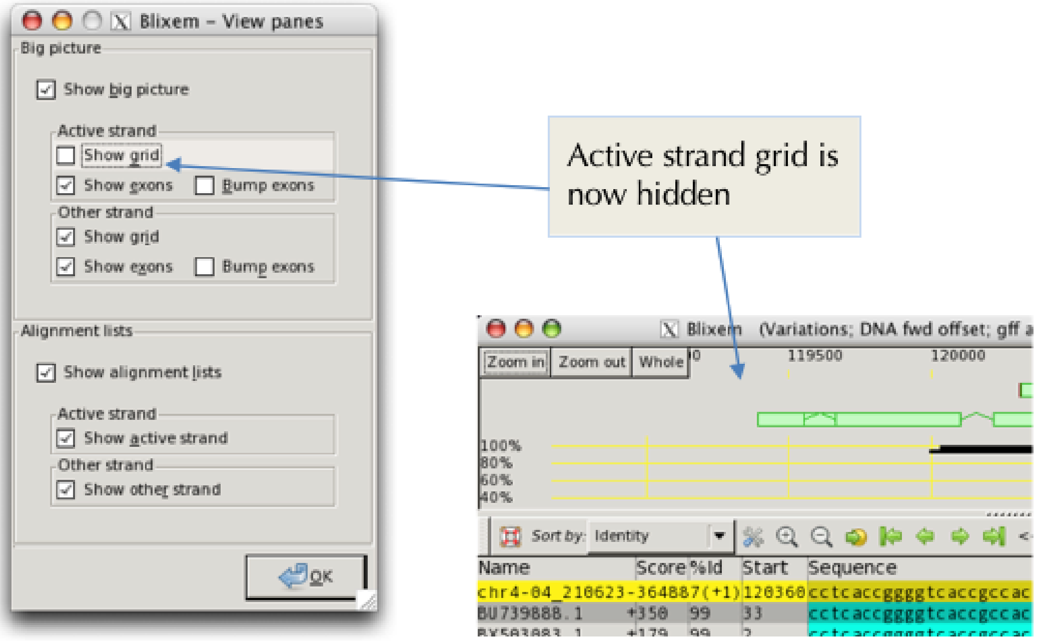

Figure 16: The View dialog

**Quit**	*Ctrl-Q*	Close Blixem and any spawned processes
**Help**	*Ctrl-H*	Display the user help
**Print**	*Ctrl-P*	Printing options
**Settings**	*Ctrl-S*	Edit settings
**View**	*v*	Show/hide parts of the display
**Create Group**	*Shift-Ctrl-G*	Create a group of sequences
**Edit Groups**	*Ctrl-G*	Edit properties for groups
**Toggle match set group**	*G*	Toggle the special “match set” group on and off. This is a quick way of creating a group from the current selection buffer, which should contain match sequence names.
**Deselect all**	*Shift-Ctrl-A*	Deselect all sequences
**Dotter**	*Ctrl-D*	Run Dotter on the currently selected sequence
**Close all Dotters**		Close all Dotters that have been opened from this Blixem

Hiding sections of the window

Use to ‘View’ dialog to show/hide sections of the window.

1. Right-click and select the View option, or hit the ’v’ shortcut key.

2. Toggle check marks on or off to show/hide sections.

Alternatively, use the following keyboard shortcuts to toggle visibility of a component:

**1**	Hide top pane in detail view
**2**	Hide second pane in detail view
**3**	Hide third pane in detail view (protein mode only)
**Ctrl-1**	Hide top grid in big picture (active strand)
**Ctrl-2**	Hide bottom grid in big picture (other strand)
**Shift-Ctrl-1**	Hide top exon view (active strand)
**Shift-Ctrl-2**	Hide bottom exon view (other strand)

Operation

Navigation

Scrolling

**Middle-click/double-click and then drag in big picture**	Jump to a particular region. Dragging moves the highlight box.
**Click on the highlight box and drag**	Move the highlight box.
**Middle-click/drag in detail view**	Select a base. Releasing the mouse button scrolls the display to centre on the selected base (hold down Ctrl to avoid scrolling.)
**Click a feature in the big picture**	Selects that feature and scrolls the detail-view vertically so that it is visible (if it is in the current detail view range).
**Horizontal scrollbar**	Scroll the detail-view range.
**Vertical scrollbars**	Scroll up/down in the detail view or the big picture.
**Horizontal mouse-wheel**	Scroll the detail-view range (if your mouse has a horizontal scroll-wheel).
**Vertical mouse-wheel**	Scroll up/down the currently moused-over alignment list in the detail view, or the big picture.

**Ctrl-left**<br>**Ctrl-right**	Scroll to the start/end of the previous/next match (limited to currently-selected sequences, if any are selected; includes all sequences otherwise).
**Home**<br>**End**	Scroll to the start/end of the display.
**Ctrl-Home**<br>**Ctrl-End**	Scroll to the start/end of the currently-selected alignments (or to the first/last alignment if none are selected).
**',' (comma)**<br>**'.' (full-stop)**	Scroll the detail-view range one nucleotide to the left/right.
**Ctrl-,**<br>**Ctrl-.**	Scroll the detail-view range one page to the left/right.
**Go-to button or 'p' key**	Scroll to a specific coordinate position.

Zooming

**= - keys and** 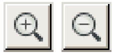	Zoom in/out of the detail-view
**Ctrl-= or Ctrl-- keys and** 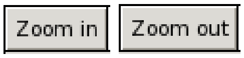	Zoom in/out of the big-picture
**Shift-Ctrl-- and** 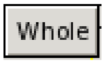	Zoom the big picture out to view the full length of the reference sequence.

Selections

Selecting sequences

- You can select a sequence by clicking on its row in the alignment list. Selected sequences are highlighted in cyan in the big picture.
- You can select a sequence by clicking on it in the big picture.
- The name of the sequence you selected is displayed in the feedback box on the toolbar. If there are multiple alignments for the same sequence, all of them will be selected.
- You can select multiple sequences by holding down the Ctrl or Shift keys while selecting rows.
- You can deselect a single sequence by Ctrl-clicking on its row.
- You can deselect all sequences by right-clicking and selecting 'Deselect all', or with the Shift-Ctrl-A keyboard shortcut.

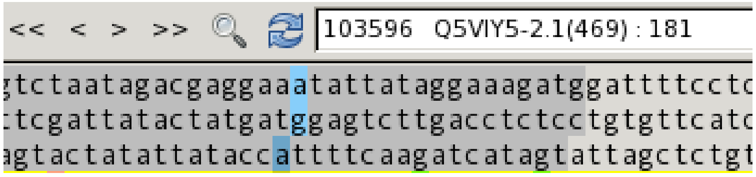

Figure 17: The 3 nucleotides for the currently-selected amino acid in reading-frame 3. Selected nucleotide 103596 is shaded in darker blue.

- You can move the selection up/down a row using the up/down arrow keys.

Selecting coordinates

- You can select a nucleotide/peptide by middle-clicking on it in the detail view. This selects the entire column at that index, and the coordinate number on the reference sequence is shown in the feedback box. (The coordinate on the match sequence is also shown if a match sequence is selected.)
- By default the display will centre on the selected base when you middle click. To select a base without scrolling, hold down Ctrl when you middle click.
- For protein matches, when a peptide is selected, the three nucleotides for that peptide (for the active reading frame) are highlighted in the header in blue. (The active reading frame is whichever alignment list currently has the focus - click in a different list to change the reading frame.) Darker blue highlighting indicates the specific nucleotide that is currently selected (i.e. whose coordinate is displayed in the feedback box).
- You can move the selection to the previous/next index using the left and right arrow keys.
- In protein mode, you can move the selected nucleotide by a single base (rather than an entire codon) holding Shift while using the left and right arrow keys.
- You can move the selection to the start/end of the previous/next match by holding Ctrl while using the left and right arrow keys (limited to just the selected sequences if any are selected; includes all sequences otherwise).

Finding sequences

The Find dialog allows the user to search for sequences by name. Press the Find 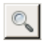 button on the toolbar or hit the 'Ctrl-F' shortcut key to open the Find dialog.

There are three search modes:

- Text search: Search for match sequences by name (or another column from the 'Search column' drop-down box). The wild-card '*' means any number (or zero) of any character and '?' means 1 character (which can be any character). Any sequences whose relevant column data matches the search string will be selected and the display will scroll to the start of the selection.
- List search: the same as text-search, but you can enter multiple search strings by placing them on separate lines in the text box.

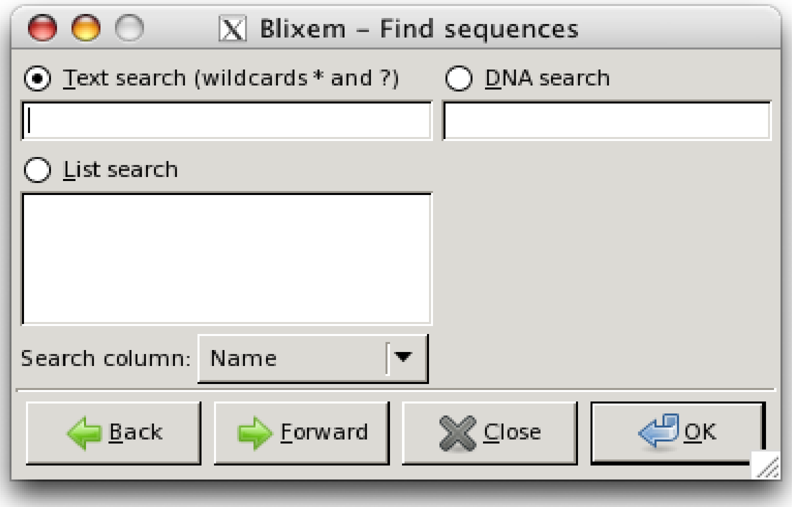

Figure 18: Find dialog

- DNA search: This searches for a given sub-sequence of nucleotides in the reference sequence. If the sub-sequence is found, the display will scroll to the start of the sub-sequence and the first base in the sub-sequence will be selected.

Enter your search text in the appropriate box and click the OK button to perform the search. By default, Blixem will start searching from the beginning of the reference sequence range. To start the search from the current position instead, click the Forward or Back button instead of OK. This will start searching from the currently-selected base, if there is one selected; if not, it will start from the beginning of the current detail-view display range when searching forwards or from the end of the display range if searching backwards.

Repeat a Find
After clicking OK on the Find dialog, press F3 to repeat the search in a forwards direction or Shift-F3 to repeat in a backwards direction. Alternatively, if you had selected the Forward or Back button in the Find dialog then click the Forward or Back buttons again to jump to the next result in that direction.

Copy and paste

- When sequence(s) are selected, their names are copied to the *selection buffer* and can be pasted to another program by middle-clicking in that program.
- Sequence names can be pasted from the selection buffer into Blixem by hitting the 'f' keyboard shortcut. If the selection buffer contains valid sequence names, those sequences will be selected and the display will jump to the start of the selection.
- Sequence names can also be pasted from the selection buffer into text boxes in dialog boxes such as the Groups dialog or Find dialog.
- To copy sequence name(s) to the *default clipboard*, select the sequence(s) and hit Ctrl-C. Sequence names can then be pasted into other applications using Ctrl-V.

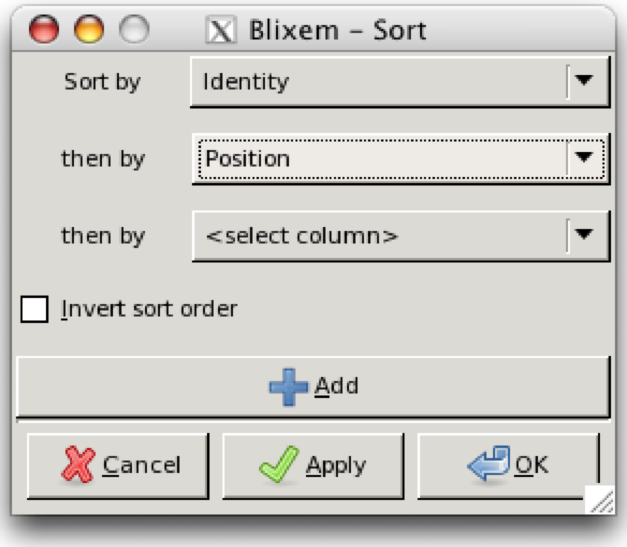

Figure 19: Sort dialog

- The default clipboard can be pasted into Blixem using Ctrl-V. If the clipboard contains valid sequence names, those sequences will be selected and the display will jump to the start of the selection.
- Note that text from the feedback box and some text labels (e.g. the reference sequence start/end coords) can be copied to the selection buffer by selecting the required text with the mouse (or copied to the default clipboard by selecting it and then hitting 'Ctrl-C').
- Text can be pasted from the default clipboard into text entry boxes on dialogs such as the Groups or Find dialog by using Ctrl-V.

Sorting alignments

- Click the sort button on the toolbar to open the Sort dialog.
- Select the column you wish to sort by from the top drop-down box on the dialog.
- You may optionally sort by further columns. You can sort by as many columns as you wish by adding further drop-down boxes using the Add button.
- The default sort order may be ascending or descending depending on what makes most sense for the selected column: e.g. sorting by position is *ascending* by default, but sorting by score or ID is *descending*.
- To get the inverse of the default sort order, select the 'Invert sort order' option on the Sort dialog.
- Alignments can also be sorted by group. Alignments that are part of a group will then be listed first (before any that are not in a group), and ordered according to the group's order number. See the Groups section for more details.

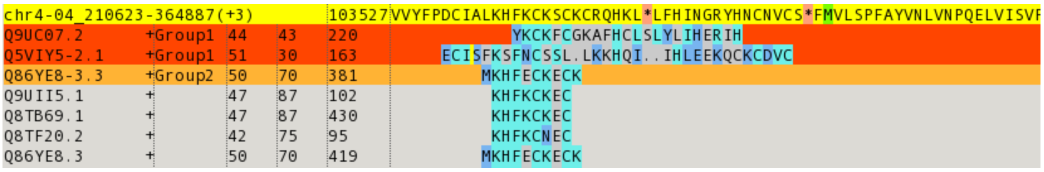

Figure 20: Alignment list sorted by group

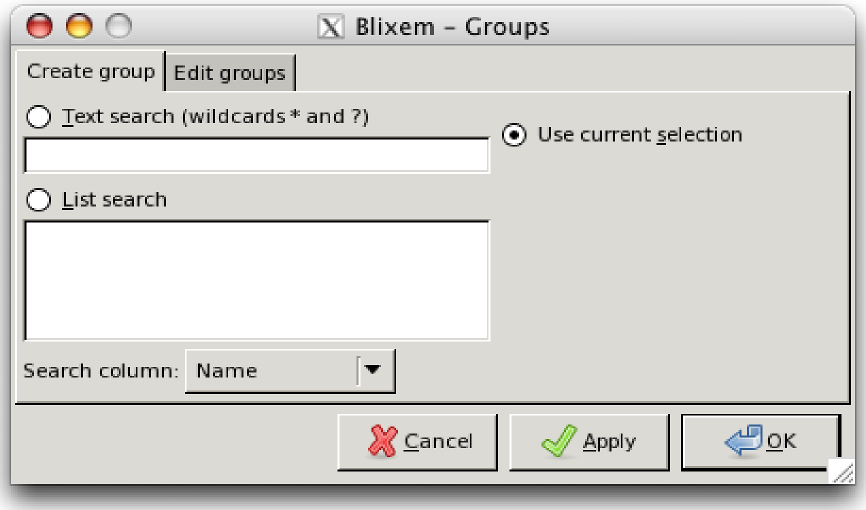

Figure 21: Groups dialog - create group

Fetching sequences

- Double-click a row to fetch a match sequence's EMBL file.

Grouping sequences

Alignments can be grouped together so that they can be sorted/highlighted/hidden etc.

Creating a group from a selection

- Select the sequences you wish to include in the group by left-clicking their rows in the detail view. Multiple rows can be selected by holding the Ctrl or Shift keys while clicking.
- Right-click and select 'Create Group', or use the Shift-Ctrl-G shortcut key. (Note that Ctrl-G will also shortcut to here if no groups currently exist.)
- Ensure that the 'From selection' radio button is selected, and click 'OK' or 'Apply'. If you click 'Apply', you will be shown the group you just created so that you can edit it. If you click 'OK' the group will be created with the default properties.

Creating a group from a search

- Right-click and select 'Create Group', or use the Shift-Ctrl-G shortcut key. (Or Ctrl-G if no groups currently exist.)

- Select the 'Text search' or 'List search' radio button and enter some text to search for.
- Select the column that you wish to search in the drop-down box at the bottom.
- Click OK or Apply.

Notes

- 'List search' allows you to enter multiple search strings; place each string on a separate line.
- You can use the following wild-cards in the search text: an asterisk (*) represents any number of characters; a question mark (?) represents any single character.
- You can paste text into the search boxes from the selection buffer by middle-clicking or from the clipboard using Ctrl-V.
- You may paste sequence names directly from another compatible program (e.g. ZMap): click on the feature in ZMap and then middle-click in the text box on the Groups dialog. (Grouping in Blixem works on the sequence name alone, so the feature coords output by ZMap will be ignored.)

Creating a temporary 'match-set' group from the current selection

- You can quickly create a group from a current selection (e.g. selected features in ZMap or just the current selection in Blixem) using the 'Toggle match set' option.
- To create a match-set group, select the required items and then select 'Toggle match set' from the right-click menu in Blixem, or hit the 'g' shortcut key.
- To clear the match-set group, choose the 'Toggle match set' option again, or hit the 'g' shortcut key again.
- While it is enabled (i.e. toggled on), the match-set group can be edited like any other group, via the 'Edit Groups' dialog. Any settings you change (e.g. highlight colour) will be saved even if the match-set group is toggled off and then on again.
- If you delete the match-set group using the 'Edit Groups' dialog, all of its settings will be lost; you will get the default settings again the next time you enable the match-set group. To avoid this, disable it by toggling it off using the 'Toggle match set' menu option (or 'g' shortcut key) rather than by deleting it in the Groups dialog.

Editing groups

To edit a group, right-click and select 'Edit Groups', or use the Ctrl-G shortcut key.
You can change the following properties for a group. Click on Apply or OK to apply the changes.

**Name**	You can specify a more meaningful name to help you identify the group.
**Hide**	Tick this box to hide the alignments in the alignment lists.
**Highlight**	Tick this box to highlight the alignments.

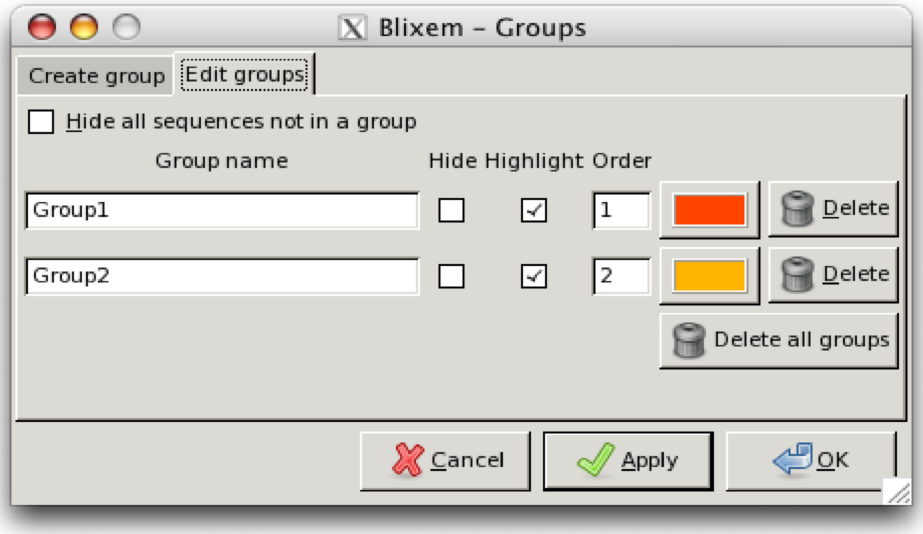

Figure 22: Groups dialog – edit groups

Colour The colour the group will be highlighted in, if 'Highlight' is enabled. The default colour for all groups is orange, so you may wish to change this if you want different groups to be highlighted in different colours.

Order When sorting by Group, alignments in a group with a lower order number will appear before those with a higher order number (or vice versa if sort order is inverted). Alignments in a group will appear before alignments that are not in a group.

You can also hide all sequences that are not part of a group by ticking the 'Hide all sequences not in a group' option. This is a quick way of filtering sequences to show only those that you are interested in; any sequences that are not part of a group will be hidden. Note that any sequences in a hidden group will also still be hidden.

To delete a group, click one of the following buttons. This will have an immediate effect (i.e. you don't have to click 'Apply').

- To delete a single group, click on the 'Delete' button next to the group you wish to delete.
- To delete all groups, click on the 'Delete all groups' button.

Running dotter

- To start Dotter from within Blixem, or to edit the parameters for running Dotter, right-click and select 'Dotter' or use the Ctrl-D keyboard shortcut. The Dotter dialog will pop up.

- Select the sequence you wish to run Dotter on before or after opening the dialog. The selected sequence name will be shown at the top of the dialog.

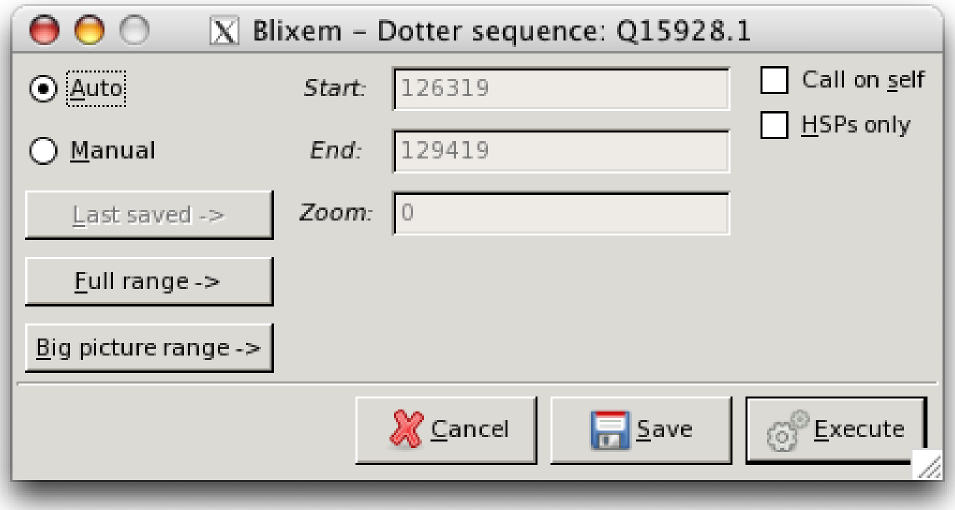

Figure 23: Dotter dialog

- Alternatively, if you just wish to edit the settings, you do not need to select a sequence.
- To run Dotter with the default (automatic) parameters, just hit RETURN, or click the 'Execute' button. In this mode, Dotter will run on the range that is currently visible in the Big Picture section.
- To enter custom parameters, select the 'Manual' radio button and enter the values in the 'Start' and 'End' boxes.
- To save the parameters without running Dotter, click Save and then Cancel'.
- To save the parameters and run Dotter, click 'Execute'.
- To revert to the last-saved manual parameters, click the 'Last saved' button.
- To revert back to automatic parameters, click the 'Auto' radio button. The coordinates in the Start and End box will be recalculated for the currently-selected sequence.

Reference sequence versus itself

To run Dotter on the reference sequence versus itself, select the 'Call on self' tick box in the Dotter dialog and then click 'Execute'. This can be useful to analyse internal repeats etc. (see the Dotter manual for more information).

Dotter HSPs only

This starts Dotter in HSP (High-Scoring Pair) mode (see the Dotter manual).

Settings

The settings menu can be accessed by right-clicking and selecting Settings, or by the shortcut Ctrl-S.

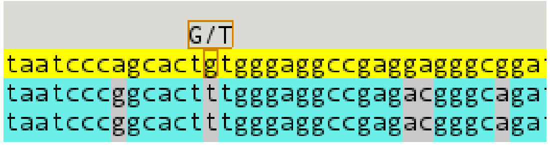

Figure 24: Variations track

Options

Highlight variations

When this option is enabled, bases in the reference sequence that have know variations (such as SNPs, insertions, deletions etc., loaded from the GFF file) are highlighted in the reference sequence (nucleotide) header.

If the 'Show variations track' sub-option is also enabled, then an additional line is shown above the nucleotide header showing the alternative bases for each variation. Note that the Variations track can be quickly enabled or disabled by double-clicking the nucleotide header.

You can double-click a variation to open its URL.

Show polyA tails

When this option is enabled, polyA tails are shown and highlighted in the alignment lists and polyA signals are highlighted in the reference sequence (nucleotide) header. If the sub-option 'Selected sequences only' is enabled, polyA tails will only be shown for the currently selected sequences.

Annotated polyA sites and signals form the input features file are also highlighted in the reference sequence. Mouse-over an annotated site/signal to see its details.

Show Unaligned Sequence

When this option is enabled, any additional, unaligned portions of the match sequences are displayed at the start and end of the alignments. If the 'Limit to' sub-option is also enabled, you can specify the maximum number of additional bases to display. If the 'Selected sequences only' sub-option is enabled, only the currently selected sequence(s) will display unaligned portions of sequence.

Show Colinearity Lines

When this option is enabled, colinearity lines are displayed between alignment blocks of the same sequence. The lines are green to indicate perfectly colinear, orange to indicate imperfectly colinear, and red to indicate not colinear. If the 'Selected sequences only' sub-option is enabled, colinearity lines are only displayed for the currently selected sequence(s) in the detail view, otherwise they are shown for all sequences in the detail view. Note that colinearity lines are only displayed for the selected sequence(s) in the big picture regardless of this setting, to save cluttering the screen.

Show Splice Sites

When this option is enabled, splice sites are highlighted in the reference sequence (nucleotide) header for the currently-selected sequence(s). The two bases from the adjacent introns are highlighted in green if they are canonical or red if they are non-canonical.

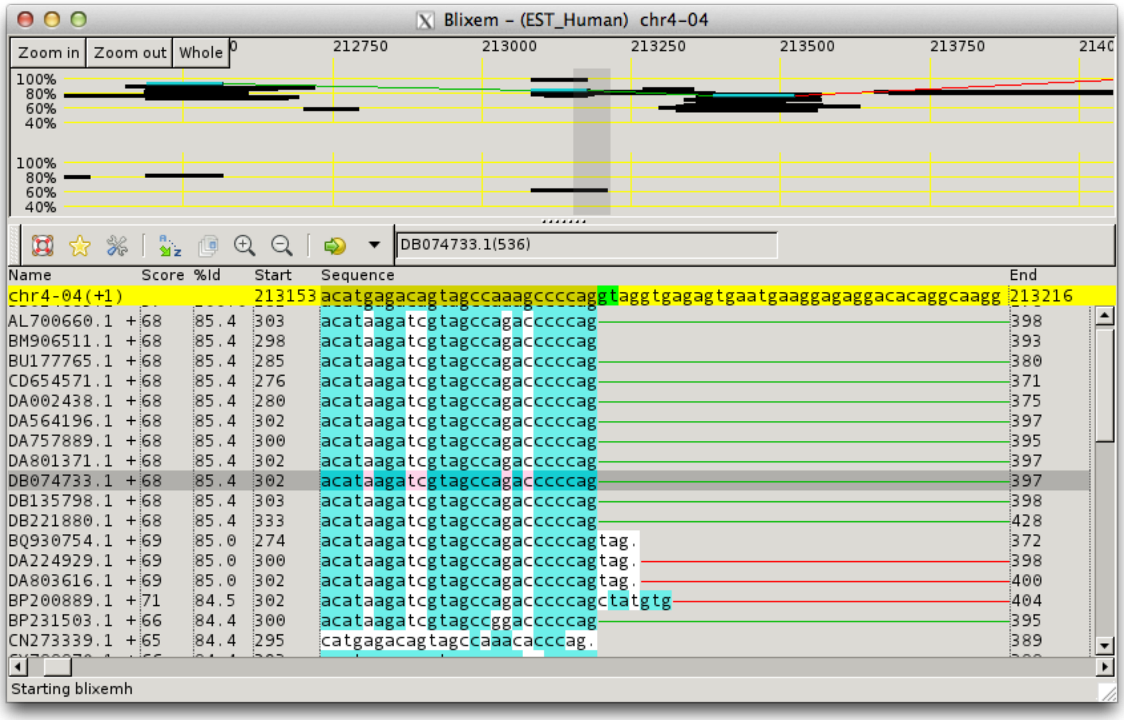

Figure 25: Colinearity lines between alignment blocks and highlighted splice-sites in the reference sequence

Highlight Differences

When this option is enabled, matching bases are blanked out and mismatches are highlighted, making it easier to see where alignments differ from the reference sequence.

Squash Matches

This groups multiple alignments from the same sequence together into the same row in the detail view, rather than showing them on separate rows.

Display

Use print colours

Select this option to make Blixem use grey-scale colours, suitable for printing.

Font

Allows you to change the font that is used to display alignments in the detail-view. Note that you must select a monospace font; otherwise matches will not be shown aligned correctly. Blixem will warn you if the font you have selected is not monospace.

%ID per cell

Use this to change the vertical scale of the big picture grid; a smaller value means the grid will be more spaced out, a larger value means the grid will be more compact.

Max %ID

Defines the maximum cut-off value for the %ID scale in the big picture grid.

Min %ID

Defines the minimum cut-off value for the %ID scale in the big picture grid.

Depth per cell

Use this to change the vertical scale of the grid for the Coverage View (see the View menu to turn on the Coverage View); a smaller value means the grid will be more spaced out, a larger value means the grid will be more compact.

Columns

Load optional data

Click this button to load optional data from EMBL entries (an `optional-fetch` method must be set up in the blixem config file). Note that this operation can take a long time if there are many sequences. The button will be greyed out once optional data has been loaded.

Column settings

Tick/un-tick the check-marks to show/hide individual columns and to include/hide column details in the mouse-over box. Adjust the column width by entering the new width in the text box in pixels. Note that if you enter a zero width then the column will be hidden, regardless of whether the check-mark is ticked or not. Greyed-out columns are optional-data columns, and will only become available once optional data has been loaded.

Colours

Change any of Blixem's custom display colours, such as the colour aligned bases are shown in or the colour stop codons are highlighted in etc. There are four colours for each item:

- Normal: this is the standard display colour;
- Normal (selected): this is the colour used when the item is selected (if applicable). Typically one would use a slightly darker or lighter shade of the Normal colour for this, so that the item does not look radically different when it is selected;
- Print: this is the standard colour used when the 'Use print colours' option is enabled;
- Print (selected): this is the colour used when 'Use print colours' is enabled and the item is selected.

Key

In the detail view, the following colours and symbols have the following meanings:

Alignment list header	Yellow background	Reference sequence
Alignment list	Cyan background	Identical residues
Alignment list	Violet background	Conserved residues
Alignment list	Grey background	Mismatch
Alignment list	'.' with grey background	Deletion
Alignment list	Purple vertical line	Insertion
Alignment list	Thin blue vertical line	Boundary of an exon
Alignment list	Thin horizontal line	Colinearity lines between alignment blocks: green for perfect colinearity, orange for imperfect colinearity, red if not colinear
Nucleotide header (protein mode)	Sky-blue background	The three nucleotides for the currently-selected codon; darker blue indicates the nucleotide whose coordinate is displayed in the feedback box
Alignment list header (protein mode)	Pale red background	STOP codon
Alignment list header (protein mode)	Green background	MET codon

Keyboard shortcuts

**Ctrl-Q**	Quit
**Ctrl-H**	Help
**Ctrl-P**	Print
**Ctrl-S**	Edit settings
**V**	Show/hide sections of the display
**Shift-Ctrl-G**	Create group
**Ctrl-G**	Edit groups (or create a group if none currently exist)
**Ctrl-A**	Select all sequences in the current list
**Shift-Ctrl-A**	Deselect all sequences
**Ctrl-D**	Dotter
**Left-arrow**	Move coordinate section one index to the left[2]
**Right-arrow**	Move coordinate section one index to the right[2]
**Shift-Left**	Same as Left, but in protein mode it scrolls by a single nucleotide
**Shift-Right**	Same as Right, but in protein mode it scrolls by a single nucleotide
**Ctrl-Left**	Scroll to the start/end of the previous alignment[3]
**Ctrl-Right**	Scroll to the start/end of the next alignment[3]
**Up-arrow**	Move row selection up
**Down-arrow**	Move row selection down
**Home**	Scroll to the start of the display
**End**	Scroll to the end of the display
**Ctrl-Home**	Scroll to the start of the first alignment[3]
**Ctrl-End**	Scroll to the end of the last alignment[3]
=	Zoom in detail view
**-**	Zoom out detail view
**Ctrl-=**	Zoom in big picture
**Ctrl--**	Zoom out big picture
**Shift-Ctrl--**	Zoom out big picture to view the whole reference sequence
**,**	Scroll left one coordinate
**.**	Scroll right one coordinate
**P**	Go to position
**T**	Toggle the active strand
**G**	Toggle the 'match set' Group
**1**	Toggles visibility of the 1st alignment list
**2**	Toggles visibility of the 2nd alignment list
**3**	Toggles visibility of the 3rd alignment list (protein mode only)

**Ctrl-1**	Toggles visibility of the 1st big picture grid
**Ctrl-2**	Toggles visibility of the 2nd big picture grid
**Shift-Ctrl-1**	Toggles visibility of the 1st exon view
**Shift-Ctrl-2**	Toggles visibility of the 2nd exon view

[2] Only applicable if a coordinate is currently selected; middle-click a coordinate to select it.
[3] Limited to just the selected sequences, if any are selected; otherwise, acts on all sequences.
