# Supplementary material for: SeqTools: visual tools for manual analysis of sequence alignments
Source: BMC Res Notes. 2016 Jan 22;9:39. doi: 10.1186/s13104-016-1847-3 (PMC4724122; doi:10.1186/s13104-016-1847-3)
Supplement: Supplementary file 1 — 10.1186/s13104-016-1847-2 A tarball of the current production release of the SeqTools source code at the time of writing. [file 13104_2016_1847_MOESM1_ESM.gz › seqtools-4.32.1/doc/User_doc/blixem_quick_start.html]

Blixem - Quick Start


# Blixem - Quick Start

- What's new
- Overview
- Zooming
- Scrolling
- Selecting sequences
- Main menu
- Feedback bar
- Status bar
- Find
- Fetching sequences
- Copy and paste
- Sorting
- Groups
- Dotter
- Settings
- Colour key
- Keyboard shortcuts

## Overview

This page gives a quick-start guide to using Blixem. Other documentation is available here:

- What's new (revision history)
- Usage (command line options)
- User manual
- Outstanding issues

## Zooming

- Zoom in to the currently-selected region in the big picture using the +/- buttons in the top-left corner of the window, or using the Ctrl '=' and Ctrl '-' shortcut keys. The 'Whole' button zooms out to show the full length of the query sequence.
- Zoom in/out of the detail view using the +/- buttons on the toolbar, or using the '=' and '-' shortcut keys.

## Scrolling

- Middle-click in the big picture to scroll to an area.
- Middle-click in the detail view to centre on a base.
- Use the horizontal scrollbar at the bottom of the window to scroll the detail view.
- Use the mouse-wheel to scroll up/down in the list of alignments.
- Use the mouse-wheel to scroll the alignments left/right (if your mouse has a horizontal scroll-wheel).
- Ctrl-left and Ctrl-right arrow keys scroll to the start/end of the previous/next match (limited to currently-selected sequences, if any are selected).
- The Home/End keys scroll to the start/end of the display.
- Ctrl-Home and Ctrl-End scroll to the start/end of the currently-selected alignments (or to the first/last alignment if none are selected).
- The ',' (comma) and '.' (full-stop) keys scroll the display one nucleotide to the left/right.
- Pressing Ctrl and ',' (comma) or '.' (full-stop) scrolls the display one page to the left/right.
- You can scroll to a specific position using the Go-to button on the toolbar, or by pressing the 'p' shortcut key.

## Selections

- You can select a sequence by clicking on its row in the alignment list. Selected sequences are highlighted in cyan in the big picture.
- You can select a sequence by clicking on it in the big picture.
- The name of the sequence you selected is displayed in the feedback box on the toolbar. If there are multiple alignments for the same sequence, all of them will be selected.
- You can select multiple sequences by holding down the Ctrl or Shift keys while selecting rows.
- You can deselect a single sequence by Ctrl-clicking on its row.
- You can deselect all sequences by right-clicking and selecting 'Deselect all', or with the Shift-Ctrl-A keyboard shortcut.
- You can move the selection up/down a row using the up/down arrow keys.
- If the 'Show Splice Sites' option is on (see the 'Settings' dialog), splice sites will be highlighted on the reference sequence for the currently-selected alignment(s).

- You can select a nucleotide/peptide by middle-clicking on it in the detail view. This selects the entire column at that index, and the coordinate number on the query sequence is shown in the feedback box. (The coordinate on the subject sequence is also shown if a subject sequence is selected.)
- For protein matches, when a peptide is selected, the three nucleotides for that peptide (for the current reading frame) are highlighted in the header in blue. The current reading frame is whichever alignment list currently has the focus - click in a different list to change the reading frame. The darker highlighting indicates the specific nucleotide that is currently selected (i.e. whose coordinate is displayed in the feedback box).
- You can move the selection to the previous/next nucleotide using the left and right arrow keys.
- You can move the selection to the previous/next peptide by holding Shift while using the left and right arrow keys.
- You can move the selection to the start/end of the previous/next matchb by holding Ctrl while using the left and right arrow keys (limited to just the selected sequences if any are selected).
- To select a coordinate without the display re-centering on it, hold down Ctrl as you middle-click.

## Main menu

Right-click anywhere in the Blixem window to pop up the main menu. The menu options are:

- Quit: Quit Blixem.
- Help: Display this help.
- Print: Print the display
- Settings: Edit settings.
- View: Display the View dialog box. This allows you to show/hide parts of the display.
- Create Group: Create a group of sequences.
- Edit Groups: Edit properties for groups.
- Deselect all: Deselect all sequences.
- Dotter: Run Dotter and/or set Dotter parameters.
- Feature series selection tool: ???

## Feedback about current items

There are two feedback boxes on the toolbar that display information about current items:

- The first shows info about the currently-selected alignment and/or the currently-selected index.
- The second shows info about the currently moused-over alignment. As a minimum this will display the sequence name. It will also show additional data such as organism and tissue type if available - if not already loaded, this data can be loaded by clicking the 'Load optional data' button on the Settings dialog.

## Status bar

- The status bar at the bottom of the window shows all messages and warnings, including non-critical messages.
- Critical messages are also displayed in a popup dialog or message list.
- The user may switch between popup dialogs or the message list by selecting the 'Switch to scrolled message window' on the popup dialog or the 'Switch to popup messages' check box on the message list.

## Find sequences

- Click the find icon on the toolbar or press the Ctrl-F shortcut to open the find dialog.
- To search for sequences by name, use either the 'Sequence name search' or 'Sequence name list' search box. Enter the sequence name you wish to search for and hit 'OK', 'Forward' or 'Back'.
- To search for a string of nucleotides in the reference sequence, use the 'DNA search' box. Enter the string of nucleotides and hit 'OK', 'Forward' or 'Back'. Note that the search is only performed on the active strand: use the 'Toggle strand' button on the toolbar or hit the 't' shortcut key to toggle which strand is active.
- Using the 'OK' button will search for the first match in the entire Blixem range.
- Using the 'Forward' or 'Back' buttons will search for the next/previous match from the current position.
- After performing a find, you can repeat the search by hitting the F3 key (or Shift-F3 to search backwards).
- You can perform a fast find on sequence name(s) in the selection buffer by hitting the 'f' shortcut key.

## Fetch sequences

- Double-click a row to fetch a sequence.
- In the Settings dialog, click the 'Load optional data' button to fetch additional information such as organism and tissue-type. This data can be viewed by enabling the corresponding column and is also displayed in the feedback area of the toolbar when a sequence is moused-over.

## Copy and paste

- When sequence(s) are selected, their names are copied to the selection buffer and can be pasted by middle-clicking.
- To paste sequence names from the selection buffer, hit the 'f' keyboard shortcut. Blixem will select the sequences and jump to the start of the selection.
- To copy sequence name(s) to the default clipboard, select the sequence(s) and hit Ctrl-C. Sequence names can then be pasted into other applications using Ctrl-V.
- The clipboard text can also be pasted into Blixem using Ctrl-V. If the clipboard contains valid sequence names, those sequences will be selected and the display will jump to the start of the selection.
- Note that text from the feedback box and some text labels (e.g. the reference sequence start/end coords) can be copied by selecting it with the mouse and then hitting Ctrl-C.
- Text can be pasted into dialog box text entry boxes using Ctrl-V (or middle-clicking to paste from the selection buffer).

## Sorting

- The alignments can be sorted by selecting the column(s) you wish to sort by from the drop-down boxes on the Sort dialog. Open the dialog from the icon on the toolbar.
- You can reverse the sort order by selecting the 'Invert sort order' option on the Sort dialog. Note that some columns sort ascending by default (e.g. name, start, end) and some sort descending (score and ID). This option reverses that sort order.
- If you sort by one of the optional columns (e.g. organism or tissue-type) the sort will have no effect until the data for that column is loaded. Load the data by clicking the 'Load optional data' button in the 'Columns' section of the Settings dialog.
- You can place alignments in a group and then sort by group to keep specific sequences always on top. See the Groups section for more details.

## Groups

Alignments can be grouped together so that they can be sorted/highlighted/hidden etc.

### Creating a group from a selection:

- Select the sequences you wish to include in the group by left-clicking their rows in the detail view. Multiple rows can be selected by holding the Ctrl or Shift keys while clicking.
- Right-click and select 'Create Group', or use the Shift-Ctrl-G shortcut key. (Note that Ctrl-G will also shortcut to here if no groups currently exist.)
- Ensure that the 'From selection' radio button is selected, and click 'OK'.

### Creating a group from a sequence name:

- Right-click and select 'Create Group', or use the Shift-Ctrl-G shortcut key. (Or Ctrl-G if no groups currently exist.)
- Select the 'From name' radio button and enter the name of the sequence in the box below. You may use the following wildcards to search for sequences: '\*' for any number of characters; '?' for a single character. For example, searching for '\*X' will find all sequences whose name ends in 'X' (i.e. all exons).
- Click 'OK'.

### Creating a group from sequence name(s):

- Right-click and select 'Create Group', or use the Shift-Ctrl-G shortcut key. (Or Ctrl-G if no groups currently exist.)
- Select the 'From name(s)' radio button.
- Enter the sequence name(s) in the text box.
- You may use the following wildcards in a sequence name: '\*' for any number of characters; '?' for a single character. (For example, searching for '\*X' will find all sequences whose name ends in 'X', i.e. all exons).
- You may search for multiple sequence names by separating them with the following delimiters: newline, comma or semi-colon.
- You may paste sequence names directly from ZMap: click on the feature in ZMap and then middle-click in the text box on the Groups dialog. Grouping in Blixem works on the sequence name alone, so the feature coords will be ignored.
- Click 'OK'.

### Creating a temporary 'match-set' group from the current selection:

- You can quickly create a group from a current selection (e.g. selected features in ZMap) using the 'Toggle match set' option.
- To create a match-set group, select the required items (e.g. in ZMap) and then select 'Toggle match set' from the right-click menu in Blixem, or hit the 'g' shortcut key.
- To clear the match-set group, choose the 'Toggle match set' option again, or hit the 'g' shortcut key again.
- While it exists, the match-set group can be edited like any other group, via the 'Edit Groups' dialog.
- If you delete the match-set group from the 'Edit Groups' dialog, all settings (e.g. highlight color) will be lost. To maintain these settings, clear the group using the 'Toggle match set' menu option (or 'g' shortcut key) instead.

### Editing groups:

To edit a group, right-click and select 'Edit Groups', or use the Ctrl-G shortcut key. You can change the following properties for a group:

- Name: you can specify a more meaningful name to help identify the group.
- Hide: tick this box to hide the alignments in the alignment lists.
- Highlight: tick this box to highlight the alignments.
- Color: the color the group will be highlighted in, if 'Highlight' is enabled. The default color for all groups is red, so you may wish to change this if you want different groups to be highlighted in different colors.
- Order: when sorting by Group, alignments in a group with a lower order number will appear before those with a higher order number (or vice versa if sort order is inverted). Alignments in a group will appear before alignments that are not in a group.
- To delete a single group, click on the 'Delete' button next to the group you wish to delete.
- To delete all groups, click on the 'Delete all groups' button.

## Dotter

- To start Dotter, or to edit the Dotter parameters, right-click and select 'Dotter' or use the Ctrl-D keyboard shortcut. The Dotter settings dialog will pop up.
- To run Dotter with the default (automatic) parameters, just hit RETURN, or click the 'Execute' button.
- To enter manual parameters, click the 'Manual' radio button and enter the values in the 'Start' and 'End' boxes.
- To revert to the last-saved manual parameters, click the 'Last saved' button.
- To dotter against a transcript's sequence, click the Transcript radio button. You will need to select a transcript to dotter against. Hold down Ctrl to select a transcript without deselecting the match you want to dotter.
- To change the match sequence to dotter against, select the Match Sequence tab. You can dotter the currently selected match (default), any ad-hoc sequence in raw or FASTA format, or you can dotter the reference sequence vs itself.
- To save the parameters without running Dotter, click Save and then Cancel'.
- To save the parameters and run Dotter, click 'Execute'.
- To close all Dotters that have been started from the current Blixem, right-click in the Blixem window and choose 'Close all Dotters'.
- Note that all Dotters opened from within Blixem will be closed when Blixem exits.

## Settings

The settings menu can be accessed by right-clicking and selecting Settings, or by the shortcut Ctrl-S. Soome settings (those in the 'Features' and 'Display options' panes on the dialog, as well as the column widths) are saved to the file ".blixemrc" in your home directory when you quit Blixem, and will be restored the next time Blixem is run. To revert to the default settings, just delete the ".blixemrc" file.

- **Highlight variations**:Tick this box to highlight bases in the reference sequence that have SNPs, insertions, deletions etc. Hover over a highlighted base to see details of the variation in the feedback area.
- **Show variations track**:The variations track shows the details of the variations. Double-click on a variation to open its URL. The Highlight-Variations option must be enabled to use this option. A shortcut to quickly enable both options is to double-click the reference sequence. Double click the reference sequence again to hide the variations track.
- **PolyA tails**:Tick this option to show polyA tails in the alignment lists. By default polyA tails are only shown for the currently-selected sequence(s). To show them for all sequences, un-tick the 'Selected sequences only' box. When a polA tail is displayed, polyA signals are also highlighted in the reference sequence.
- **Show Unaligned Sequence**:Show any additional unaligned parts of the match sequence at the start/end of the alignment. Specify the number of additional bases to show in 'Limit to... bases', or untick this option to show all of the unaligned sequence. Tick the 'Selected sequences only' option to only show unaligned sequence for the currently selected sequence(s). Note that this option does not work with the 'Squash Matches' option, so it will not do anything if 'Squash Matches' is on.
- **Show Splice Sites**:Shows splice sites for the currently-selected alignment(s). Splice sites are highlighted on the reference sequence in green (for canonical) or red (for non-canonical). Blixem identifies GT-AG, GC-AG and AT-AC introns as canonical.
- **Highlight Differences**:When this option is set, matching bases are blanked out and mismatches are highlighted, making it easier to see where alignments differ from the reference sequence.
- **Squash Matches**:Group multiple alignments from the same sequence together into the same row in the detail view, rather than showing them on separate rows.
- **Columns**:Show/hide columns or edit the width of columns (in pixels). Click the 'Load optional data' button to load the data for the optional columns such as organism and tissue-type - then tick the check box to show this column in the Blixem window. The optional columns are greyed out if their data is not loaded. Once optional data is loaded you can also sort by it. Note that optional data is loaded on startup for DNA matches but not for protein matches, because the latter can be slow. The Load Optional Data button will be greyed out if optional data is already loaded.
- **Grid properties**:Set the maximum/minimum %ID values shown in the big picture. Expand or contract the grid scale by adjusting '%ID per cell'. The ID per cell can be set to as low as 0.1. Note that setting a low ID-per-cell can result in a large number of cells and hence a very large grid, and Blixem is not currently very clever about dealing with this.

## Color key

In the detail view, the following colors and symbols have the following meanings:

- Yellow: Query sequence
- Cyan: Identical match
- Blue: Similar match
- Grey: Mismatch
- **`.`**: Deletion
- | Purple bar: Insertion
- \_\_\_\_\_\_\_\_ Empty green box: Coding (CDS) exon
- \_\_\_\_\_\_\_\_ Empty red box: Non-coding (UTR) exon
- | Blue bar: Start boundary of an exon
- | Dark blue bar: End boundary of an exon
- Blue in DNA headerNucleotides of the selected codon. Darker blue indicates the specific nucleotide displayed in the feedback box.
- `*` STOPs are highlighted in red in the translation
- `M` METs are highlighted in green in the translation

## Keyboard shortcuts

- **Ctrl-Q** Quit
- **Ctrl-H** Help
- **Ctrl-P** Print
- **Ctrl-S** 'Settings' menu
- **Ctrl-F** Open the Find dialog
- **f** Perform a find on sequence name(s) in the current selection buffer
- **Ctrl-G** Edit groups (or create a group if none currently exist)
- **Shift-Ctrl-G** Create group
- **g** Toggle the 'match set' Group
- **Shift-Ctrl-A** Deselect all alignments
- **Escape** Deselect the currently-selected base index
- **Ctrl-D** Dotter
- **Left**/**right** arrow keys Move the currently-selected coordinate one place to the left/right
- **Shift-Left**/**right** Same as left/right arrow keys, but for proteins it scrolls by a single nucleotide, rather than an entire codon.
- **Ctrl-Left**/**right** Scroll to the start/end of the previous/next alignment (limited to just the selected sequences, if any are selected).
- **Home**/**End** Scroll to the start/end of the display.
- **Ctrl-Home**/**End** Scroll to the first/last alignment (limited to just the selected sequences, if any are selected).
- **,** (comma) scroll left one coordinate
- **.** (period) scroll right one coordinate
- **=** (equals) zoom in detail view
- **-** (subtract) zoom out detail view
- **Ctrl**-**=** zoom in big picture
- **Ctrl**-**-** zoom out big picture
- **Shift**-**Ctrl**-**-** zoom out big picture to whole width
- **V** 'View' menu (for toggling visibility)
- **1**, **2**, **3** These number keys toggle visibility of the 1st, 2nd (and 3rd, for protein matches) alignment list.
- **Ctrl**-**1**, **Ctrl**-**2** This toggles visibility of the 1st and 2nd big picture grid.
- **Shift**-**Ctrl**-**1**, **Shift**-**Ctrl**-**2** This toggles visibility of the 1st and 2nd exon views.
- **p** Go to position
- **t** Toggle the active strand
