# Supplementary material for: SeqTools: visual tools for manual analysis of sequence alignments
Source: BMC Res Notes. 2016 Jan 22;9:39. doi: 10.1186/s13104-016-1847-3 (PMC4724122; doi:10.1186/s13104-016-1847-3)
Supplement: Supplementary file 1 — 10.1186/s13104-016-1847-2 A tarball of the current production release of the SeqTools source code at the time of writing. [file 13104_2016_1847_MOESM1_ESM.gz › seqtools-4.32.1/doc/User_doc/Dotter_manual/Dotter_manual.pdf]

Dotter User Manual

Written by Gemma Barson
<gb10@sanger.ac.uk>

Wellcome Trust Sanger Institute
18 January 2011

Revision History

Revision	Date	Author
First revision (Dotter v4.1.5)	18/01/11	Gemma Barson
Updated for Dotter v4.1.9	10/02/11	Gemma Barson
Updated for Dotter v4.1.13	25/03/11	Gemma Barson
Updated for Dotter v4.1.14	04/04/11	Gemma Barson
Updated for Dotter v4.7	02/12/11	Gemma Barson
Updated for Dotter v4.27	17/04/14	Gemma Barson

Contents

Revision History 2

Introduction 4

Getting Started 4
- Running Dotter . . . 4
  - Sequence versus itself . . . 4
- Input files . . . 4
  - FASTA file . . . 6
  - GFF file . . . 6
  - Transcripts . . . 7
  - Sample GFF file . . . 7

The Dotter Windows 7
- The dot-plot window . . . 7
  - Cross-hair . . . 7
  - Zoom in with a child Dotter . . . 9
- The alignment tool . . . 9
  - Alignment tool menu . . . 10
  - Alignment tool shortcuts . . . 11
- Greyramp tool . . . 11

Main menu 11
- File menu . . . 11
- Edit menu . . . 13
- View menu . . . 14
- Help menu . . . 15

Settings 15
  - Zoom . . . 15
  - Horizontal range . . . 15
  - Vertical range . . . 15
  - Sliding window size . . . 16
  - Show break-lines . . . 16
  - Show break-lines . . . 16
  - Show horizontal sequence labels . . . 16
  - Show vertical sequence labels . . . 16

Keyboard shortcuts 17

Introduction

This manual explains how to configure, run and use Dotter. Dotter is a graphical dot-plot program for detailed comparison of two sequences. Every residue in one sequence is compared to every residue in the other sequence. The first sequence runs along the x-axis and the second sequence along the y-axis. In regions where the two sequences are similar to each other, a row of high scores will run diagonally across the dot matrix.

Pairwise scores are averaged over a sliding window to make the score matrix more intelligible. The averaged score matrix forms a three-dimensional landscape, with the two sequences in two dimensions and the height of the peaks in the third. This landscape is projected onto two dimensions using a grey-scale image - the darker grey of a peak, the higher the score is.

The contrast and threshold of the grey-scale image can be adjusted, and a tool is provided to examine the sequence alignment that the grey-scale image represents.

Dotter is maintained by the Wellcome Trust Sanger Institute and is available as part of the SeqTools package. The software can be downloaded from the Sanger Institute's website:
http://www.sanger.ac.uk/resources/software/seqtools

Getting Started

Running Dotter

As a minimum, Dotter takes the following required arguments:

```
dotter <horizontal_sequence> <vertical_sequence>
```

where <horizontal_sequence> and <vertical_sequence> are the path names of FASTA files containing the two input sequences. Dotter will assume that the sequences both start at coordinate 1 unless you use the -q and -s arguments to set an offset for the query (horizontal) and subject (vertical) sequences respectively.

Run ‘dotter‘ without any arguments to see further usage information.

Sequence versus itself

Dotter can be run on a sequence versus itself. This can be useful to analyse internal repeats. You can also look for overlaps between many sequences by making a dot-plot of all of the sequences versus themselves. To run Dotter on many sequences at once, concatenate the FASTA files for all of the sequences and then run Dotter on the concatenated sequence file against itself.

If you're comparing a sequence against itself, you'll notice that the main diagonal scores maximally, since it's the 100% perfect self-match. Partitioning break-lines will appear between the sequences if there were multiple sequences in the input file.

Input files

The sequence input files are in FASTA format. Comparisons are allowed between two nucleotide sequences, two protein sequences, or one nucleotide and one protein sequence – note that when

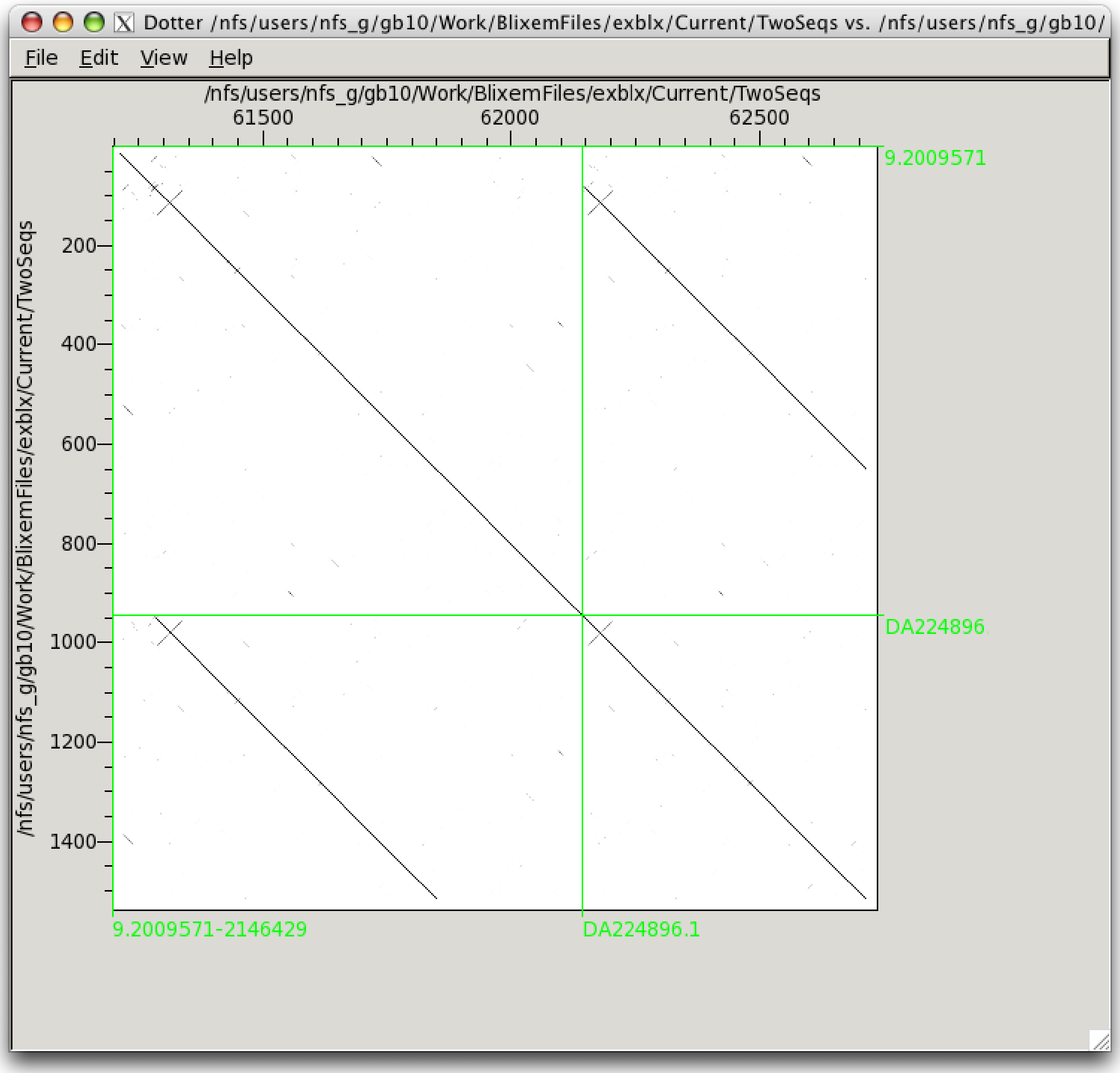

Figure 1: Multiple sequences vs themselves

comparing a nucleotide and a protein sequence, the nucleotide sequence must be passed first (i.e. as the horizontal sequence).

Additional features can be passed to Dotter in a GFF file using the -f argument. Relevant features include alignments, which can be viewed using Dotter's HSP mode, and transcripts, which are shown at the bottom of the Dotter window.

FASTA file

A FASTA file has a header line that starts with '>' and contains the sequence name. The next line contains the start of the sequence data. The sequence data can be on a single line or separated by newlines; it is usually separated by newlines every 50 characters to aid readability.

```
>chr4-04
tcttgtttctgtaggagaggccatctccatcagctataaccaaaaaaaaa
acaaaaaactcctctttttgacaagtttgtaaagcctgtccatctgggtc
tataataatcctccaggccctatgccactcctctttattcagccagttca
...
```

GFF file

Dotter uses the GFF version 3 file format. In this section we give a very brief description of this file format; see http://www.sequenceontology.org/gff3.shtml for a full description.

The GFF file should start with the following two comment lines. (Additional comments can be included but may be ignored.)

```
##gff-version 3
##sequence-region chr4-04 44144 154265
```

Each subsequent line defines a feature. A feature line must have the following 8 tab-separated columns:

```
reference_sequence_name source type start end score strand phase
```

An optional 9th column defines any tags (separated by semi-colons). Dotter supports the following GFF tags. (Additional tags can be supplied but may be ignored.)

**Target**	(required for alignments)
**Gap**	(required for gapped alignments)
**ID**	(required for parent features)
**Name**	(required for transcripts and SNPs)
**Parent**	(required for child features)

Transcripts

Note that exons should have a Parent transcript defined, and the Name tag should be set in the parent rather than the child exons. Note that Dotter *will* recognise exons that do not have a Parent tag if they have a Name tag instead, but they may not get grouped correctly with other exons from the same transcript.

Typically, one defines the parent transcript, the exons, and the CDS regions; Dotter will then calculate the missing components (in this case, the UTR regions and the introns). Dotter will recognise other combinations of inputs, and will always calculate the missing components as long as enough information is provided.

Sample GFF file

A sample GFF file may look like this ('...' denotes that text has been omitted).

```
##gff-version 3
##sequence-region chr4-04 44144 154265
chr4-04 EST_Human nucleotide_match 79195 79311 95.000000 - . Target=DA692754.1 \
287 403 +;percentID=90.6;sequence=GATCTGGC...
chr4-04 EST_Human nucleotide_match 79195 79323 121.000000 + . Target=AI095103.1 \
326 454 +;percentID=96.9;sequence=TTTAAATT...
chr4-04 ensembl_variation deletion 80798 80799 . + . Name=rs60725655;url=http%3A\
%2F%2Fwww.ensembl.org%2FHomo_sapiens%2FVariation%2FSummary%3Fv%3Drs60725655;vari\
ant_sequence=AA-;
chr4-04 Augustus mRNA 119534 119941 . - . ID=transcript21;Name=AUGUSTUS00000051712
chr4-04 Augustus exon 119534 119941 . - . Parent=transcript21
chr4-04 Augustus CDS 119534 119941 . - 0 Parent=transcript21
```

The Dotter Windows

The dot-plot window

The main Dotter window contains the dot-matrix plot. It also shows any exons for the sequences along the bottom of the window (for the horizontal sequence; or along the right-hand-side for the vertical sequence).

Note that the narrow red-shaded border around the edge of the plot indicates the region where the dot-plot cannot be calculated due to the sliding window averaging method that is used to calculate the scores.

Cross-hair

The blue cross-hair shows the coordinates at a particular position. It can be moved by clicking/dragging with the left mouse button, or by using the following keyboard shortcuts:

Left-arrow **Right-arrow** Move one dot left/right along the horizontal sequence.

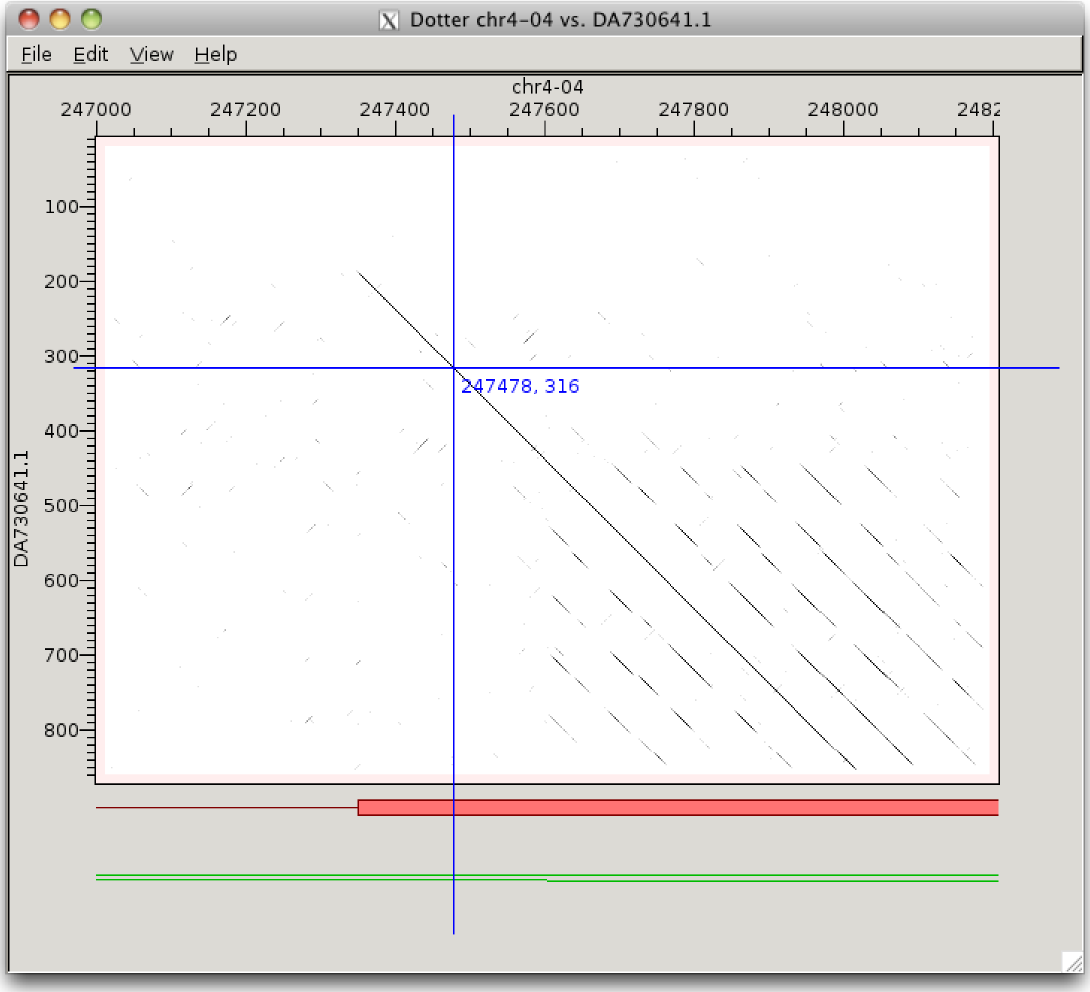

Figure 2: The main window

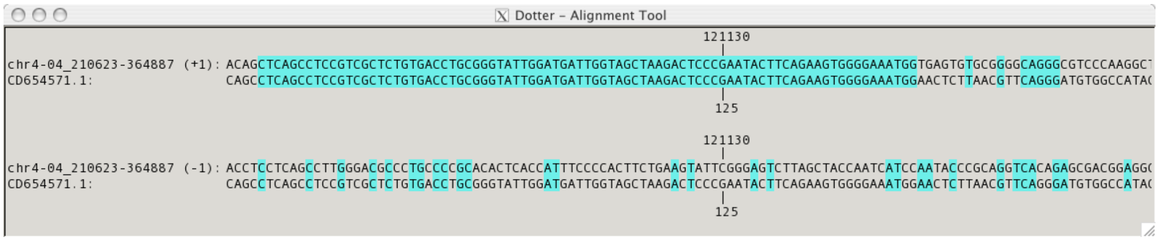

Figure 3: Alignment tool - nucleotide->nucleotide mode

**Shift-Left**<br>**Shift-Right**	The same as Left/Right, but for protein sequences this moves by a single nucleotide coordinate rather than a whole dot/amino-acid.
**Up-arrow**<br>**Down-arrow**	Move one dot up/down along the vertical sequence.
**Shift-Up**<br>**Shift-Down**	The same as Up/Down, but for protein sequences this moves by a single nucleotide coordinate rather than a whole dot/amino-acid.
**,**<br>**.**	Move diagonally up-left or down-right. Useful for moving along an alignment.
<<br>>	Move diagonally up-left or down-right but, for protein sequences, move by a single nucleotide coordinate rather than a whole amino-acid.
**[**<br>**]**	Move diagonally down-left or up-right. Useful for moving along an alignment.
**{**<br>**}**	Move diagonally down-left or up-right but, for protein sequences, move by a single nucleotide coordinate rather than a whole amino-acid.

Zoom in with a child Dotter

You can open a new child Dotter on a particular region from the current Dotter window. Middle-click and drag the mouse to select the region to open the new Dotter on.

The alignment tool

The alignment tool shows the portions of the two sequences at the current cross-hair position. The sequences will move to remain centred on the cross-hair coordinates when the cross-hair is moved. The same shortcut keys for moving the cross-hair can be used in this window.

Aligning matches are highlighted and colour-coded according to whether they are an exact or conserved match (cyan for exact, violet for conserved).

In nucleotide->nucleotide mode, both strands of the horizontal sequence are shown in the alignment tool. In nucleotide->protein mode, all three reading frames of the horizontal sequence are shown, and the best match out of the three frames determines the highlight colour for the bases in the vertical sequence.

If closed or hidden, the alignment tool can be shown with the 'Ctrl-A' shortcut or by selecting the 'Alignment tool' option under the 'View' menu.

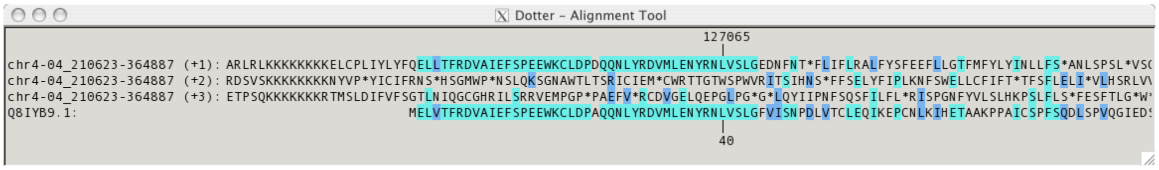

Figure 4: Alignment tool - nucleotide->protein mode

Alignment tool menu

Right-clicking in the alignment tool brings up a context menu with the following options:

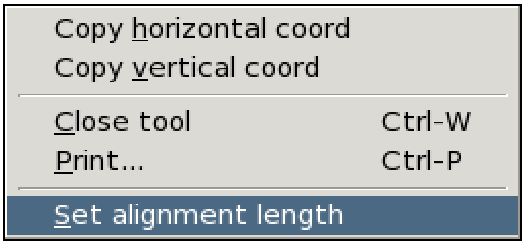

Figure 5: Alignment tool menu

**Copy horizontal coord**	Copy the current horizontal-sequence coordinate to the clipboard
**Copy vertical coord**	Copy the current vertical-sequence coordinate to the clipboard
**Close tool**	Close the alignment tool window (it can be re-opened with Ctrl-A)
**Print**	Print the alignment tool window
**Set alignment Left**	Control how long a portion of the sequences should be shown in the alignment tool.

Alignment tool shortcuts

The keyboard shortcuts for moving the cross-hair also apply in the alignment tool window.

Greyramp tool

This tool controls the threshold and contrast of the the dot-plot image. To improve visualization, little peaks (noise) can be nullified by a minimum cut-off. Similarly, significant peaks above a certain score can be saturated by a maximum cut-off.

Drag the square handle and the arrows to change the threshold and contrast. The 'Swap' button swaps the positions of the top and bottom arrows, inverting the colours. The 'Undo' button undoes the effect of the last drag.

If closed or hidden, the greyramp tool can be shown with the 'Ctrl-G' shortcut or by selecting the 'Greyramp tool' option under the 'View' menu.

Main menu

The main menu can be accessed via the menu-bar at the top of the dot-plot window or by right-clicking in the dot-plot window.

Note that menus with a dotted line at the top can be “torn off” by clicking on the dotted line. A torn-off menu will stay visible on top of the Dotter window and can be repositioned by dragging its header bar. Click the dotted line again to get rid of it.

File menu

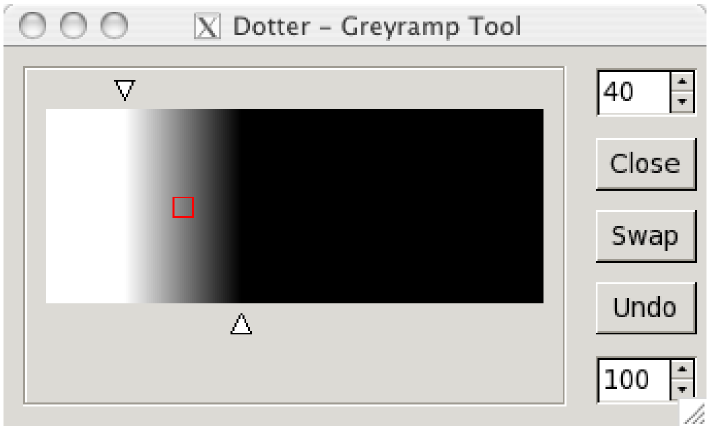

Figure 6: Greyramp tool

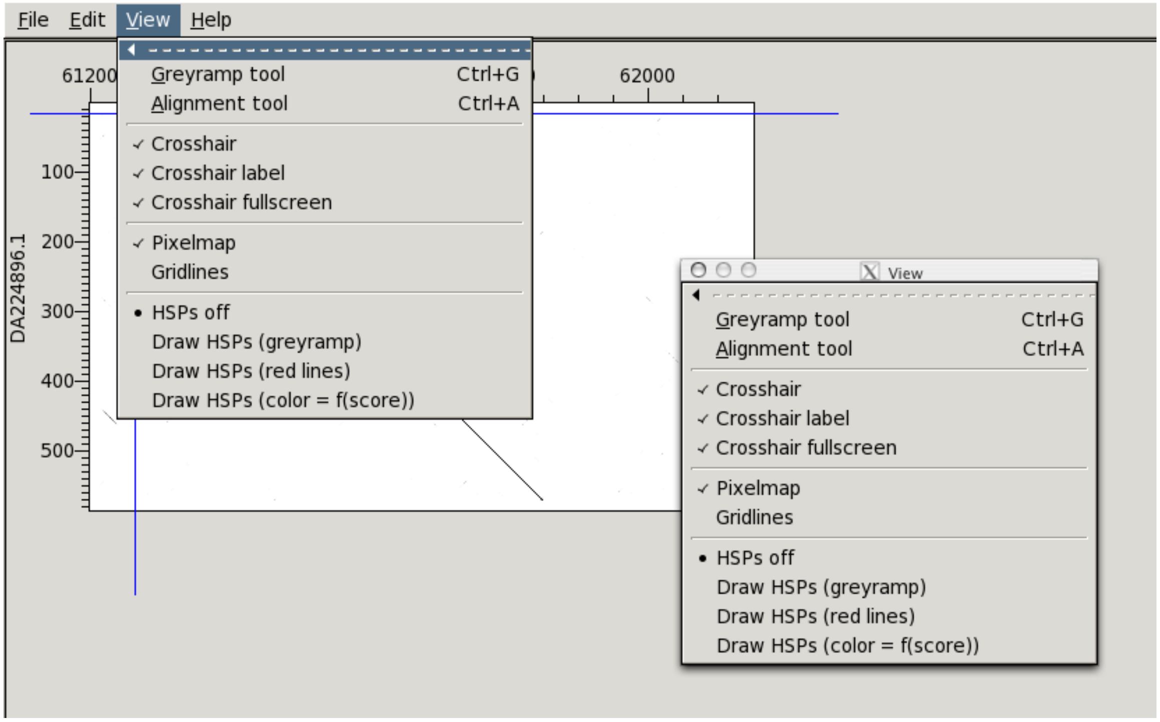

Figure 7: Tear-off menus

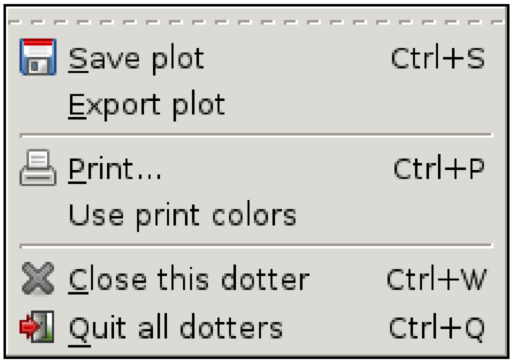

Figure 8: File menu

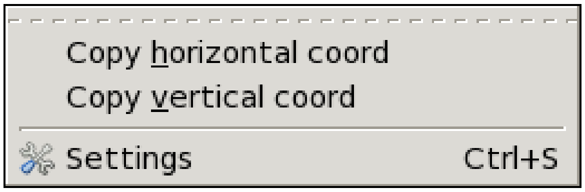

Figure 9: Edit menu

Save plot Save the current dot-plot. It can be re-loaded by calling Dotter from the command line using the -l argument. Note that you will need to call Dotter with the same portion of each sequence that was originally passed to Dotter in order for the alignment tool to function correctly when you load the dot-plot.

Export plot Export the plot to PDF format. Note that other formats are also available from the Print menu by selecting Print to File from the print dialog.

Print Print the current dot-plot.

Use print colours Change to a colour-scheme that is more suitable for printing.

Close Close the current Dotter window. Also closes the associated alignment and greyramp tool, but does not close any other Dotter windows.

Quit Close the current Dotter window and all associated Dotters as well (including any child or parent Dotters). If you just wish to close the current Dotter, then use the 'Close' menu option instead.

Edit menu

Copy horizontal coord Copy the current horizontal-sequence coordinate to the clipboard

Copy vertical coord Copy the current vertical-sequence coordinate to the clipboard

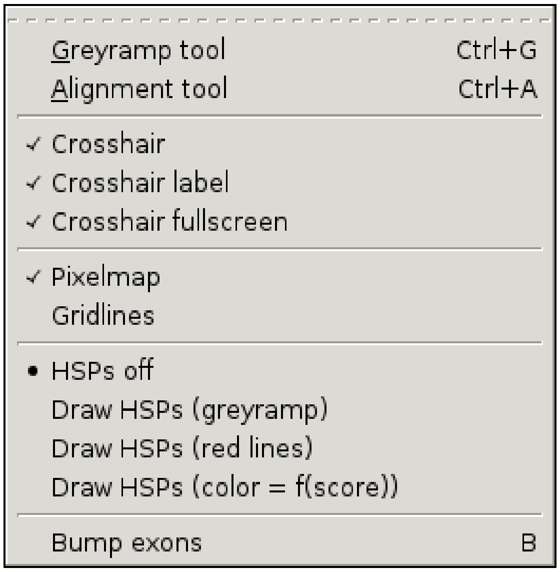

Figure 10: View menu

**Settings**	Show the 'Settings' dialog.

View menu

**Greyramp tool**	Show the greyramp tool.
**Alignment tool**	Show the alignment tool.
**Crosshair**	Toggle visibility of the cross-hair
**Crosshair label**	Toggle visibility of the cross-hair label (only has an effect if the cross-hair is visible).
**Crosshair fullscreen**	Toggle whether the cross-hair is shown to its full extents or is clipped to just the dot-plot area.
**Pixelmap**	Toggle visibility of the grey-scale dot-plot image.
**Gridlines**	Toggle visibility of gridlines.
**HSPs off**	Select this option to turn HSP (High Scoring Pair) mode off.
**Draw HSPs (greyramp)**	Select this option to view HSPs in grey-scale mode. In this mode, the HSPs (High Scoring Pairs) are drawn in a shade of grey that is determined by their score. The greyramp tool can be used to adjust the thresholds and contrast of the HSP image. This mode *replaces* the standard dot-plot image.

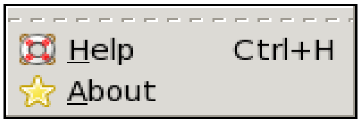

Figure 11: Help menu

Draw HSPs (red lines) Select this option to view all HSPs as red lines. This mode can be used in conjunction with the standard dot-plot image: HSPs are drawn over the top.

Draw HSPs (color=f(score)) Select this option to view HSPs as solid lines, whose colour depends on their score. This mode can be used in conjunction with the standard dot-plot image: HSPs are drawn over the top.

Bump exons Expand the transcript display so that exons do not overlap.

Help menu

Help Show the 'Help' dialog.

About Show the 'About' dialog.

Settings

The settings dialog can be accessed by selecting the 'Settings' option on the 'Edit' menu, or by pressing the 'Ctrl-S' shortcut key.

Zoom

Specify the zoom factor. The factor is an inverse: a zoom factor of 3 will zoom *out* by a factor of 3, i.e. the window will shrink to 1/3 of its full size. A zoom factor of 1 will show the window at full size. A factor of less than 1 (e.g. 0.5) can be set in order to zoom in, but this will result in a stretched dot-plot so is not recommended.

Horizontal range

Set the range of the horizontal sequence. The maximum range possible is the range that was originally passed to Dotter – the range you enter will be trimmed if you enter out-of-range values.

Note that this causes the matrix to be recalculated, so if it took a long time to calculate in the first place, stay away from this menu item!

Vertical range

Set the range of the vertical sequence. The maximum range possible is the range that was originally passed to Dotter – the range you enter will be trimmed if you enter out-of-range values.

Note that this causes the matrix to be recalculated, so if it took a long time to calculate in the first place, stay away from this menu item!

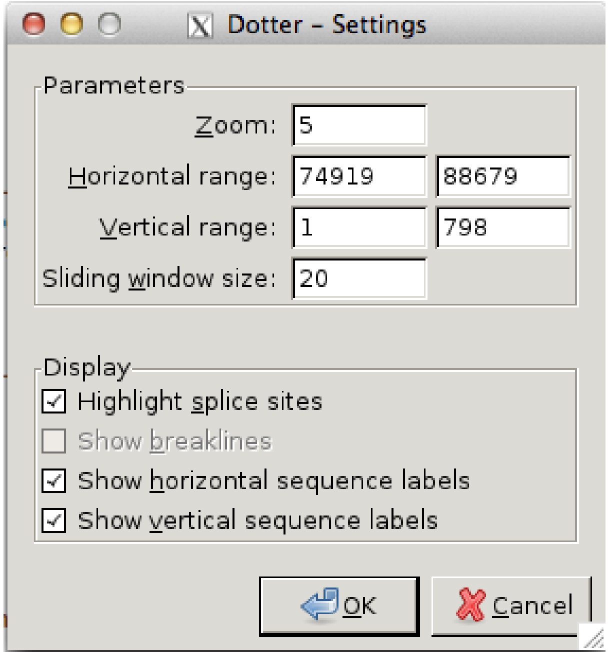

Figure 12: The Settings dialog

Sliding window size

To make the score matrix more intelligible, the pairwise scores are averaged over a sliding window that runs diagonally. This option allows you to edit the size of the sliding window. There's normally no need to change this.

Note that this causes the matrix to be recalculated, so if it took a long time to calculate in the first place, stay away from this menu item!

Highlight splice sites

When this option is enabled, splice-sites for known high-scoring pairs will be highlighted in the alignment view. The dinucleotide will be highlighted in green for a canonical splice-site and red for non-canonical.

Show break-lines

Tick this option to display break-lines between different sequences when Dottering multiple sequences (i.e. where there are multiple sequences in the same FASTA input file). This option will be greyed out if there is only one sequence per input file.

Show horizontal sequence labels

When break-lines are enabled, tick this option show labels for each break-line for the horizontal sequence.

Show vertical sequence labels

When break-lines are enabled, tick this option show labels for each break-line for the vertical sequence.

Keyboard shortcuts

**Left-arrow**<br>**Right-arrow**	Move the cross-hair one dot left/right along the horizontal sequence.
**Shift-Left**<br>**Shift-Right**	The same as Left/Right, but for protein sequences this moves by a single nucleotide coordinate rather than a whole dot/amino-acid.
**Up-arrow**<br>**Down-arrow**	Move the cross-hair one dot up/down along the vertical sequence.
**Shift-Up**<br>**Shift-Down**	The same as Up/Down, but for protein sequences this moves by a single nucleotide coordinate rather than a whole dot/amino-acid.
**,**<br>**.**	Move diagonally up-left or down-right. Useful for moving along an alignment.
<<br>>	Move diagonally up-left or down-right but, for protein sequences, move by a single nucleotide coordinate rather than a whole amino-acid.
**[**<br>**]**	Move diagonally down-left or up-right. Useful for moving along an alignment.
**{**<br>**}**	Move diagonally down-left or up-right but, for protein sequences, move by a single nucleotide coordinate rather than a whole amino-acid.
**Ctrl-W**	Close the current window. If this is a dot-plot window, it also closes the associated alignment and greyramp tool.
**Ctrl-Q**	Quit Dotter. Also quits any associated Dotters, i.e. any child or parent Dotters.
**Ctrl-S**	Open the Settings dialog.
**Ctrl-P**	Print the Dotter window.
**Ctrl-H**	Open the Help dialog.
**Ctrl-A**	Show the alignment tool.
**Ctrl-G**	Show the greyramp tool.
**Ctrl-D**	Show the main dot-plot window.
**B**	Bump exons.
