# Supplementary material for: SeqTools: visual tools for manual analysis of sequence alignments
Source: BMC Res Notes. 2016 Jan 22;9:39. doi: 10.1186/s13104-016-1847-3 (PMC4724122; doi:10.1186/s13104-016-1847-3)
Supplement: Supplementary file 1 — 10.1186/s13104-016-1847-2 A tarball of the current production release of the SeqTools source code at the time of writing. [file 13104_2016_1847_MOESM1_ESM.gz › seqtools-4.32.1/doc/User_doc/dotter_quick_start.html]

Dotter - Quick Start


# Dotter - Quick Start

- What's new
- Overview
- Mouse controls
- Keyboard shortcuts
- Settings
- Colour key

## Overview

This page gives a quick-start guide to using Dotter. Other documentation is available here:

- What's new (revision history)
- Usage (command line options)
- User manual
- Outstanding issues

## Mouse controls

- Left button: position crosshair.
- Middle button: drag to zoom in to a region.

## Keyboard shortcuts

- Arrow keys: move crosshair one dot in arrow direction. Hold Shift to move by a single nucleotide rather than a whole amino-acid (if applicable).
- , . : move crosshair along diagonals. Hold Shift to move by a single nucleotide, i.e. <, >.
- [ ] : move along reverse diagonals. Hold Shift to move by a single nucleotide, i.e. {, }
- Ctrl-Q : quit Dotter (including any child/parent Dotters)
- Ctrl-W : close the current window (if in the Dotter window, will close the related alignment/greyramp tool as well)
- Ctrl-P : print the Dotter window
- Ctrl-S : settings
- Ctrl-H : show this Help page
- Ctrl-A : show the alignment tool
- Ctrl-D : show the Dotter main window
- Ctrl-G : show the greyramp tool

## Settings

- Zoom: enter a higher value to zoom out. A value of 1 means 100%, 2 means 50% etc. A fraction of 1 can be entered in order to zoom in (e.g. 0.5 for a 200% zoom), but the display will appear stretched.
- Horizontal range: enter the min and max coords to display on the horizontal scale. Note that this will be limited to the horizontal sequence range that Dotter was started up with.
- Vertical range: enter the min and max coords to display on the vertical scale. Note that this will be limited to the vertical sequence range that Dotter was started up with.
- Sliding window size: affects cut-off limit for how dots are drawn

## Residue colours (alignment tool)

- Cyan = Identical Residue.
- Blue = Positive Score.
- No colour = Negative score.
