# Supplementary material for: SeqTools: visual tools for manual analysis of sequence alignments
Source: BMC Res Notes. 2016 Jan 22;9:39. doi: 10.1186/s13104-016-1847-3 (PMC4724122; doi:10.1186/s13104-016-1847-3)
Supplement: Supplementary file 1 — 10.1186/s13104-016-1847-2 A tarball of the current production release of the SeqTools source code at the time of writing. [file 13104_2016_1847_MOESM1_ESM.gz › seqtools-4.32.1/doc/User_doc/release_notes.html]

SeqTools - Release Notes


## SeqTools - Release Notes

#### Version 4.32

- Blixem
  - Previously-unsupported GFF feature types can now be displayed as a new 'basic feature' type.- Styles are now applied to the detail view as well as the big picture, so feature display is more consistent across the two views.- Fix a bug where styles were not being applied to dynamically-loaded features.- Dotter
    - Added a 'maximise' button for the greyramp tool.- Small fixes to inconsistencies in context-sensitive menus and keyboard shortcuts.

#### Version 4.31

- All
  - Fix configure error on Cygwin on Windows 7.- Make dependency on sqlite optional.

#### Version 4.30

- Blixem
  - Fix potential crash with internal processing- Fix two bugs in data input- Dotter- Belvu

#### Version 4.29

- Blixem
  - Added the facility to run Dotter on an ad-hoc sequence (RT:408836).- Added the facility to run Dotter against a transcript.- Dotter
    - Minimise the greyramp tool to a simple contrast slider. Use Ctrl-G to toggle between this and the full greyramp tool.- Belvu
      - Fix a bug with tree bootstrapping in Belvu.

#### Version 4.28

- Blixem
  - Fixed a bug where Blixem would crash if you drag-and-droped a pseudogene into it from ZMap. (RT:407536)- Store original as well as upper-cased feature names to solve a lookup problem when fetching sequence data (RT:408965)- Dotter
    - Fixed a bug in Dotter where one of the coord labels on the alignment tool is not centred after resizing the window.

#### Version 4.27

- Blixem
  - You can now drag and drop features into Blixem. The dragged data must be text in GFF3 format (RT234245).- Dotter auto-coords now uses the big picture range to make it more consistent (RT131802).- Dotter
    - Dotter's windows are now dockable. You can toggle docKing with Ctrl-K, and toggle the Alignment and Greyramp tool visibility with Ctrl-A and Ctrl-G respectively (RT398273).- Splice sites are now highlighted in the alignment tool for known HSPs (RT335980).- Fix a crash with showing HSPs.

#### Version 4.26

- Blixem
  - Add lines to show colinearity between alignment blocks.

#### Version 4.25

- Blixem
  - Add support for fetching sequence data from SQLite databases.

#### Version 4.24

- Blixem
  - Add support for a "cigar\_bam" attribute in GFF, which contains a BAM-style cigar string.

#### Version 4.23

- Blixem
  - Minor change to use the GFF Gap string in preference to the new cigar\_bam attribute (which isn't fully implemented yet)

#### Version 4.22

- Blixem
  - Add new config options for linking and squashing features- Add a Clear button to the Find dialog- Work-in-progress to add support for a cigar\_bam attribute in the GFF- Dotter
    - Make sure dotter doesn't try to use X if it's not required

#### Version 4.21

- Blixem
  - Splice-site highlighting is enabled by default- Improved support for small screens- New colour scheme (RT329278)- Dotter
    - Add command-line arguments to specify sequence types (so Dotter does not have to guess)

#### Version 4.20

- All
  - Minor internal robustness improvements

#### Version 4.19

- All
  - Usage/version/help info are now printed to stdout, with successful exit code

#### Version 4.18

- Blixem
  - Add option to abbreviate window title prefixes- Improve coverage view scale and labelling

#### Version 4.17

- Blixem
  - Copy match/reference sequence to the clipboard from the right-click menu- Allow loading of non-native file formats using fetch methods- Dotter
    - Fix crash on long slices- Fix intermittent label rendering on long slices

#### Version 4.16

- All
  - Fixed a bug where the link in the About dialog did not work.- Include the following font in the preferences list when searching for a fixed-width font: DejaVu Sans Mono- Blixem
    - Fixed a bug where Blixem was passing incorrect values in the %s and %e substitution variables for fetch method arguments (RT286051).- Dotter
      - Fixed a bug where the sequence labels in the Alignment tool were truncated.- Removed deprecated drawing functions from Dotter (might address RT281848: intermittent problems with drawing axis labels).

#### Version 4.15

- Blixem
  - Fix a bug where blixem column label text overlaps- Fix a bug where the source column is sometimes blank- Make sure all stdout messages are flushed- Improved error handling: check executables are in the path before trying to call them

#### Version 4.14

- Blixem
  - Sequence-fetching has been generalised so that it can now be used by users outside the Sanger. Data sources can be specified in the config file.- The config file can now be used to specify whether features with the same name should be linked under the same parent.- Fix an error that can occur when opening the help page in Firefox.- Belvu
    - Added keyboard shortcuts for Make-non-redundant (Ctrl-R) and Remove-partial-sequences (Ctrl-T)

#### Version 4.13.2

- Blixem
  - Fixed a compilation error on Ubuntu 12.04.

#### Version 4.13.1

- Blixem
  - Fixed a bug where features were not being linked under the same parent correctly.

#### Version 4.13

- Blixem
  - Fixed a bug where the --squash-matches argument was being overridden by the config file.- Dotter
    - You can now select and copy sequence from the alignment tool using the mouse.- Fixed a bug where the coordinates in the alignment tool were wrong if you set the display length to an even number.- Fixed an issue where the greyramp tool had the wrong context menu.- Fixed some bugs with printing: the alignment tool labels were not printed correctly; Ctrl-P on the alignment tool printed the wrong window; the background colour was not white in the print; the background colour in the alignment tool was getting messed up after a print operation.

### Version 4.12

- Blixem
  - Performance improvements when reading in large GFF files.- Short reads are now counted as duplicates in the 'squashed' view even if they are from different sources.- The detail-view now scrolls the selected match into view when you jump to the next/previous match. This also now works when matches are squashed.- Fixed some bugs with handling of short read data that was causing some reads to appear misaligned.- Fixed a bug where the sort order was incorrect for short reads.- Fixed a bug in protein mode where the variations track would flicker, and you were unable to select features in the variations track.- Removed duplicate sequence data from the sample GFF files.

### Version 4.10.1

- Blixem
  - Added examples directory- Improvements to test scripts- Fixed incorrect return value from Blixem's main function

### Version 4.10

- Blixem
  - Display settings are now saved on exit.- Dotter
    - Fixed a bug where exons were not always being displayed.- Fixed a bug where Dotter would fail when comparing a very long horizontal sequence to a short vertical sequence (RT:242777).- Fixed an issue where you could not scroll to the very edge of the plot (RT:251308).- Belvu
      - Fixed a crash when running in non-graphical mode.

### Version 4.9

- Blixem
  - Fixed a bug from version 4.7 where blixem would crash if no config file was given.- Fixed a bug in Blixem where Dotter would fail to start after opening the Blixem help page.- General
    - Fixed some memory leaks.

### Version 4.8.1

- Blixem
  - Fixed a buffer overrun bug introduced in version 4.7.

### Version 4.8

- Blixem
  - Improvements to installation scripts.- Updated user manuals for changes since version 4.2.- Fixed an issue where Blixem would fail to start if a config file was given but did not contain pfetch groups.

### Version 4.7

- Blixem
  - Overlapping items in the variations track are now drawn on separate lines rather than being drawn over the top of each other.- Split codons are now highlighted in exons in the detail view.- Improvements to navigation in the big picture: double-clicking/dragging now scrolls the selected range (like middle-clicking/dragging); left-clicking on the highlight box and dragging allows it to be moved; the cursor now changes when dragging the highlight box; and the horizontal mouse wheel now works when over the transcript view.- If you change column visibility or sizes, these settings are now remembered when you close and re-open Blixem. You can revert to the default column visibility and sizes using the 'Reset to defaults' button on the Settings dialog.- Dotter
    - Fixed an issue with printing where only the visible part of the scrolled window was printed.- Added the ability to export plots to PDF. In batch mode, either the dot-matrix or the PDF, or both, can be saved.

### Version 4.6

- Blixem
  - Made the main window title more informative.- Exons can now be selected by clicking in the big picture, and are sorted in the same order as the detail view.- Dotter
    - The transcript sections can now be bumped.- The area near the border where alignments cannot be calculated (due to the sliding window calculation) is now shaded in red.

### Version 4.5

- Blixem
  - Blixem now highlights regions in the reference sequence that are gaps (RT:225707)- Blixem now handles gap characters in the reference sequence (RT:226652)- Fixed a bug where multiple UTRs in the same exon are not displayed (RT:225220)  
  - Belvu
    - Fixed a bug with the last column not being fully shown.- Fixed a bug with columns not being resized correctly after deleting sequences.- Made sure all terminal messages are terminated with a line break.- Added `BELVU_STATUSBAR_SIZE` environment variable to allow the statusbar to have a different font size to the main window. (Set this to zero to hide the statusbar.)- Fixed an issue where the fetch and annotations windows were not using the font size specified by `BELVU_FONT_SIZE`.- Fixed a bug where the wrong colour scheme was selected in the menu when loading a residue colour scheme on start-up.- Improved the size of the fetch window.- Fixed a performance issue when reading in files with many annotation lines.

### Version 4.4.1

- Belvu
  - Fixed a potential crash when opening the tree window if the user scrolls the mouse-wheel at the same time.- Fixed an issue where user could not re-select the sort-by-similarity/id option after changing the selection.- Added a fix to make sure tree cannot get so large that it causes a crash.- Fixed a bug with sorting by similarity on start up when the first line is a markup line.

### Version 4.4

- Blixem
  - There is a new --zoom-range argument, which allows the caller to specify the initial big picture range.- Reference sequence coordinates can now be specified in the FASTA definition line in the sequence file; the line format must be:   `>name start end`- Fixed a bug where introns that are partially out of range were not being shown.- Fixed a bug where short reads were incorrectly being squashed onto the same line in 'squash matches' mode when their sources are different.- Fixed a bug where the initial exon view height was sometimes being calculated incorrectly.- Fixed a bug where starting Dotter fails if the fetch command returns a string such as "no match".  
  - Belvu
    - Added Belvu to the SeqTools package.- The functionality to left-click a menu to get a default action has been removed; left- or right-clicking a menu will now always show the drop-down menu. The default left-click actions have been moved to a toolbar adjacent to the menu; hover over the toolbar icons for tooltips.- There is a new status bar at the bottom of the window; look out for feedback and helpful hints here.- The color menu now only contains the color schemes; the other settings from this menu have been moved to the new Settings menu.- You can quickly toggle between color-by-residue and color-by-conservation modes using the keyboard shortcut 't'.- The 'toggle last palette' menu option has been removed because its original purpose (of being able to switch back to a custom color scheme) was not clear. There is now a 'custom' entry in the color scheme sub-menu that you can use for this purpose. (Note that this only applies to color-by-residue mode.)- There are some new keyboard shortcuts - see the quick start guide for details.- You can now control the text size of the alignments using the zoom buttons on the toolbar. You can change the text size of the menus using the `BELVU_FONT_SIZE` environment variable (e.g. `setenv BELVU_FONT_SIZE 14`). NB To change the font size for all GTK applications, use the `~/.gtkrc-2.0` file instead (e.g. `gtk-font-name="sans serif 14"`).- You can now search for an alignment by name using the Find button on the toolbar (or the Ctrl-F shortcut key).- You can "tear off" drop-down menus using the dotted line at the top.- You now get a different cursor when you are in the remove-many-sequences mode, rather than a dialog box that you must close to exit the mode; you can now exit this mode by deselecting the option in the toolbar or menu; by pressing the 'Esc' key; or by left-clicking anywhere in the alignment window.- When you edit the alignment, the tree becomes invalidated and must be recalculated using the 'Recalculate tree' option on the File menu or right-click menu.- If you change a tree by clicking a branch to swap nodes/re-root the tree, then the current tree window will be updated (rather than creating a new tree window, as in the old Belvu). If the alignment is sorted by tree order, then it will be updated to reflect the new tree. To revert to the original tree, you can recalculate it using the 'Recalculate tree' option on the File menu or right-click menu.

### Version 4.2

- Blixem
  - Blixem now supports fetching of short-read data from BAM files.- Blixem command-line arguments have changed.

### Version 4.1.14

- Blixem
  - Short reads: The 'short\_read' feature type is now supported.- Coverage: A new 'coverage view' has been added to show coverage depth - it can be enabled via the View dialog.- Sorting: The sort-by drop-down box has been replaced with a Sort dialog, allowing sorting by multiple columns - open it with the Sort button on the toolbar. The 'invert sort order' option has been moved from the Settings dialog to the Sort dialog.- Search by column: When grouping or finding sequences, you can now search on data in different columns, not just the Name column. See the Group and Find dialogs.- Groups: You can now hide all sequences that are not in a group. See the Groups dialog.  
  - Dotter
    - The 'short\_read' feature type is now supported.- The right-click menu has been shortened by placing the display options into a sub-menu.

### Version 4.1.13

- Blixem
  - Replaced the Help dialog with HTML help pages.  
  - Dotter
    - Fixed some bugs with scrolling in the alignment tool.- Replaced the Help dialog with HTML help pages.

### Version 4.1.12

- Blixem
  - You can now change the active reading frame by clicking in a list header (as well as in the list itself).

### Version 4.1.11

- Blixem
  - The 'Source' column is now enabled by default. It is also sortable and its contents are now shown in the feedback area.- Column widths can now be specified in the Blixem config file (which is supplied via the `-c` command line argument).  
  - Dotter
    - Fixed an issue where Dotter was overwriting the primary clipboard contents on startup.

### Version 4.1.10

- Blixem
  - Fixed a bug with populating optional data for protein variants.

### Version 4.1.9

- Blixem
  - Printing now scales to fit the page.- Fixed a bug where splice sites were incorrectly being shown at termini.  
  - Dotter
    - You can now print from Dotter.- There is now an exon view for the vertical sequence.- There are now separator lines between sequences when Dottering multiple sequences.

### Version 4.1.6

- Dotter
  - You can now copy coordinates from the alignment tool using the right-click menu.- Re-added the exon view for the vertical sequence.

### Version 4.1.5

- Blixem
  - Close all Dotters: There is a new menu option to close all Dotters started from the current Blixem. Right-click in Blixem to see the menu.  
  - Dotter
    - Dotter re-vamp: Dotter has been re-written to facilitate future improvements. Most of the changes so far are under-the-hood, but you will notice a few cosmetic differences and additional shortcuts.- Menu bar: As well as the right-click menu, there is now a menu-bar at the top of the main Dotter window.- CDS/UTR regions: Exons are now separated into CDS and UTR regions: CDS regions are coloured green and UTR red.- Close all sub-Dotters: You can close all related Dotter windows using the Quit menu option or Ctrl-Q. This will close all sub-Dotters that were started under the same parent Dotter (by middle-dragging to zoom in to a region). To just close an individual Dotter window, click on the x in the corner of the window or use your system shortcut for closing a window (e.g. Ctrl-W, or Cmd-W on Macs). Note that the alignment tool and greyramp tool will be destroyed along with their parent - however, if the parent window is still open then these tools can be re-opened using the relevant menu options or keyboard shortcuts.- Close all Dotters from Blixem: All Dotter windows spawned from a Blixem will be closed when that Blixem is closed.- Navigation keys: You no longer need to hold Shift when moving diagonally along an alignment using the keys ',.[]'. You can hold down Shift to move a single nucleotide at a time along a protein sequence, rather than moving a whole amino-acid.- Keyboard shortcuts: The following keyboard shortcuts have been added to show the Alignment tool, main Dotter window or Greyramp tool, respectively: Ctrl-A, Ctrl-D and Ctrl-G.- Settings dialog: The Parameter Control dialog box has been replaced by a more intuitive Settings dialog. From here you can change the zoom or edit the display range.

### Version 4.1.4

- Blixem
  - Variations: SNPs, insertions, deletions etc. can be highlighted in the reference sequence, and shown in a 'variations track' above the reference sequence. See the Settings dialog or double-click the reference sequence to show/hide the variations track. Double-click a variation to open its URL. This is an experimental feature and the display is quite crude - please give us feedback on how you would like variations to be displayed, particularly where they overlap.- PolyA tails: PolyA tails can be shown and polyA signals highlighted in the reference sequence. See the Settings dialog.- CDS/UTR regions: Exons are now separated into CDS and UTR regions: CDS regions are coloured green and UTR red. Don't forget, you can quickly bump/un-bump the exon view by pressing 'b'.- Moused-over item feedback: There is now an additional feedback area on the toolbar that shows information about the currently moused-over item.- Status bar: There is now a status bar at the bottom of the window, which shows non-critical messages and warnings.- Grid scale: The grid scale now has a finer level of granularity; you can set %ID per cell to as low as 0.1 (see the Settings dialog).  
  - Dotter
    - New Dotter: Dotter has had a re-vamp - check the Dotter Help page for more info.
