# Supplementary material for: SeqTools: visual tools for manual analysis of sequence alignments
Source: BMC Res Notes. 2016 Jan 22;9:39. doi: 10.1186/s13104-016-1847-3 (PMC4724122; doi:10.1186/s13104-016-1847-3)
Supplement: Supplementary file 1 — 10.1186/s13104-016-1847-2 A tarball of the current production release of the SeqTools source code at the time of writing. [file 13104_2016_1847_MOESM1_ESM.gz › seqtools-4.32.1/doc/User_doc/todo.html]

SeqTools - To Do


## SeqTools package to-do lists

### Blixem

- Issues
  - Variations: the variations track is experimental code and will need to be improved, in particular in its handling of overlapping features.- Feature-series: this functionality was disabled when Blixem and Dotter were re-written at version 4. We should consider whether this is still a useful feature and, if so, whether to use the original SFS file format or to switch to GFF. The SFS file format is documented here.- Multiple row selections: using the up/down arrow keys to move the row selection doesn't always work if there are adjacent rows from the same match sequence.- DNA search: this currently just jumps to the start of the DNA section that was found; we should also highlight the found DNA in the reference sequence.- Printing: the pfetch window should have a print option.  
  - Enhancements
    - It would be nice to be able to view the sequence data for an insertion (a vertical yellow line in a match sequence) in Blixem, e.g. by hovering over it with the mouse.- When you hover over an exon in the exon view in Blixem (when bumped), it would be nice if info about the exon were shown in the toolbar feedback area.- When running Dotter on a large range it takes a long time to start up and there is no indication that anything is happening; it would be good to have a status bar of some description, and a cancel button. When running from Blixem, we should report if Dotter fails to start.- When calling Dotter from Blixem, start Dotter with the crosshair placed at the currently-selected coords in Blixem (if applicable).- Cosmetic: the %ID labels on the big picture grid should be right-aligned.- Button tooltips: the zoom buttons in the big-picture should have tooltips.

### Dotter

- Issues
  - There is a bug where the dot-plot can appear blacked-out if the plot is very wide and you zoom in (say to about half of the default compression factor).  
  - Enhancements
    - If you hit the boundary of one sequence when moving along a diagonal in Dotter and continue to press the movement keys (,.[]), then it stops moving on the one sequence but continues to move along the other, i.e. it goes off the diagonal. It would be nicer if it stopped moving altogether.

### Belvu

- Issues
  - Printing: the pfetch window should have a print option; so should the Annotations window.- In the colour-by-conservation colour schemes, the foreground colour is tied to the background colour. This means that if the same background colour is specified more than once, the foreground colours are ambiguous.- Colours dialog: the dialog for editing the residue colours used to have a section at the bottom showing residues grouped by colour. This has not been implemented in the new Belvu yet.- For short alignments, the initial window size is too large.  
  - Enhancements
    - Undo function (RT:214316).- Search-for-residues function (RT:212364).
